# Supplementary figures and images for: Persistent cell migration emerges from a coupling between protrusion dynamics and polarized trafficking
Source: eLife. 2022 Mar 18;11:e69229. doi: 10.7554/eLife.69229 (PMC8963884; doi:10.7554/eLife.69229)

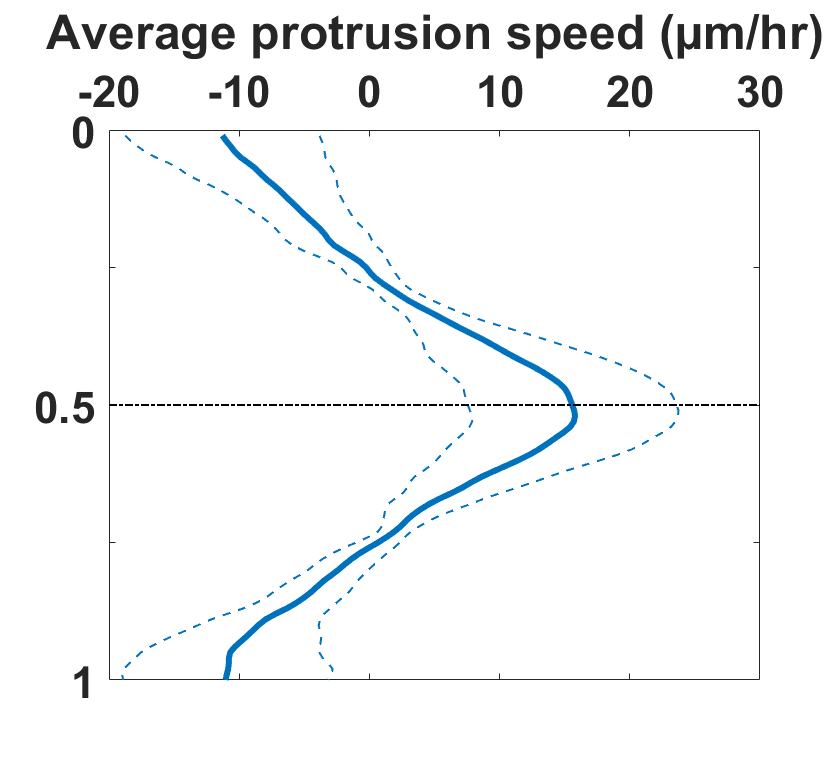

Supplement: Figure 1—source data 1. [file elife-69229-fig1-data1.zip › Figure1 Supp/1c-d/protrusion_Traj_centered_mean_std_um-h_line_FLIPPED.png]

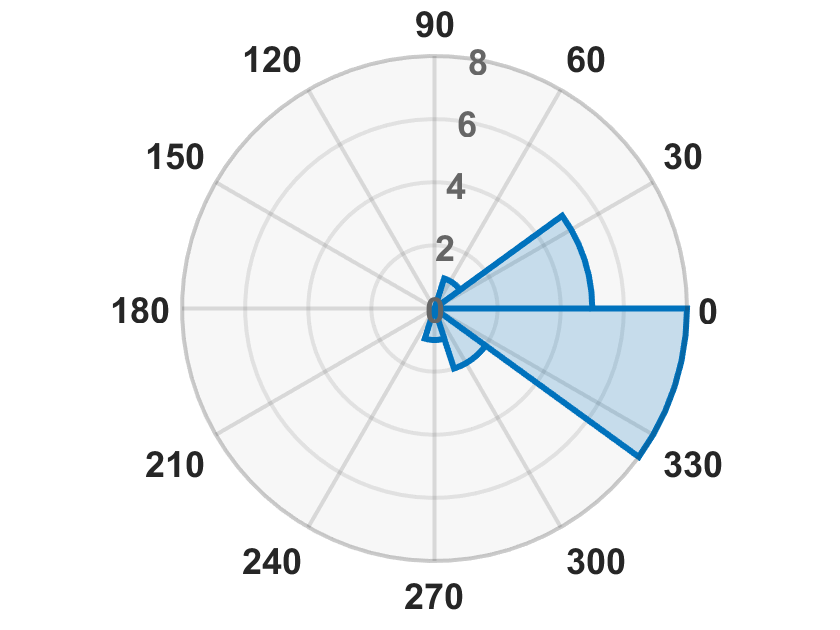

Supplement: Figure 1—source data 1. [file elife-69229-fig1-data1.zip › Figure1/1c/averaged_angles_RPE1_18.png]

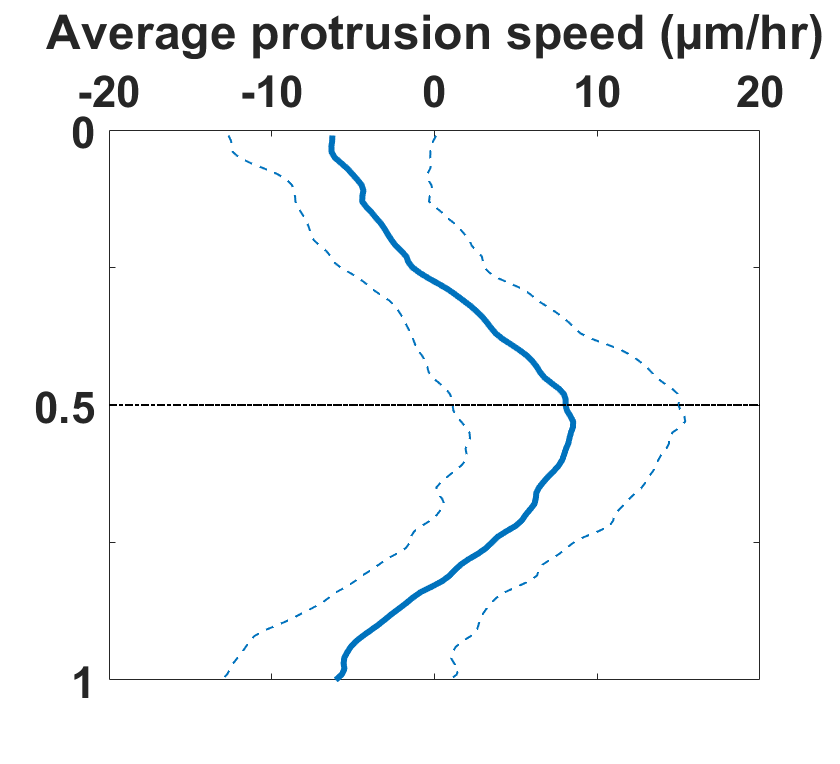

Supplement: Figure 1—source data 1. [file elife-69229-fig1-data1.zip › Figure1/1f/protrusion_NG_centered_mean_std_um-h_line_FLIPPED.png]

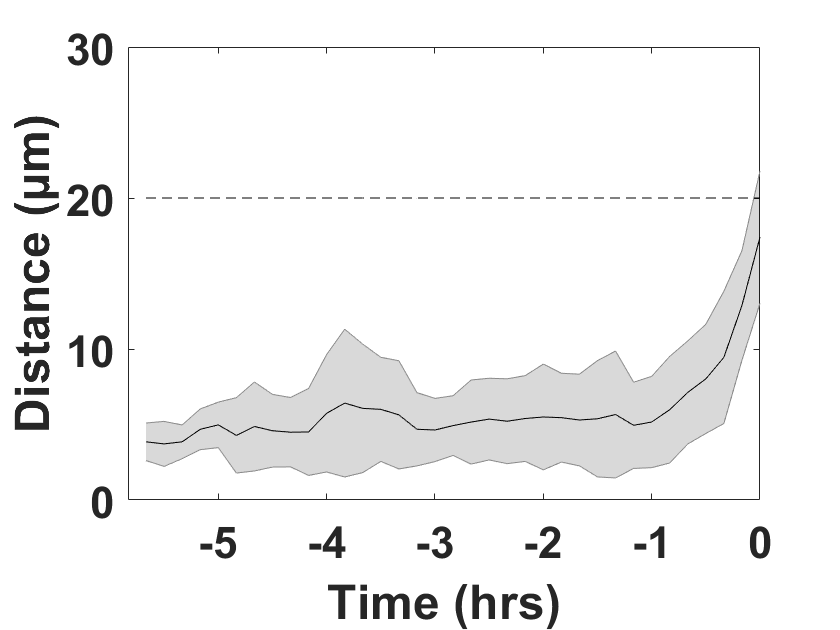

Supplement: Figure 2—source data 1. [file elife-69229-fig2-data1.zip › Figure2 Supp/Figure2 supp2 A-B/escape_Nucleus_22.png]

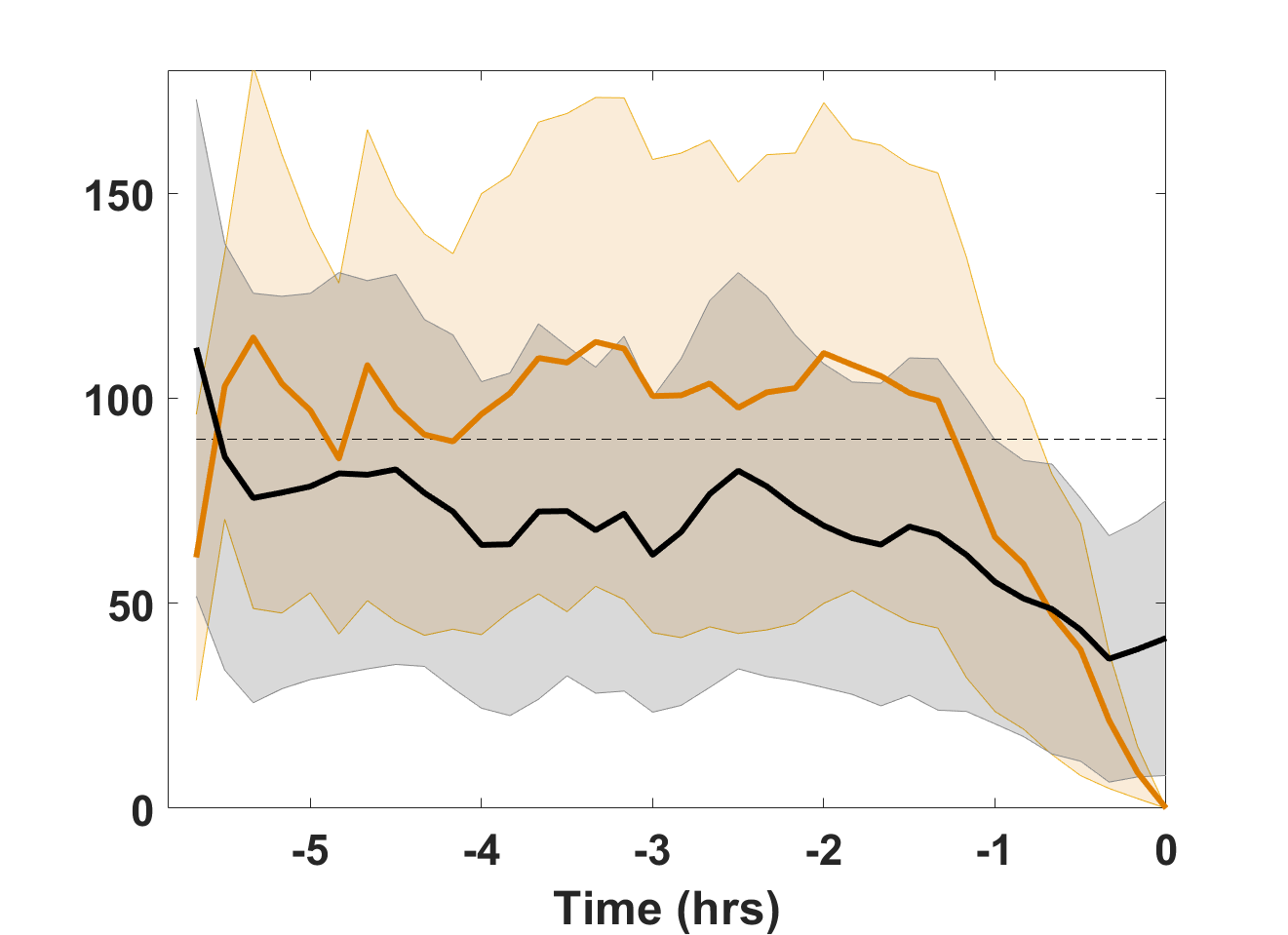

Supplement: Figure 2—source data 1. [file elife-69229-fig2-data1.zip › Figure2 Supp/Figure2 supp2 A-B/normalized_N-pattern_escape_Angles_BO_22.png]

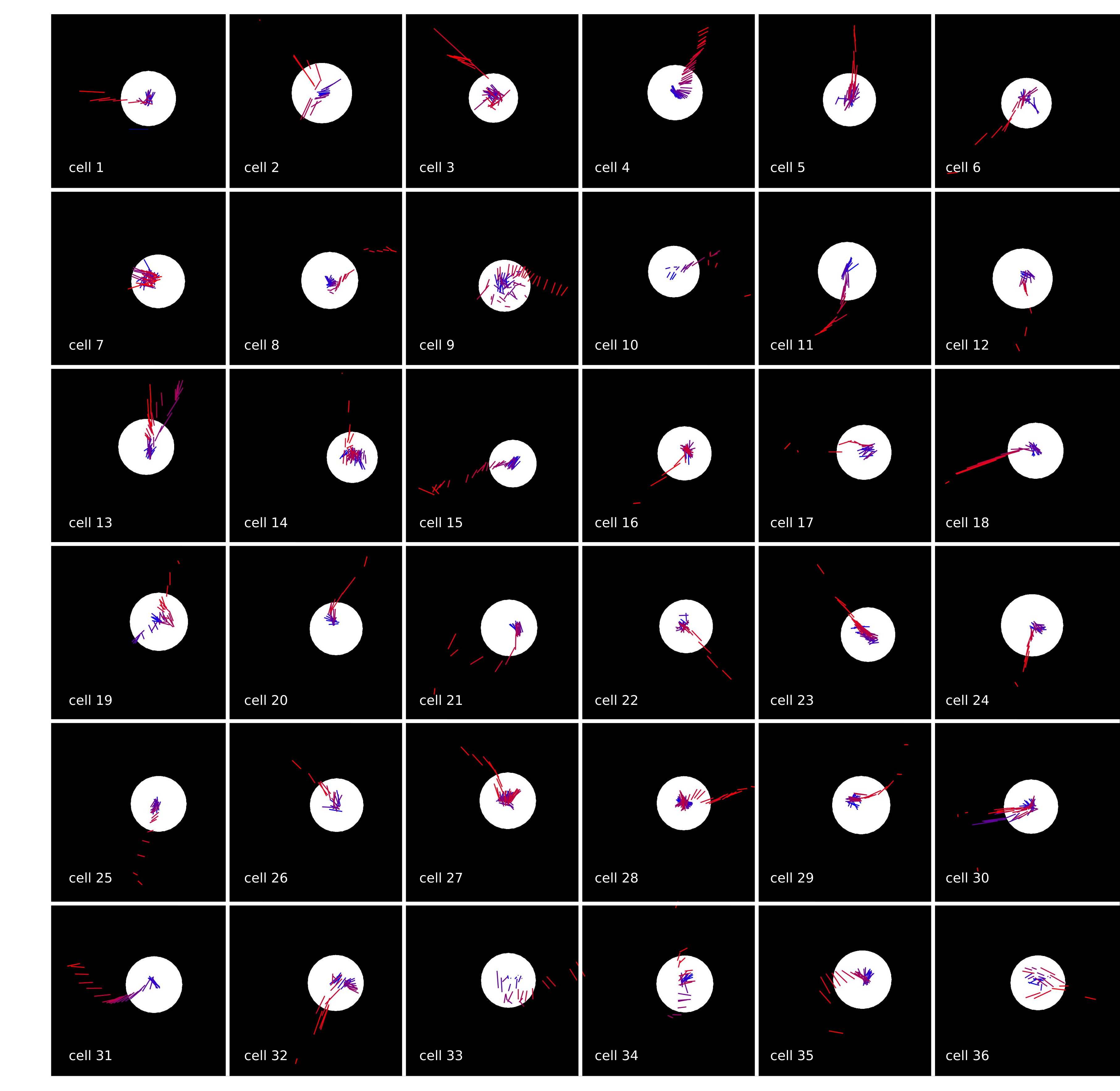

Supplement: Figure 2—source data 1. [file elife-69229-fig2-data1.zip › Figure2 Supp/Figure2_supp1.png]

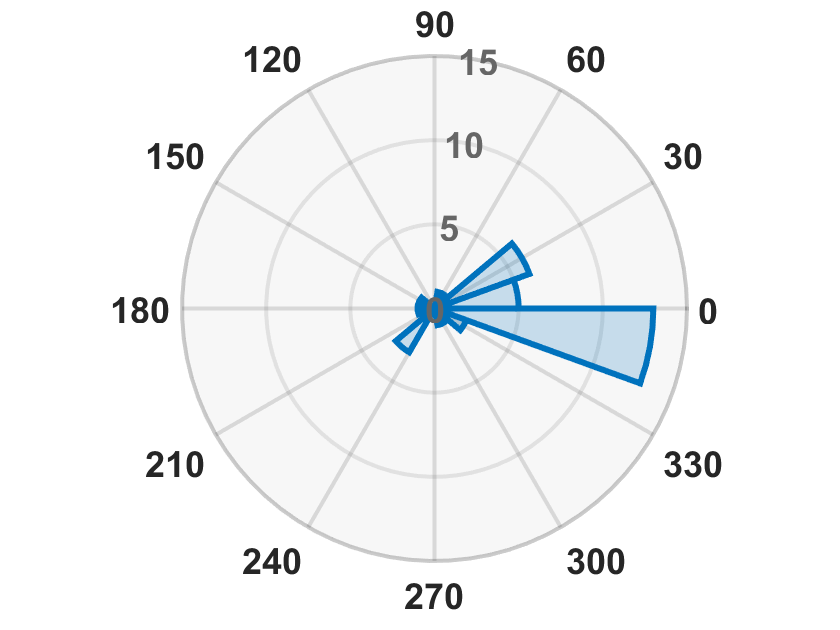

Supplement: Figure 2—source data 1. [file elife-69229-fig2-data1.zip › Figure2/2c-d/escapeTescape_rose_fin_max.png]

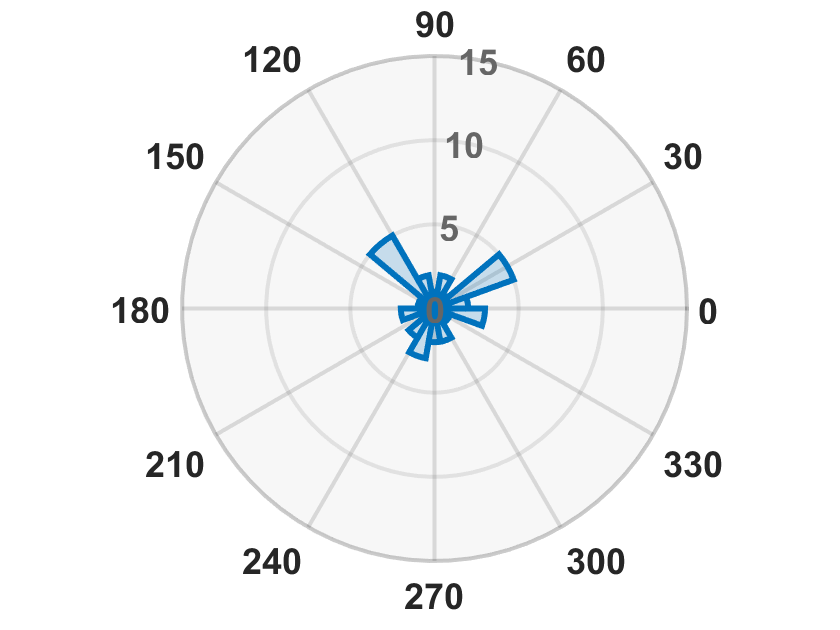

Supplement: Figure 2—source data 1. [file elife-69229-fig2-data1.zip › Figure2/2c-d/escapeTzero_rose_fin_max.png]

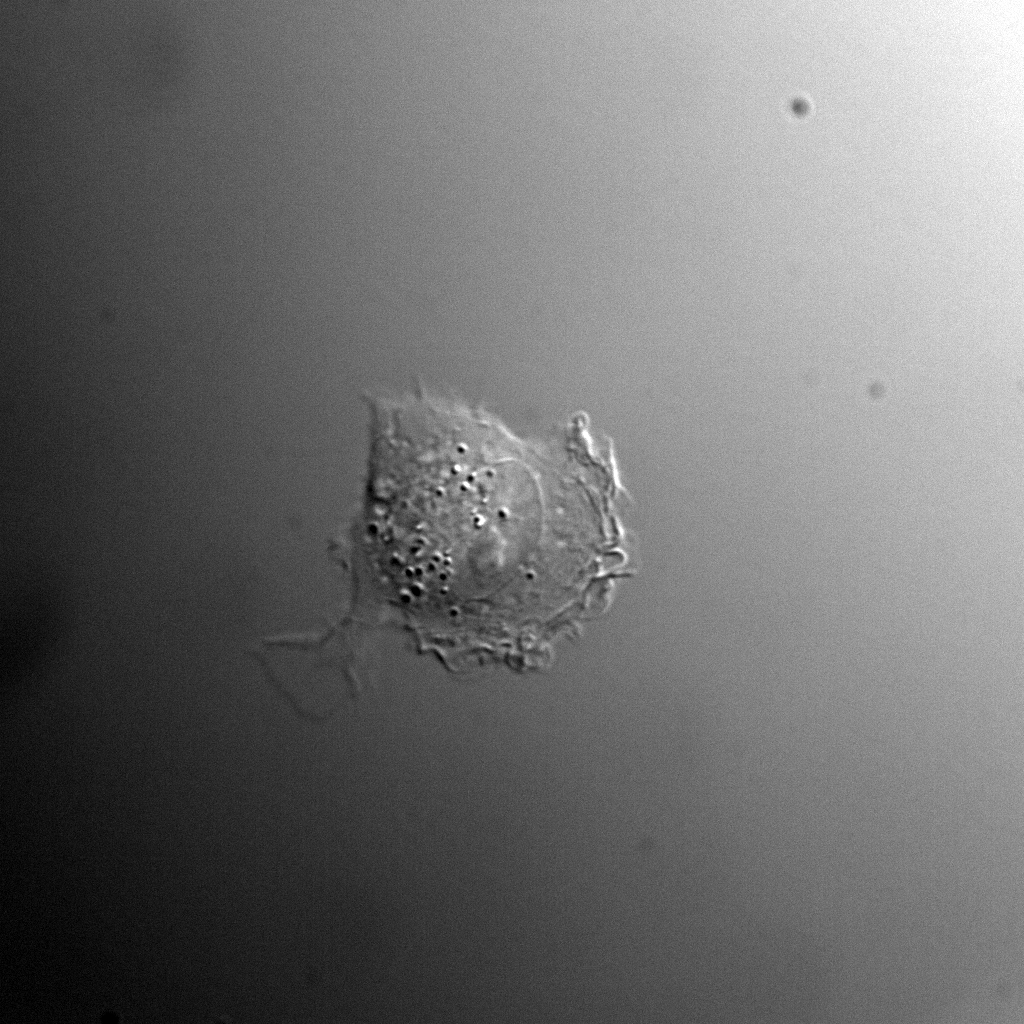

Supplement: Figure 2—source data 1. [file elife-69229-fig2-data1.zip › Figure2/scripts for escape from pattern data extraction/TRACKNUC/cell.tif]

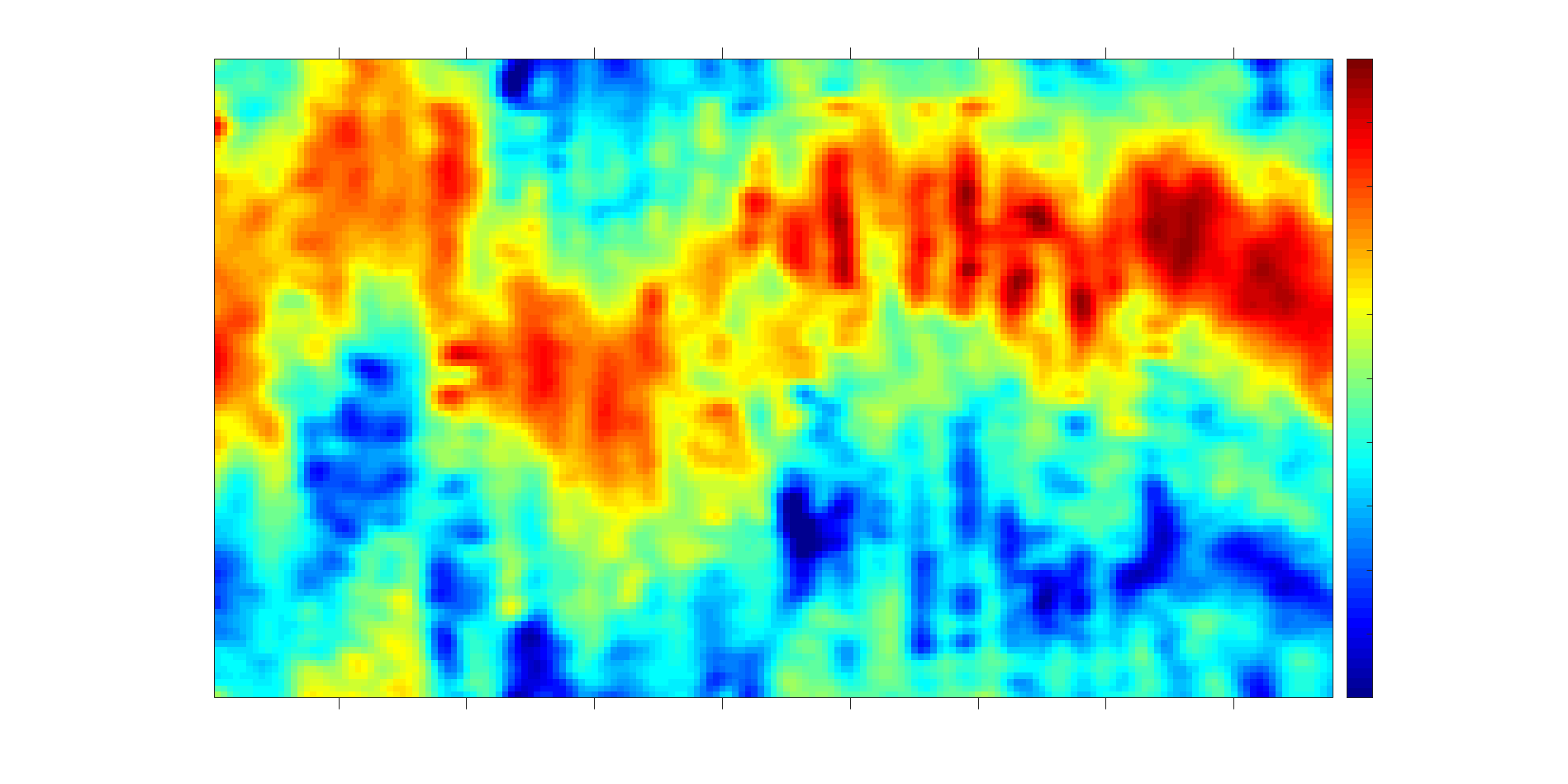

Supplement: Figure 3—source data 1. [file elife-69229-fig3-data1.zip › Figure3 Supp/control/c012_pos2_morpho_map_noshift_Xaxis_noLabel_25.png]

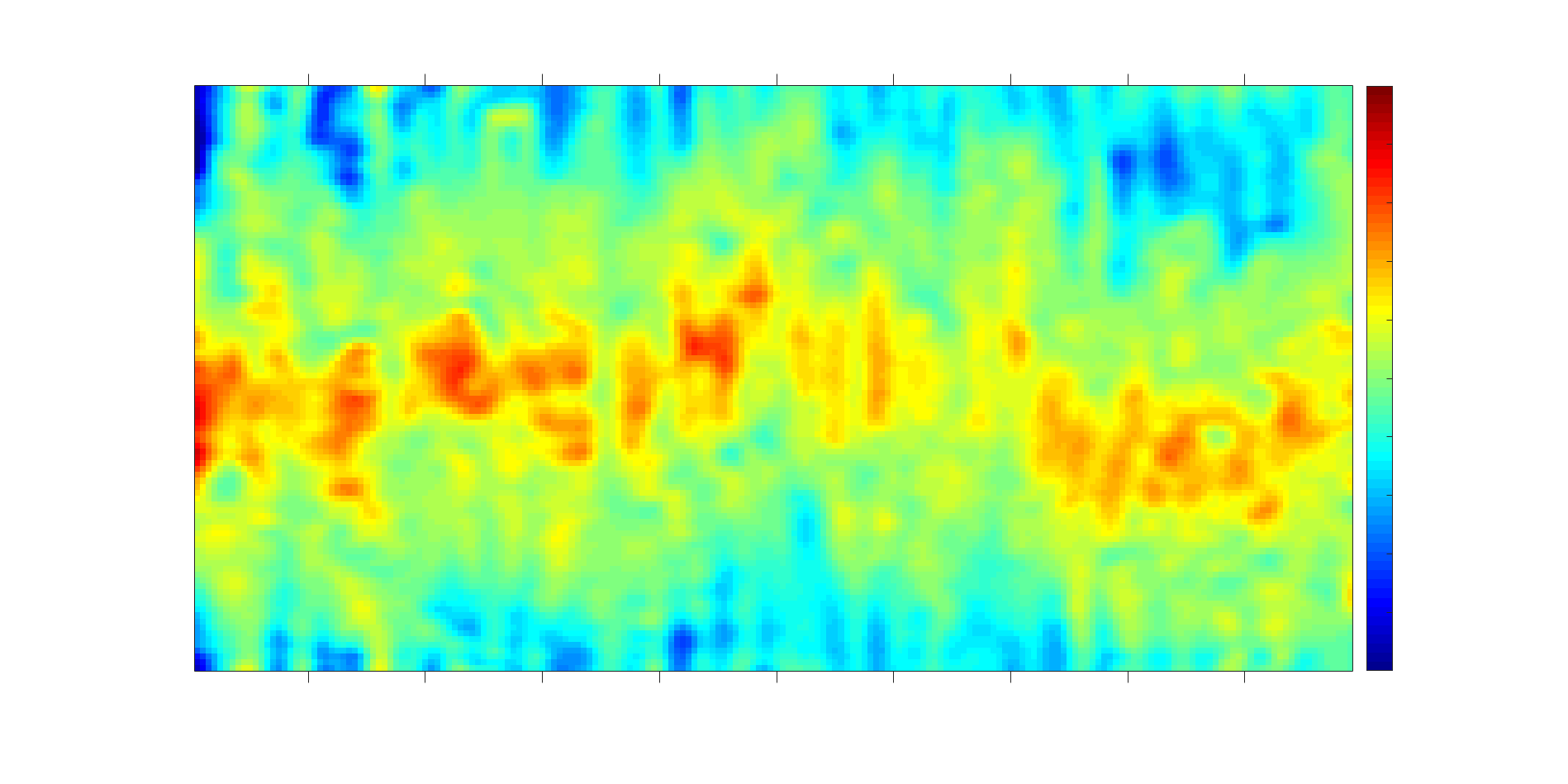

Supplement: Figure 3—source data 1. [file elife-69229-fig3-data1.zip › Figure3 Supp/control/c012_pos6_morpho_map_noshift_Xaxis_noLabel_25.png]

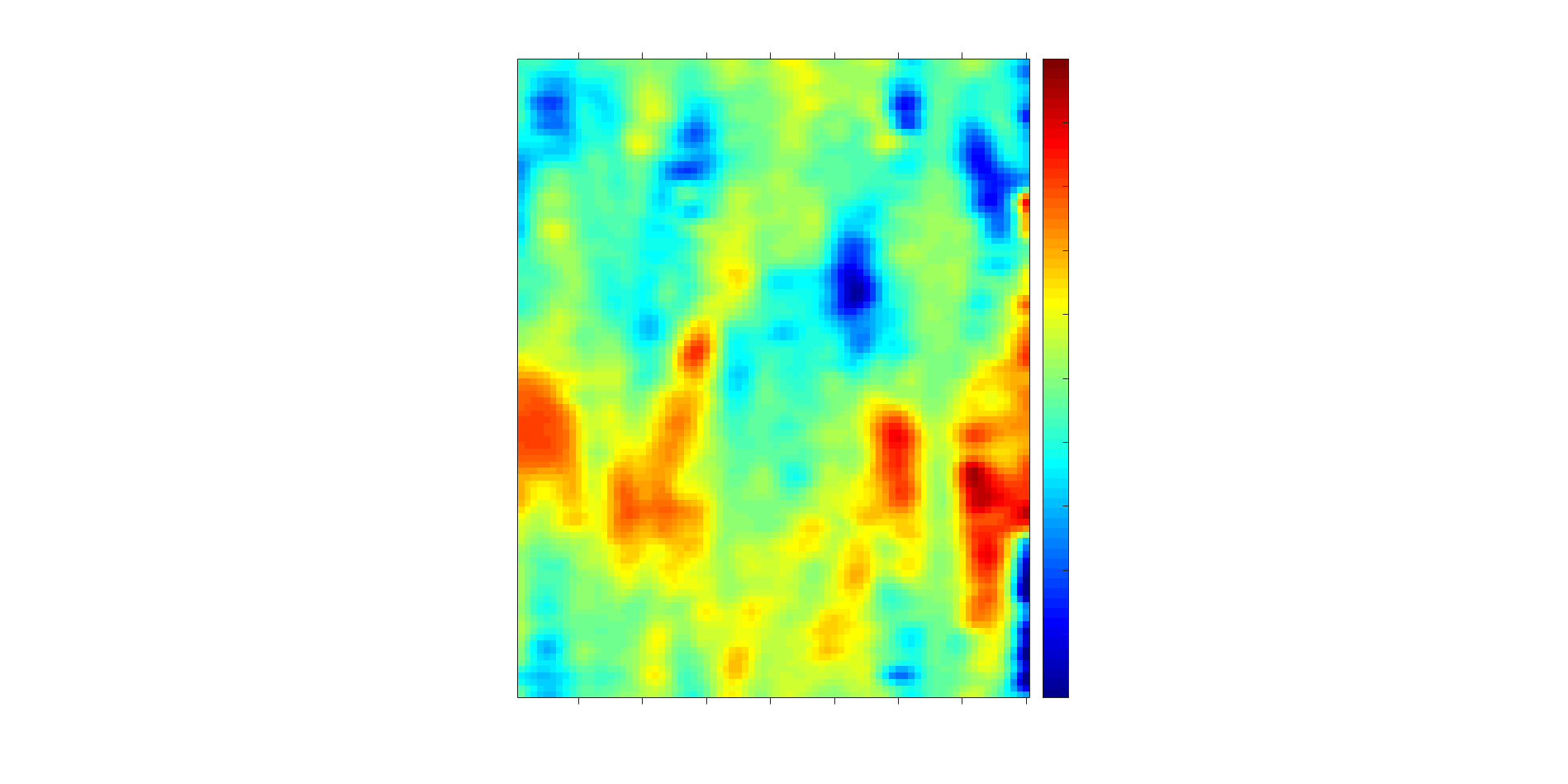

Supplement: Figure 3—source data 1. [file elife-69229-fig3-data1.zip › Figure3 Supp/control/c013_pos3_morpho_map_noshift_Xaxis_noLabel_25.png]

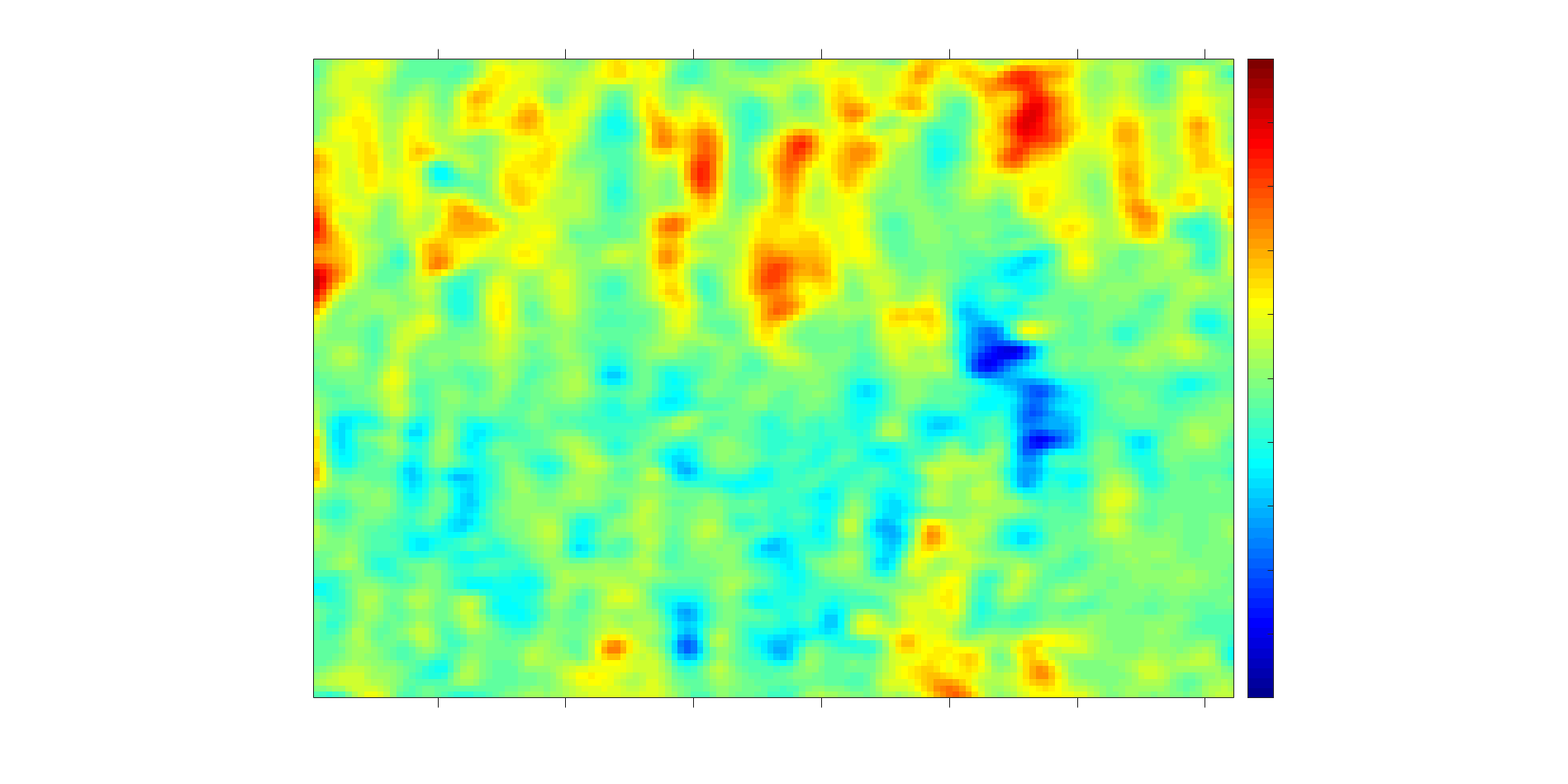

Supplement: Figure 3—source data 1. [file elife-69229-fig3-data1.zip › Figure3 Supp/control/c013_pos6_morpho_map_noshift_Xaxis_noLabel_25.png]

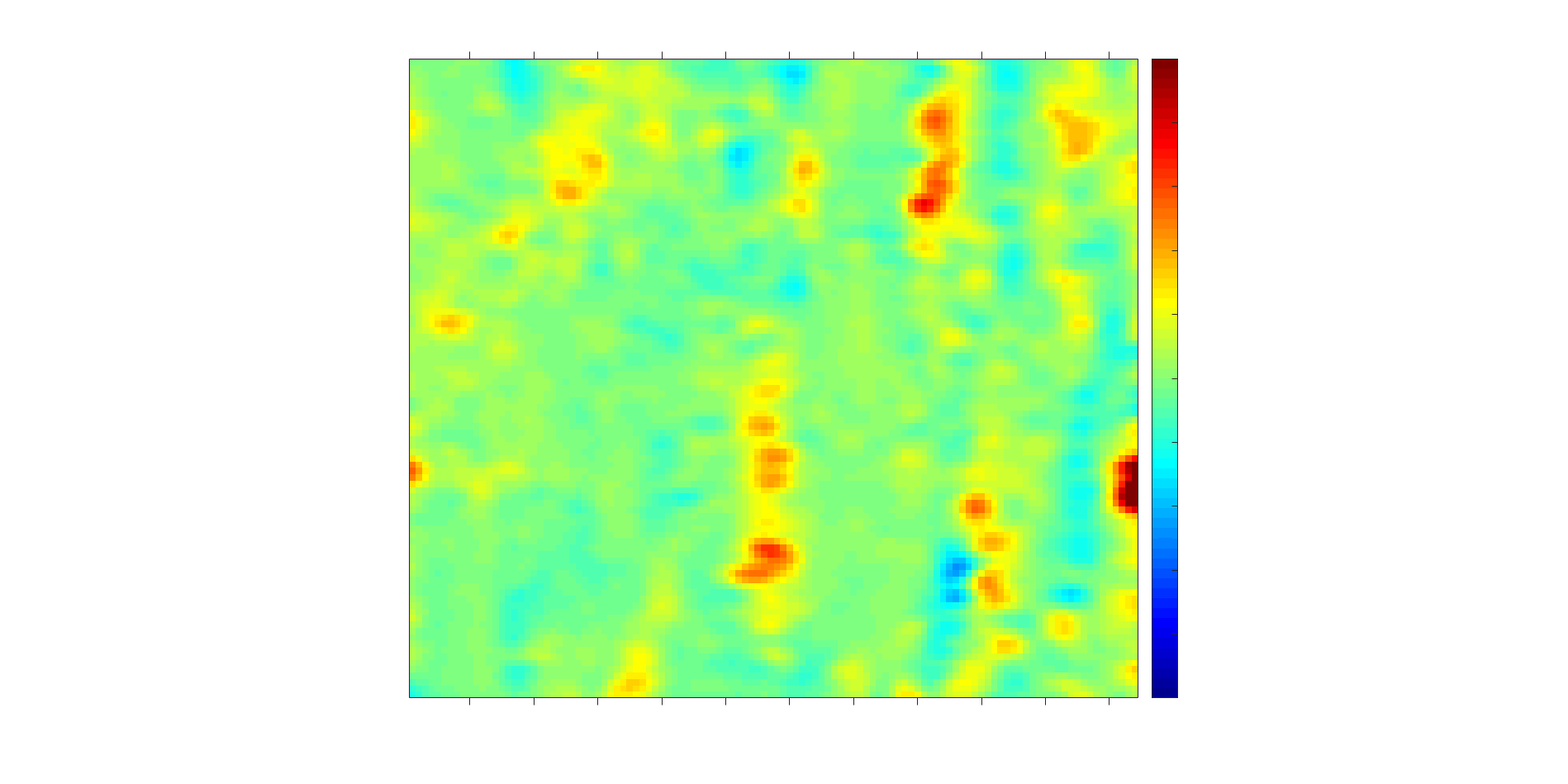

Supplement: Figure 3—source data 1. [file elife-69229-fig3-data1.zip › Figure3 Supp/control/c013_pos8_morpho_map_noshift_Xaxis_noLabel_25.png]

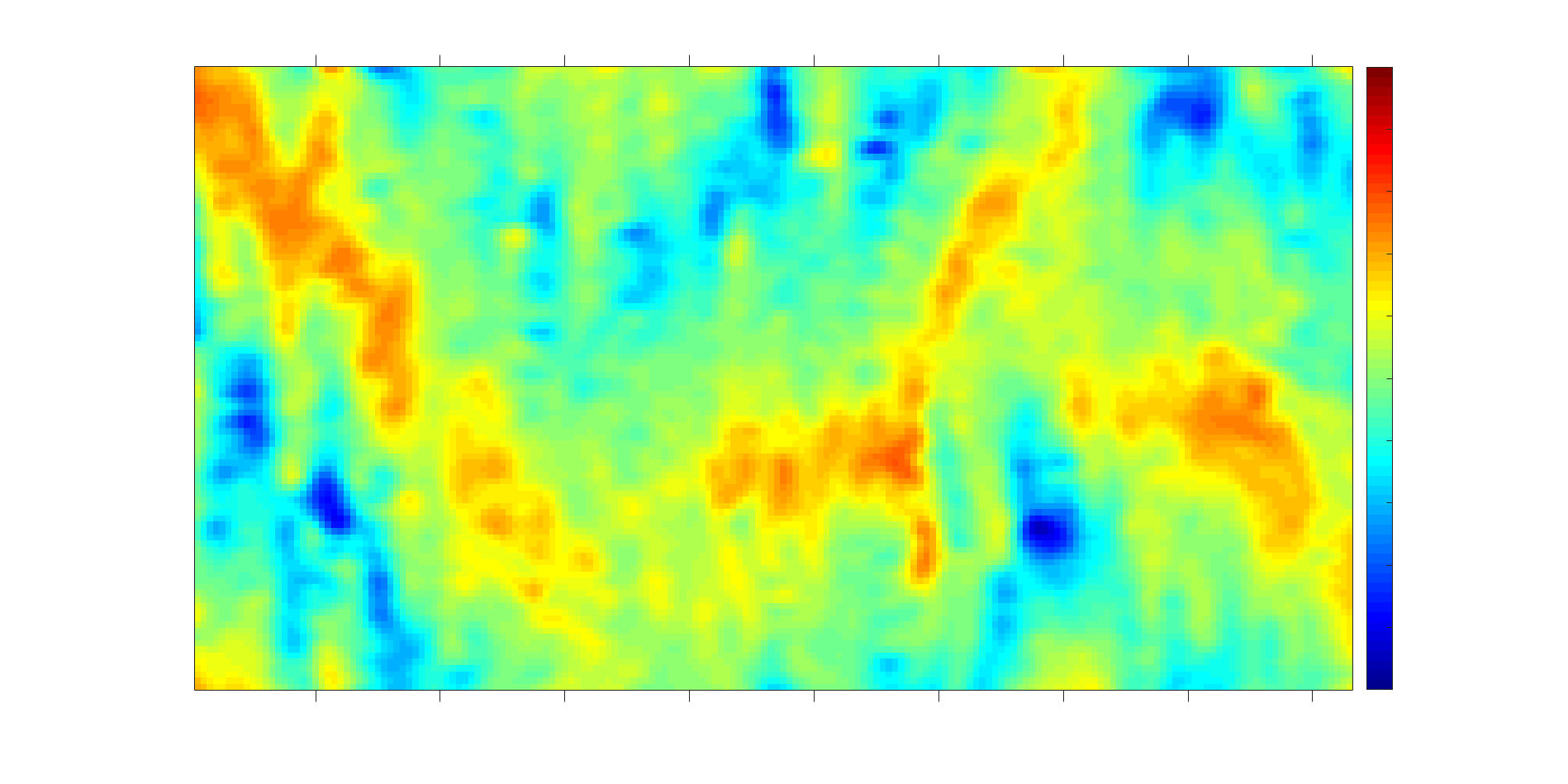

Supplement: Figure 3—source data 1. [file elife-69229-fig3-data1.zip › Figure3 Supp/control/c014_pos12_morpho_map_noshift_Xaxis_noLabel_25.png]

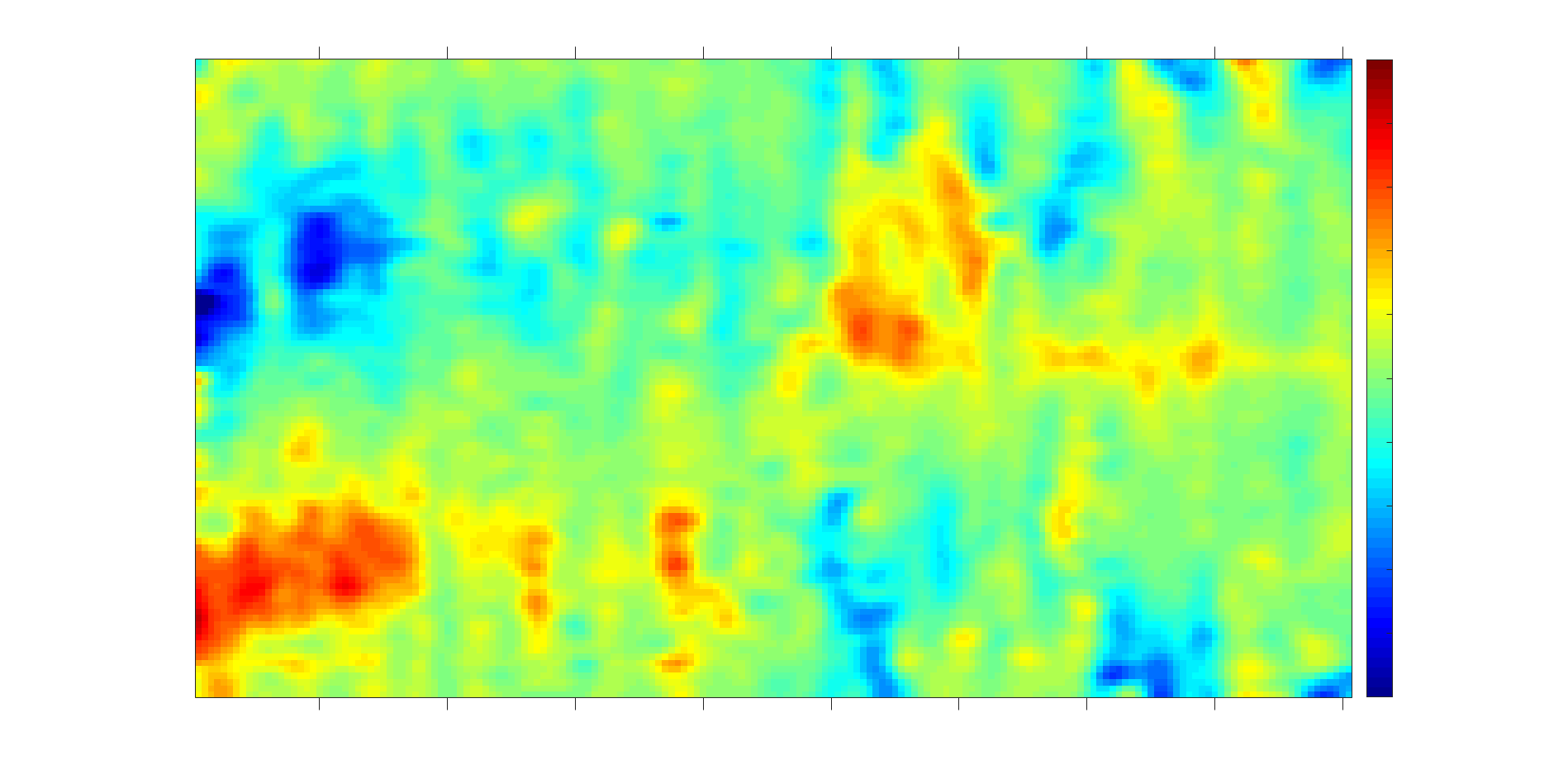

Supplement: Figure 3—source data 1. [file elife-69229-fig3-data1.zip › Figure3 Supp/control/c014_pos13_morpho_map_noshift_Xaxis_noLabel_25.png]

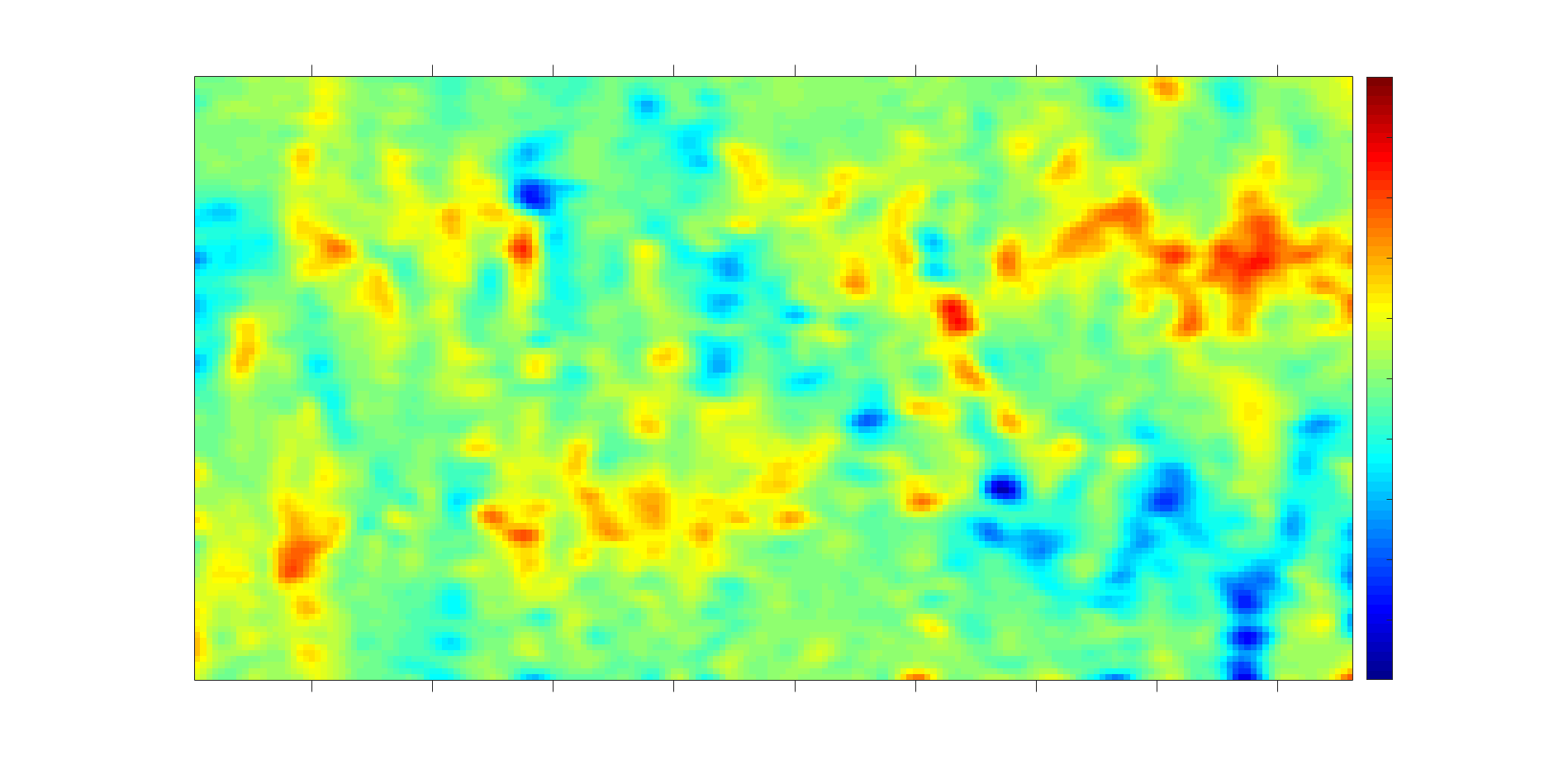

Supplement: Figure 3—source data 1. [file elife-69229-fig3-data1.zip › Figure3 Supp/control/c014_pos14_morpho_map_noshift_Xaxis_noLabel_25.png]

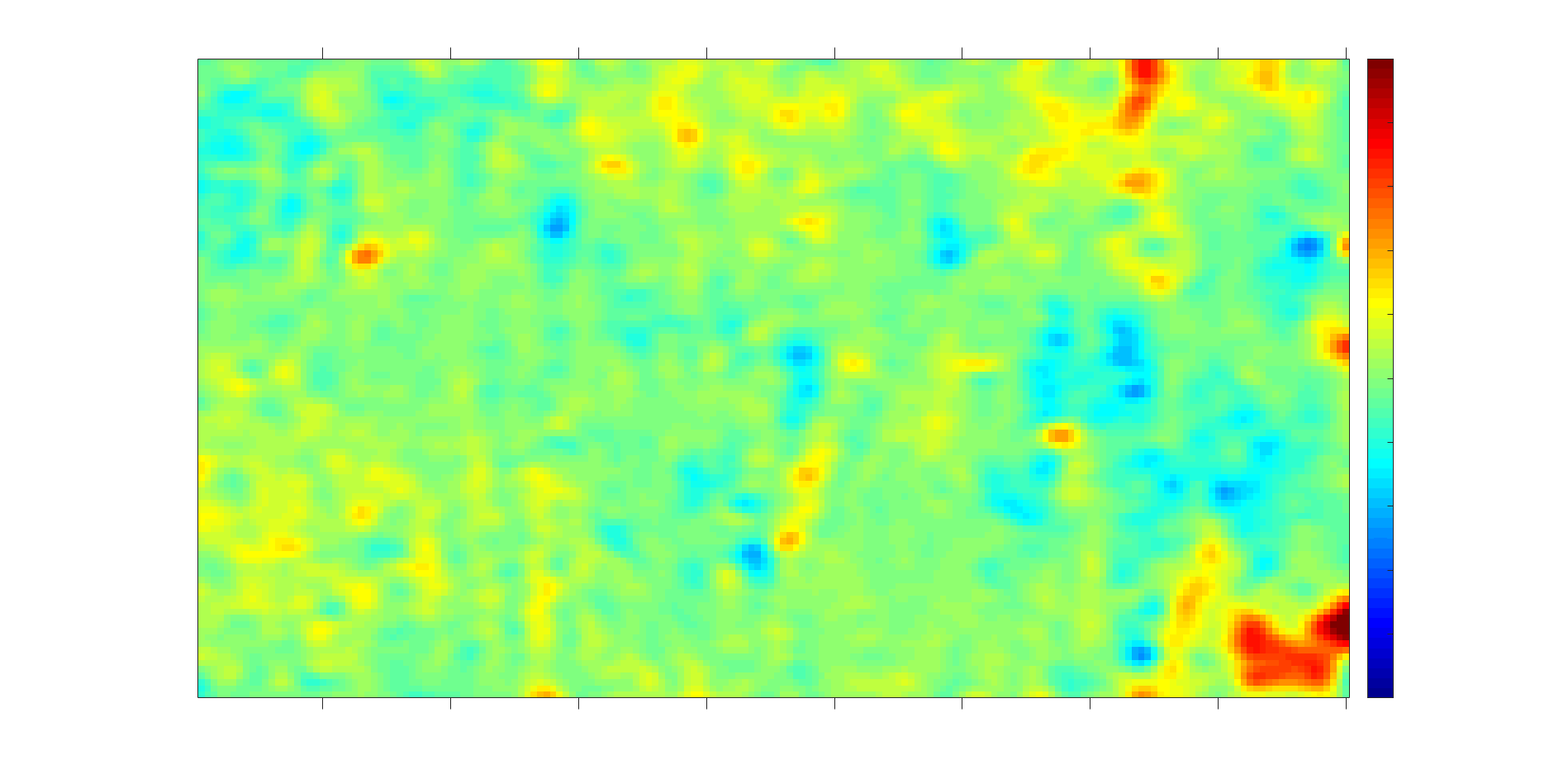

Supplement: Figure 3—source data 1. [file elife-69229-fig3-data1.zip › Figure3 Supp/control/c014_pos1_morpho_map_noshift_Xaxis_noLabel_25.png]

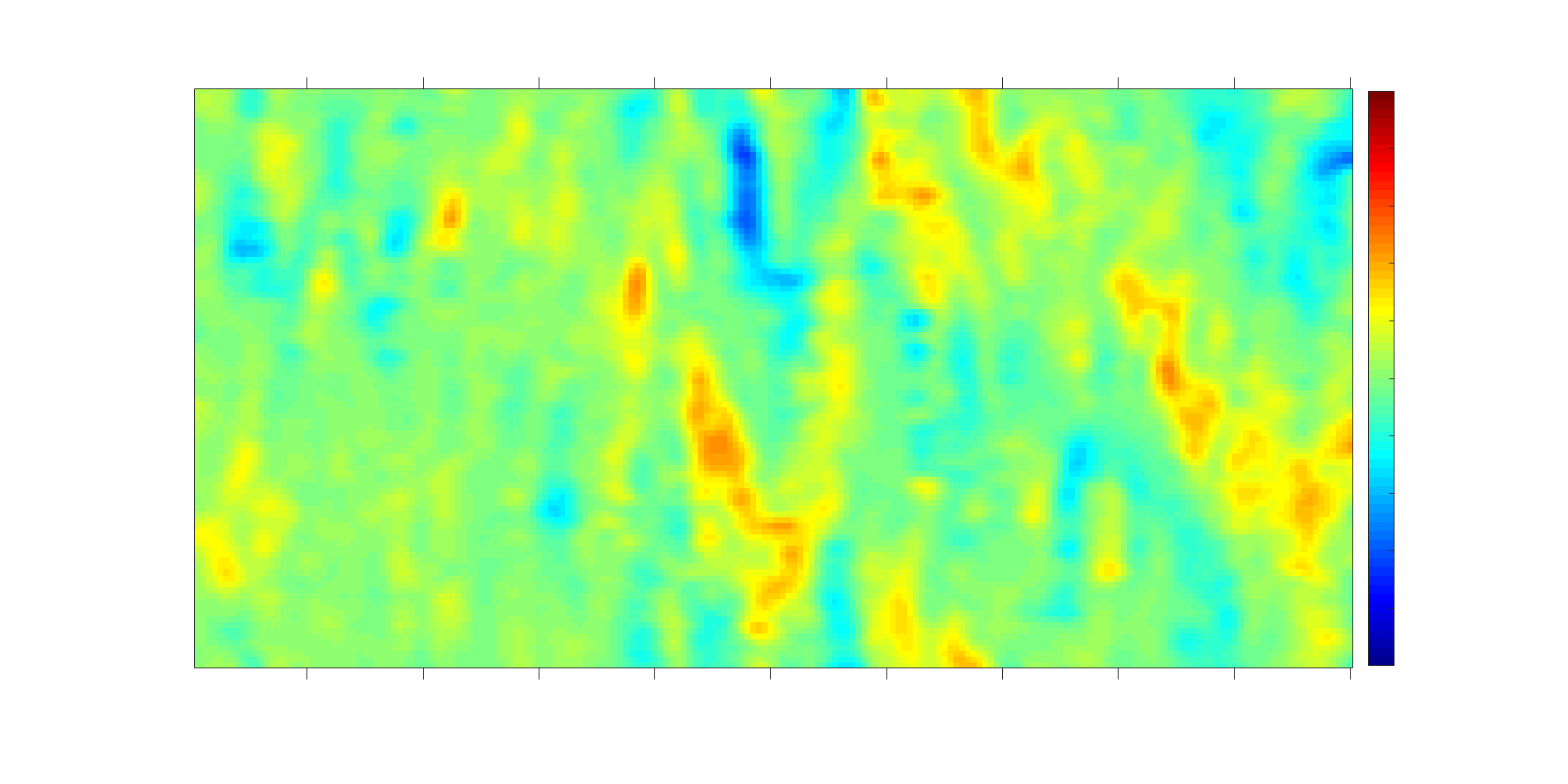

Supplement: Figure 3—source data 1. [file elife-69229-fig3-data1.zip › Figure3 Supp/control/c014_pos2_morpho_map_noshift_Xaxis_noLabel_25.png]

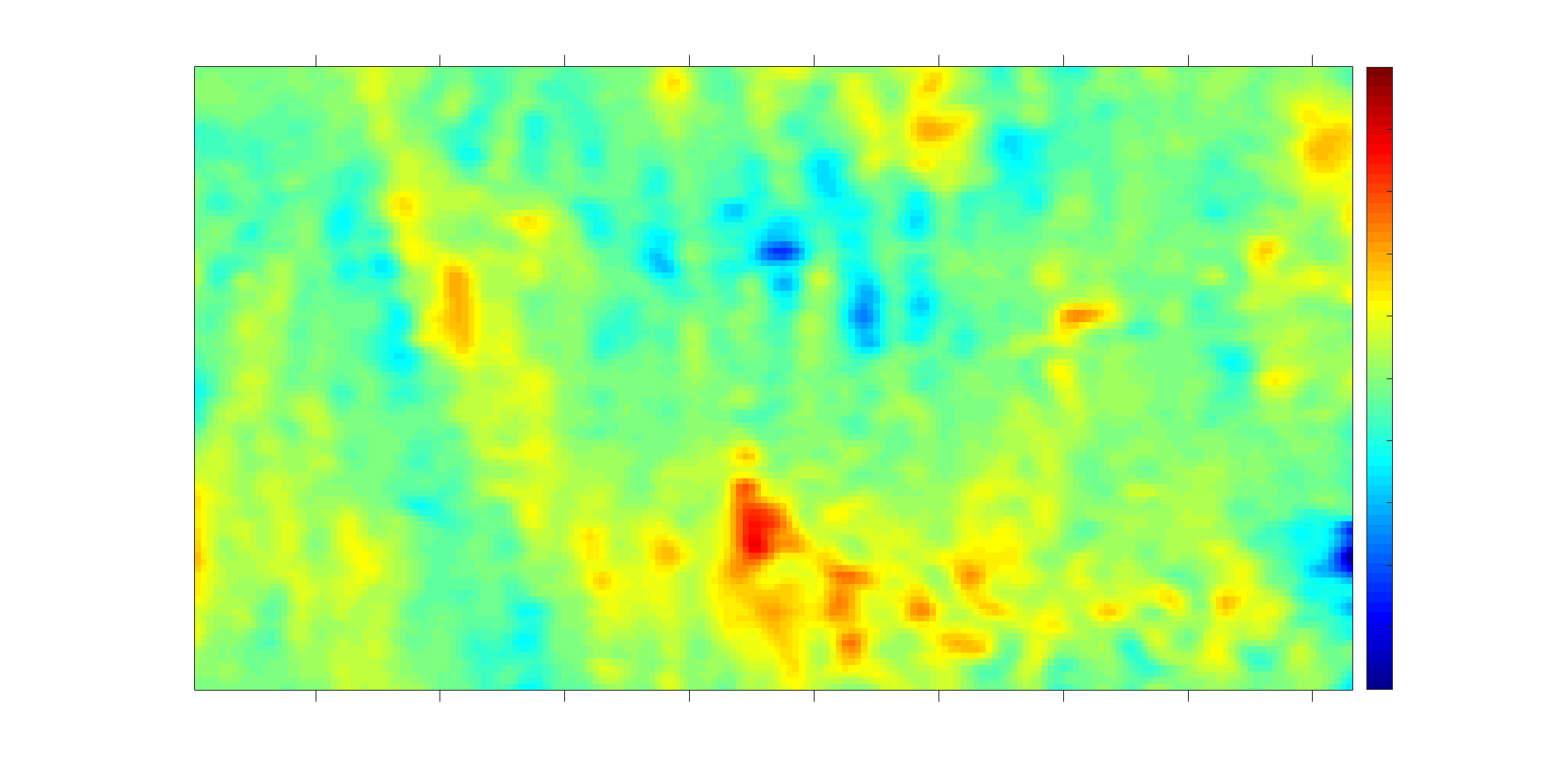

Supplement: Figure 3—source data 1. [file elife-69229-fig3-data1.zip › Figure3 Supp/control/c014_pos6_morpho_map_noshift_Xaxis_noLabel_25.png]

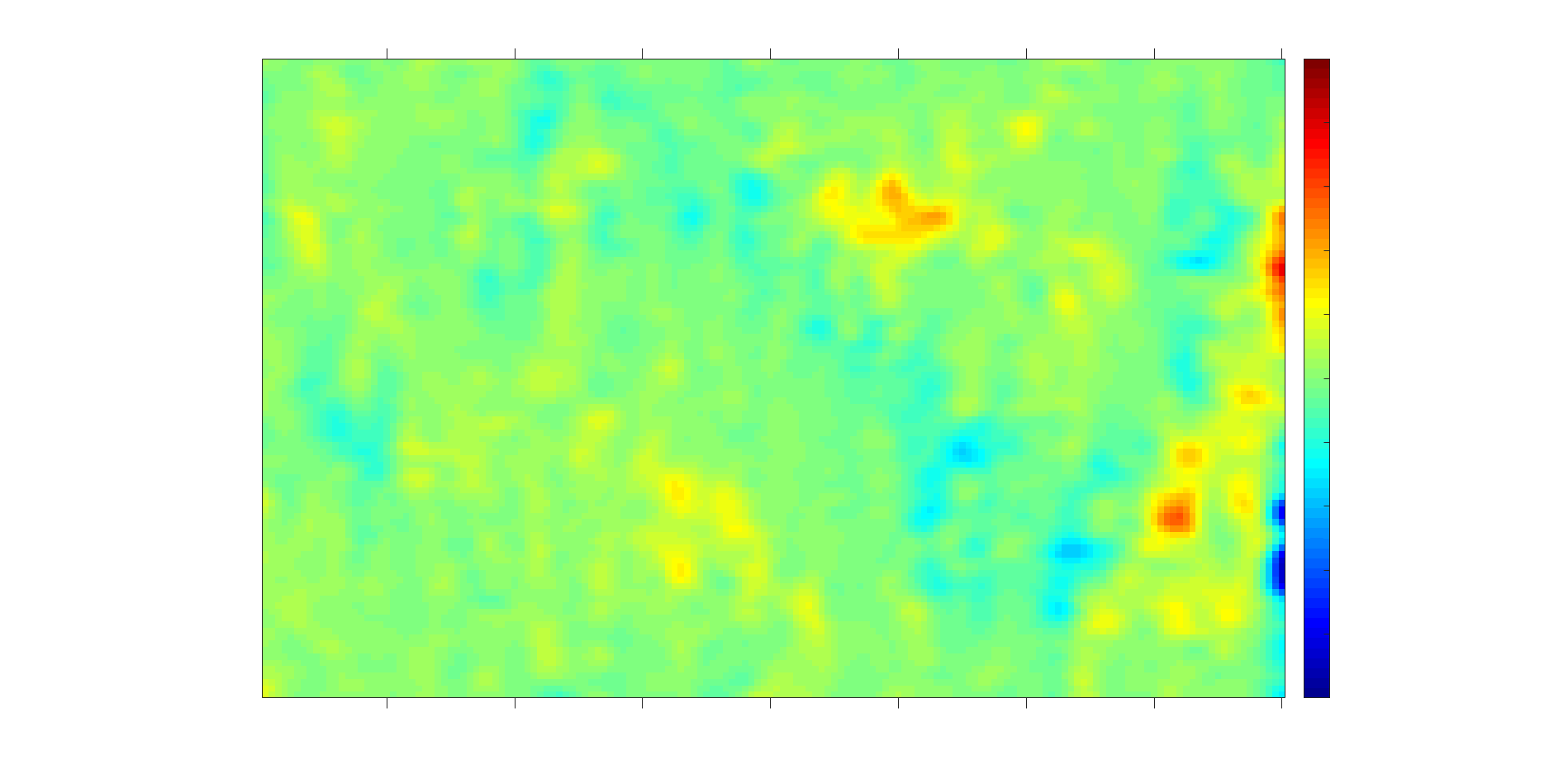

Supplement: Figure 3—source data 1. [file elife-69229-fig3-data1.zip › Figure3 Supp/control/c014_pos7_morpho_map_noshift_Xaxis_noLabel_25.png]

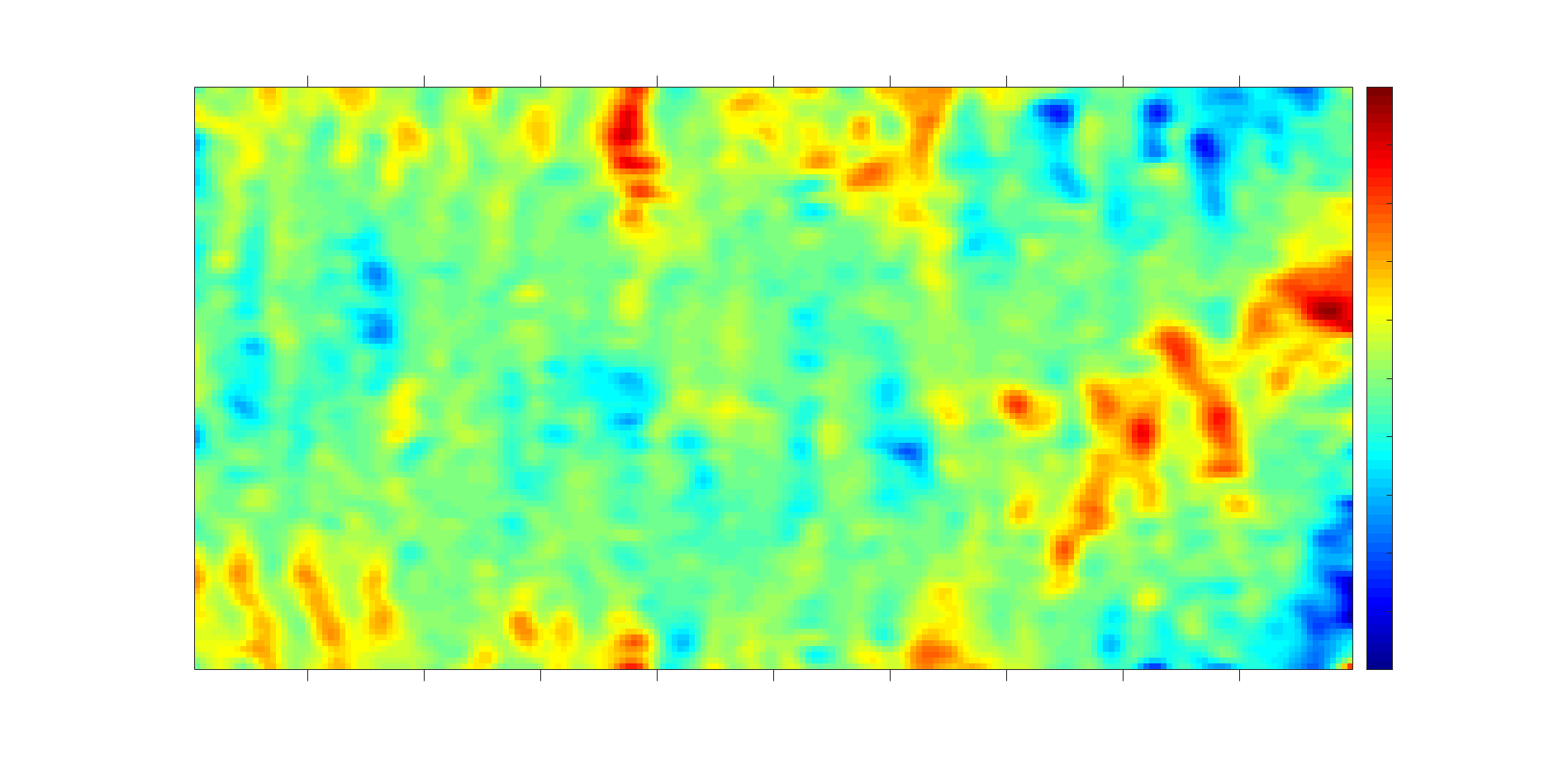

Supplement: Figure 3—source data 1. [file elife-69229-fig3-data1.zip › Figure3 Supp/control/c015_pos5_morpho_map_noshift_Xaxis_noLabel_25.png]

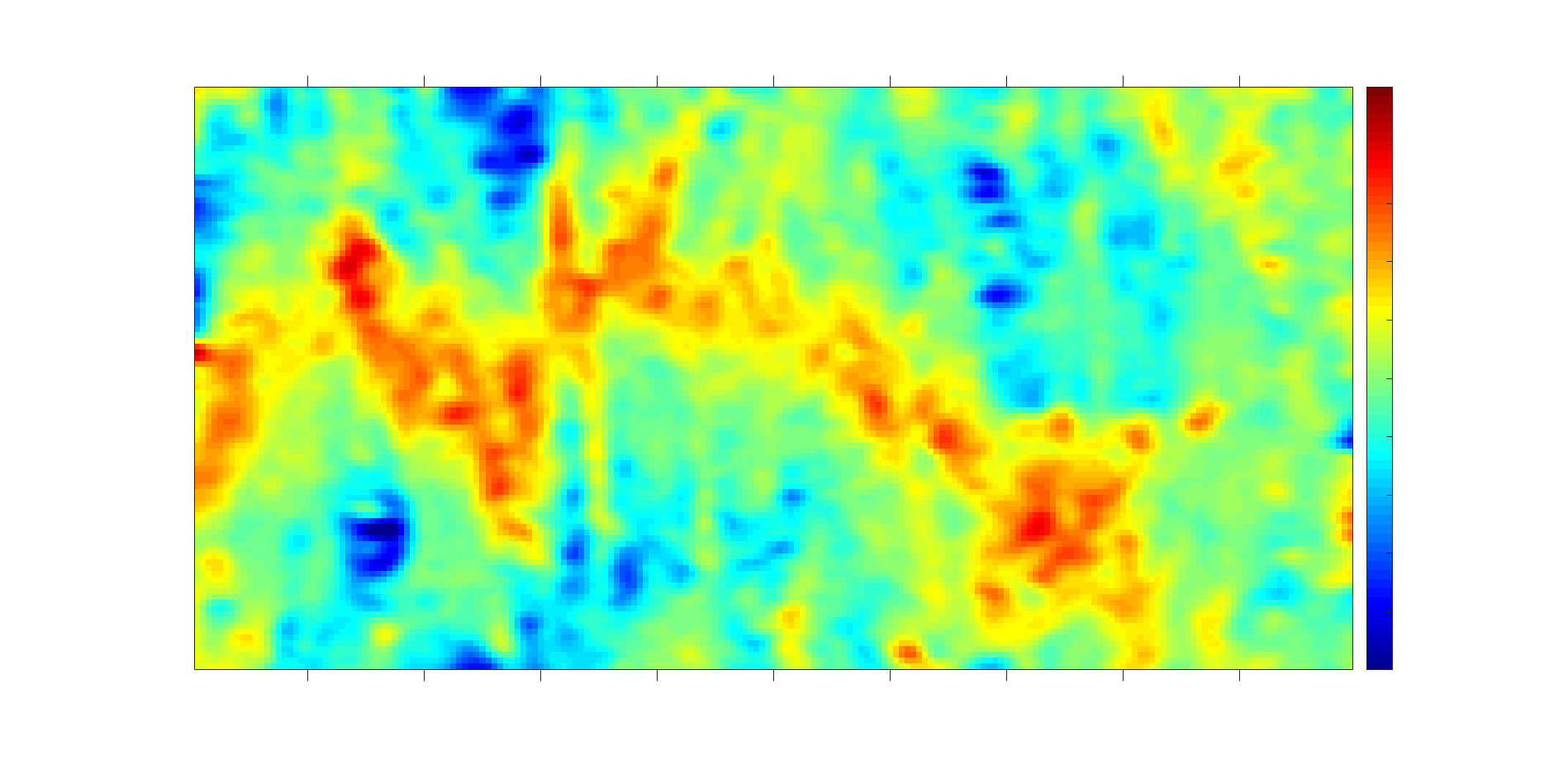

Supplement: Figure 3—source data 1. [file elife-69229-fig3-data1.zip › Figure3 Supp/control/c015_pos6_morpho_map_noshift_Xaxis_noLabel_25.png]

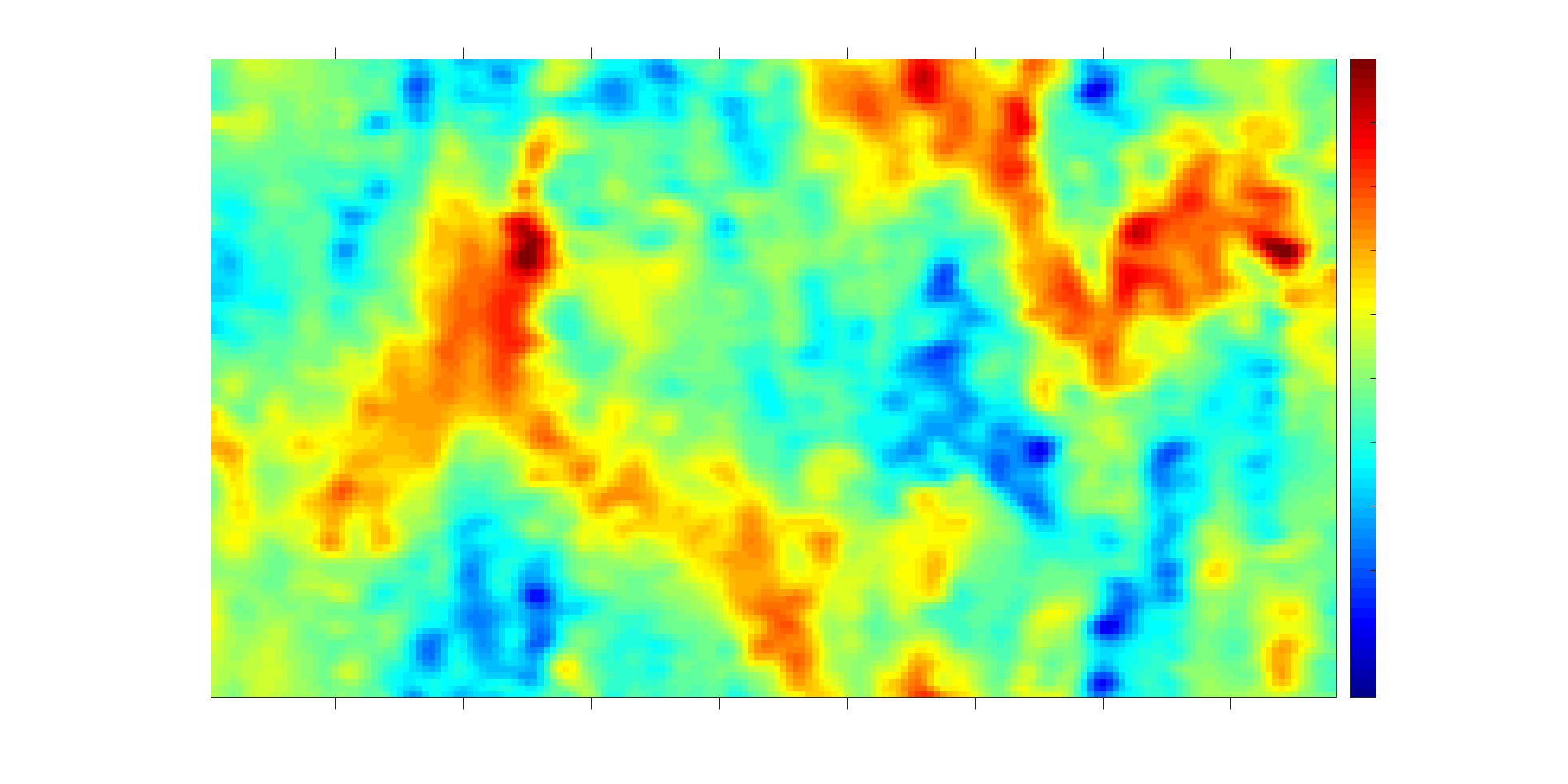

Supplement: Figure 3—source data 1. [file elife-69229-fig3-data1.zip › Figure3 Supp/control/c015_pos7_morpho_map_noshift_Xaxis_noLabel_25.png]

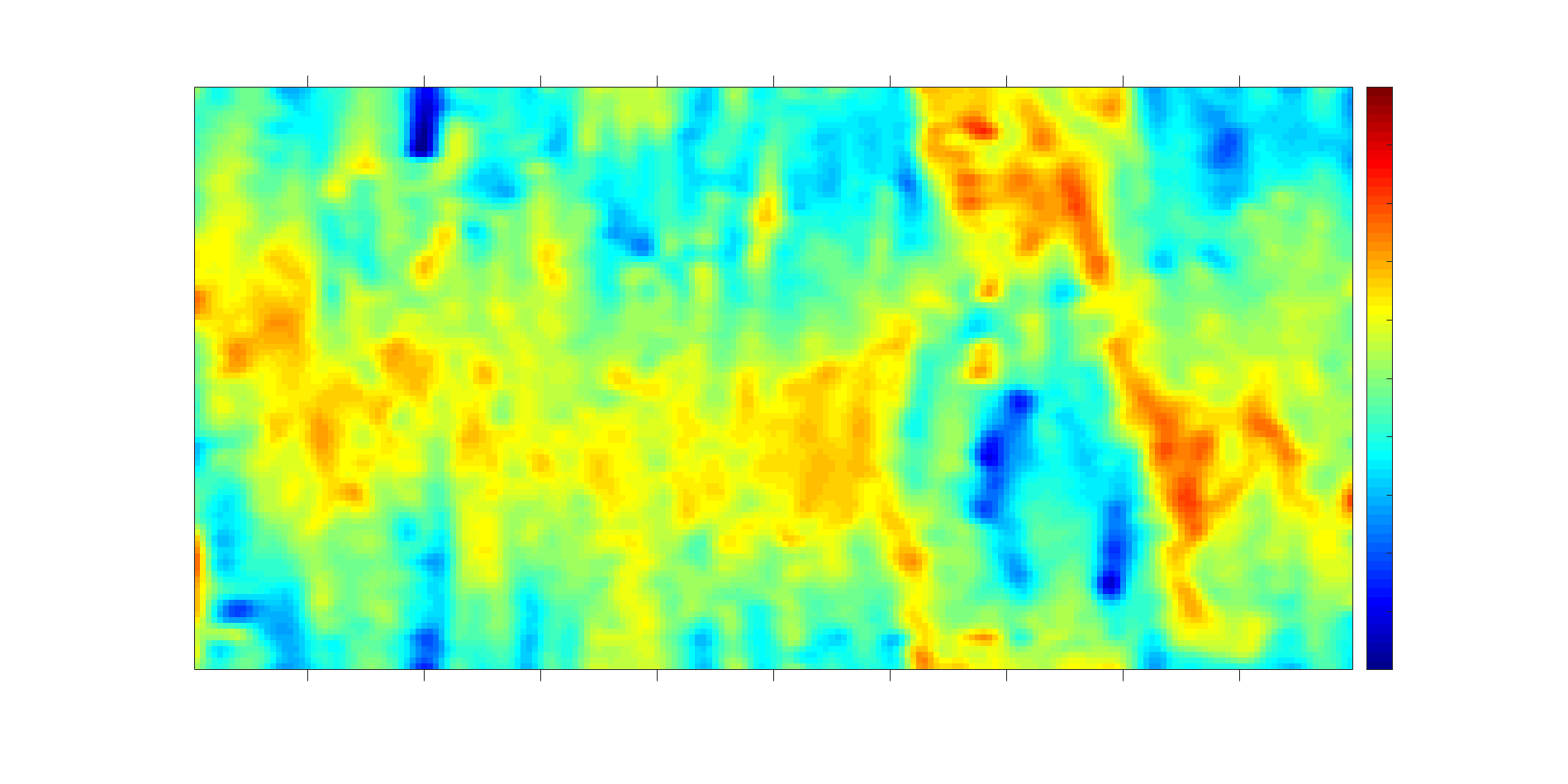

Supplement: Figure 3—source data 1. [file elife-69229-fig3-data1.zip › Figure3 Supp/control/c015_pos8_morpho_map_noshift_Xaxis_noLabel_25.png]

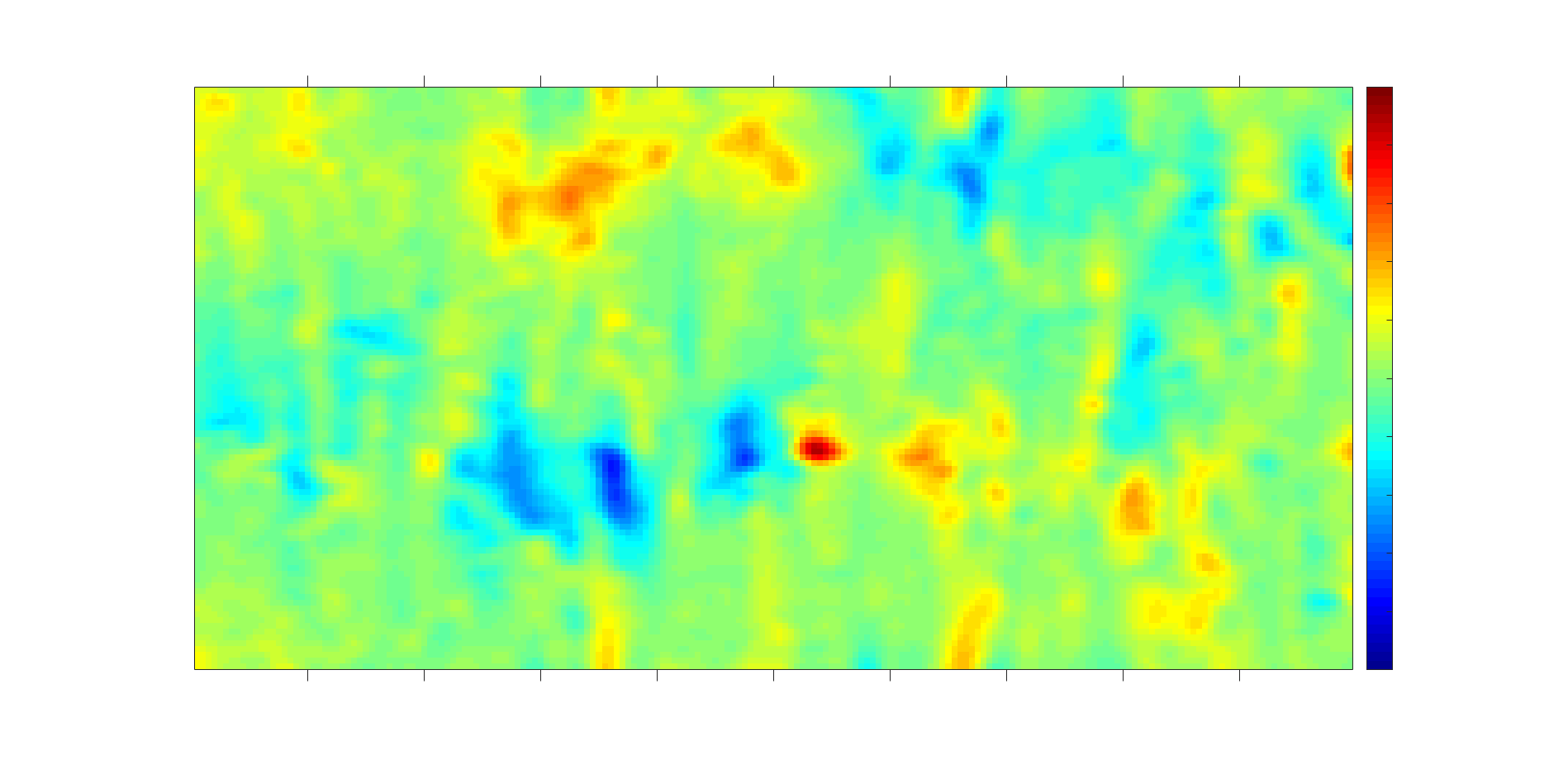

Supplement: Figure 3—source data 1. [file elife-69229-fig3-data1.zip › Figure3 Supp/control/c015_pos9_morpho_map_noshift_Xaxis_noLabel_25.png]

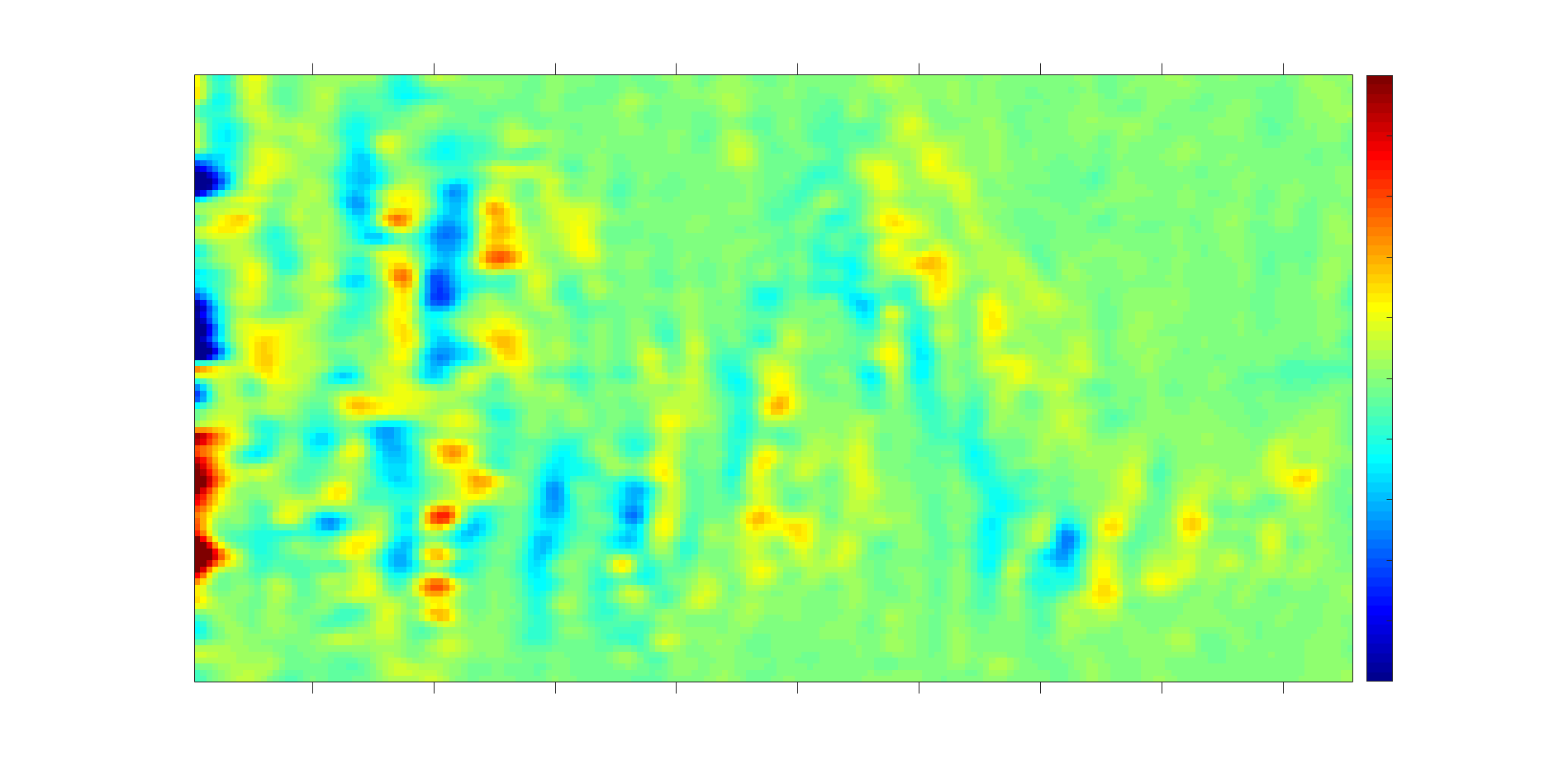

Supplement: Figure 3—source data 1. [file elife-69229-fig3-data1.zip › Figure3 Supp/Nocodazole/c0020_pos11__RawMorpho_noLabel_25.png]

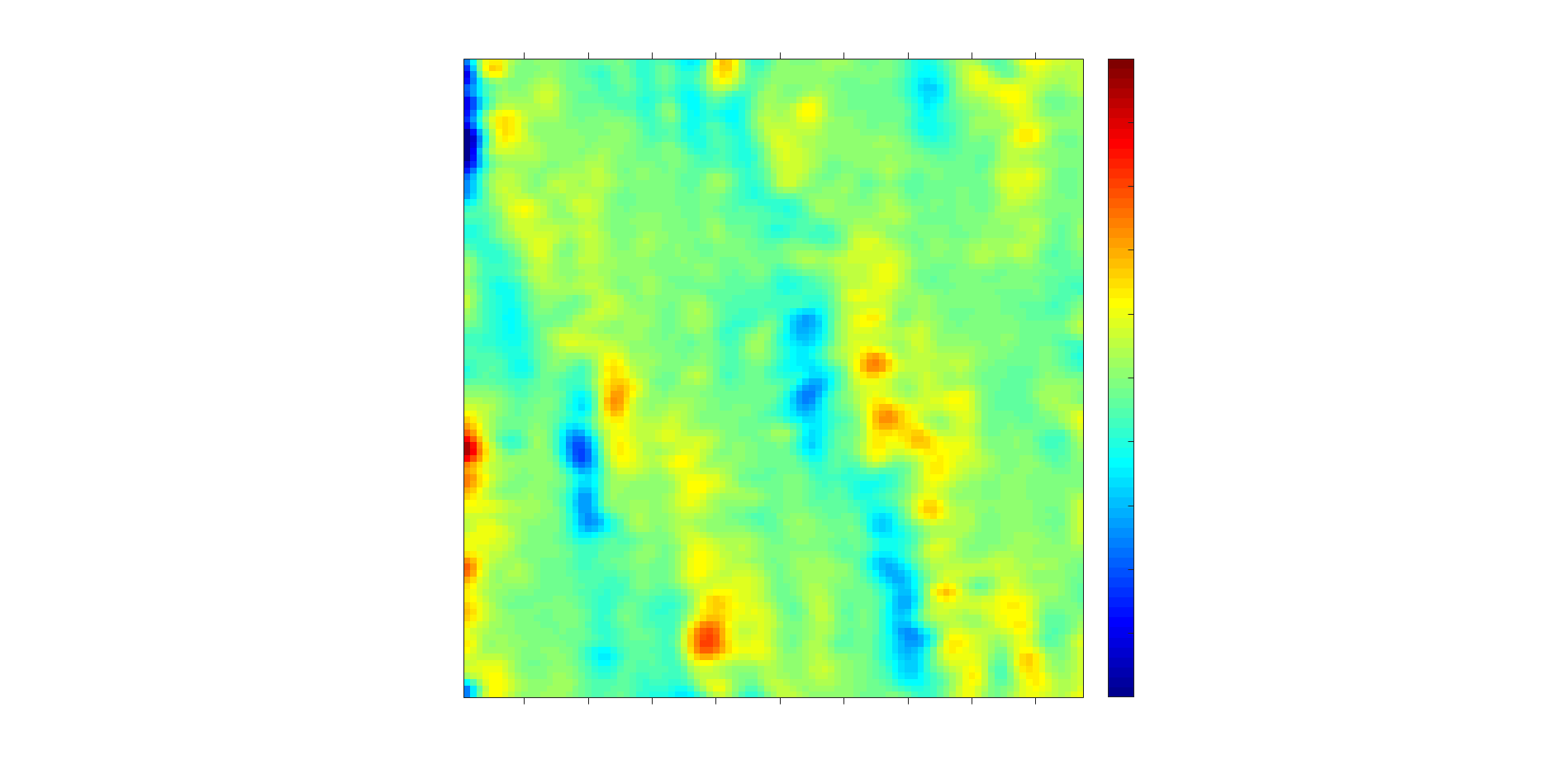

Supplement: Figure 3—source data 1. [file elife-69229-fig3-data1.zip › Figure3 Supp/Nocodazole/c0020_pos12__RawMorpho_noLabel_25.png]

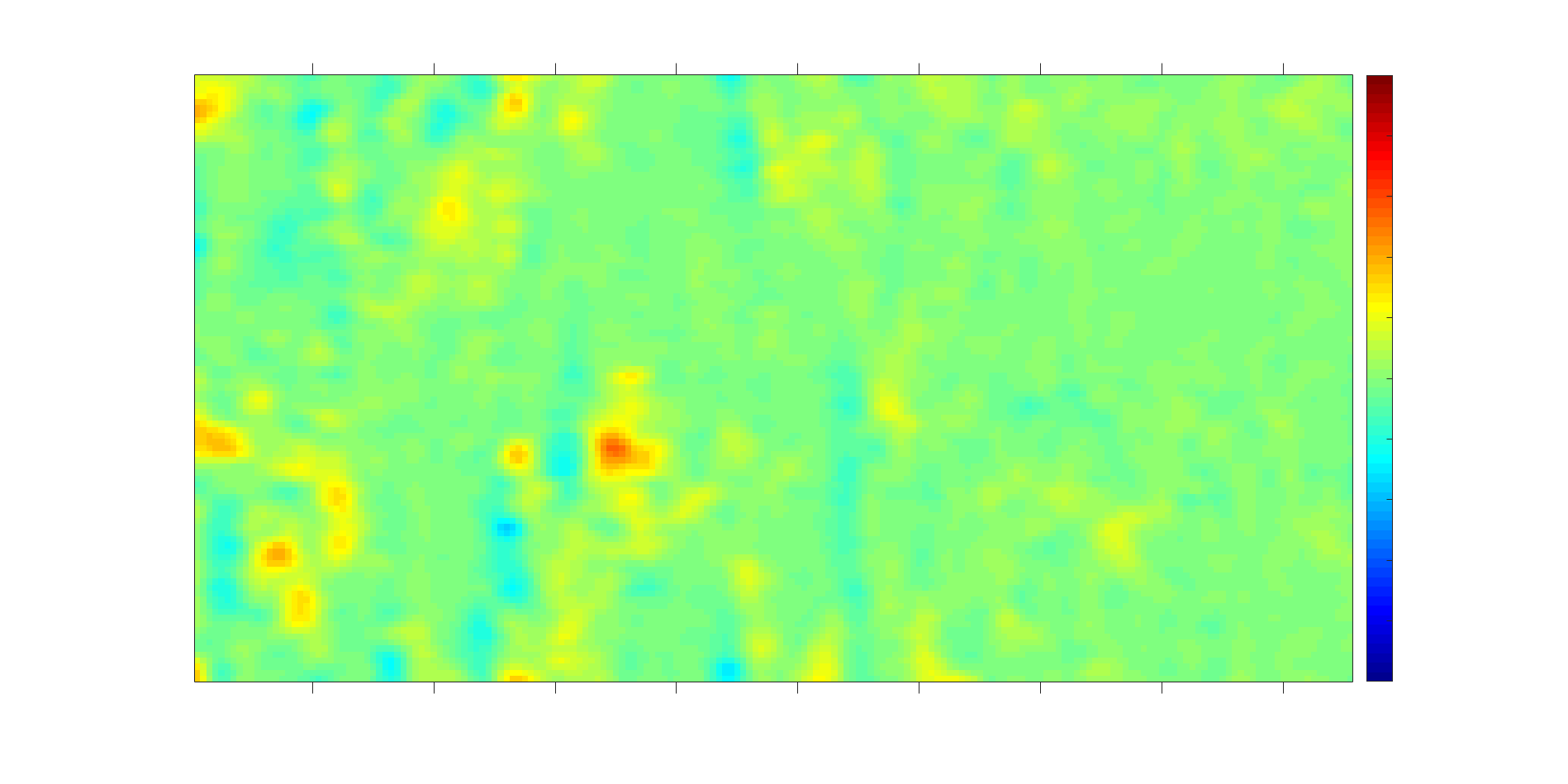

Supplement: Figure 3—source data 1. [file elife-69229-fig3-data1.zip › Figure3 Supp/Nocodazole/c0020_pos13__RawMorpho_noLabel_25.png]

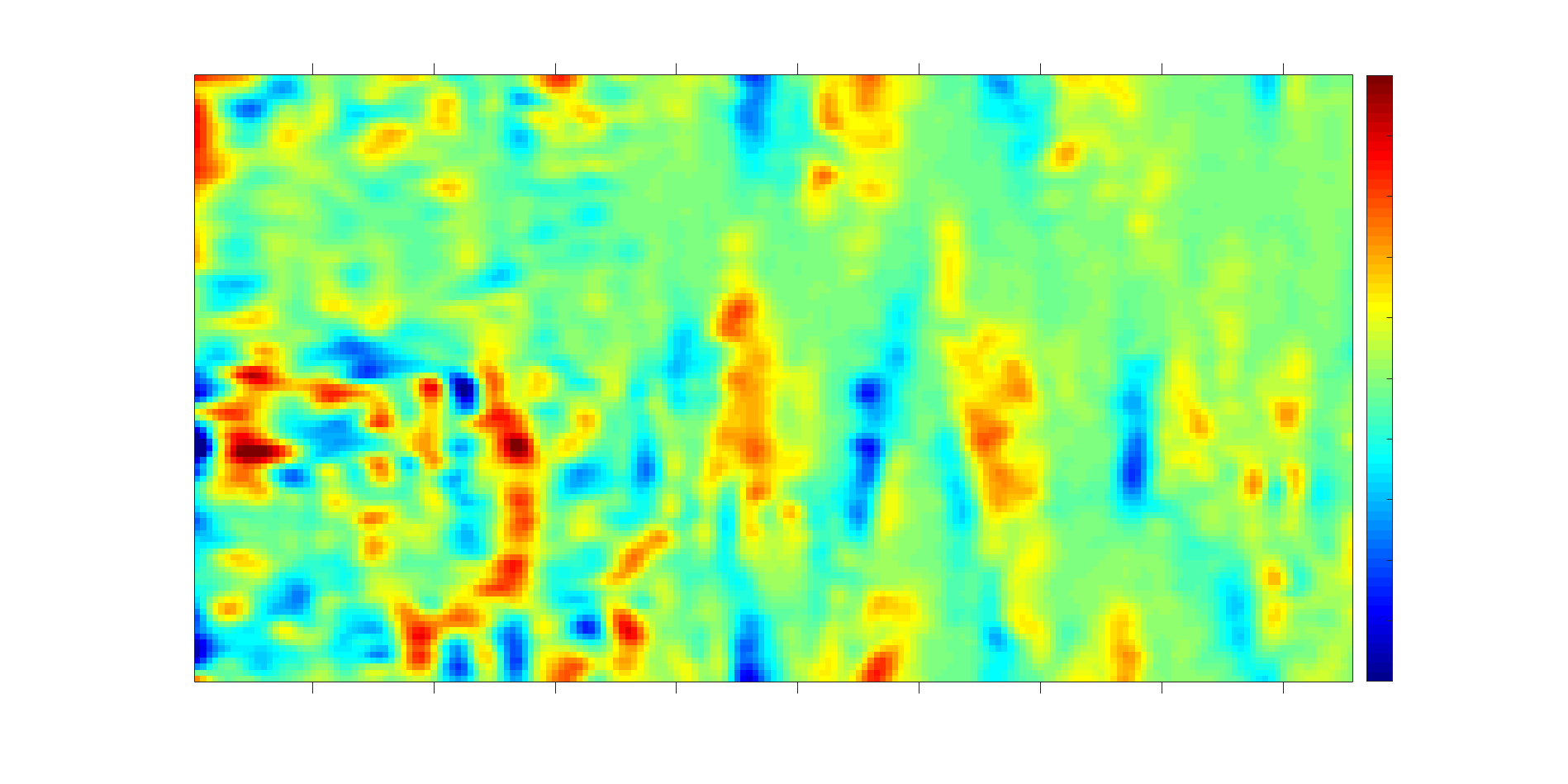

Supplement: Figure 3—source data 1. [file elife-69229-fig3-data1.zip › Figure3 Supp/Nocodazole/c0020_pos14__RawMorpho_noLabel_25.png]

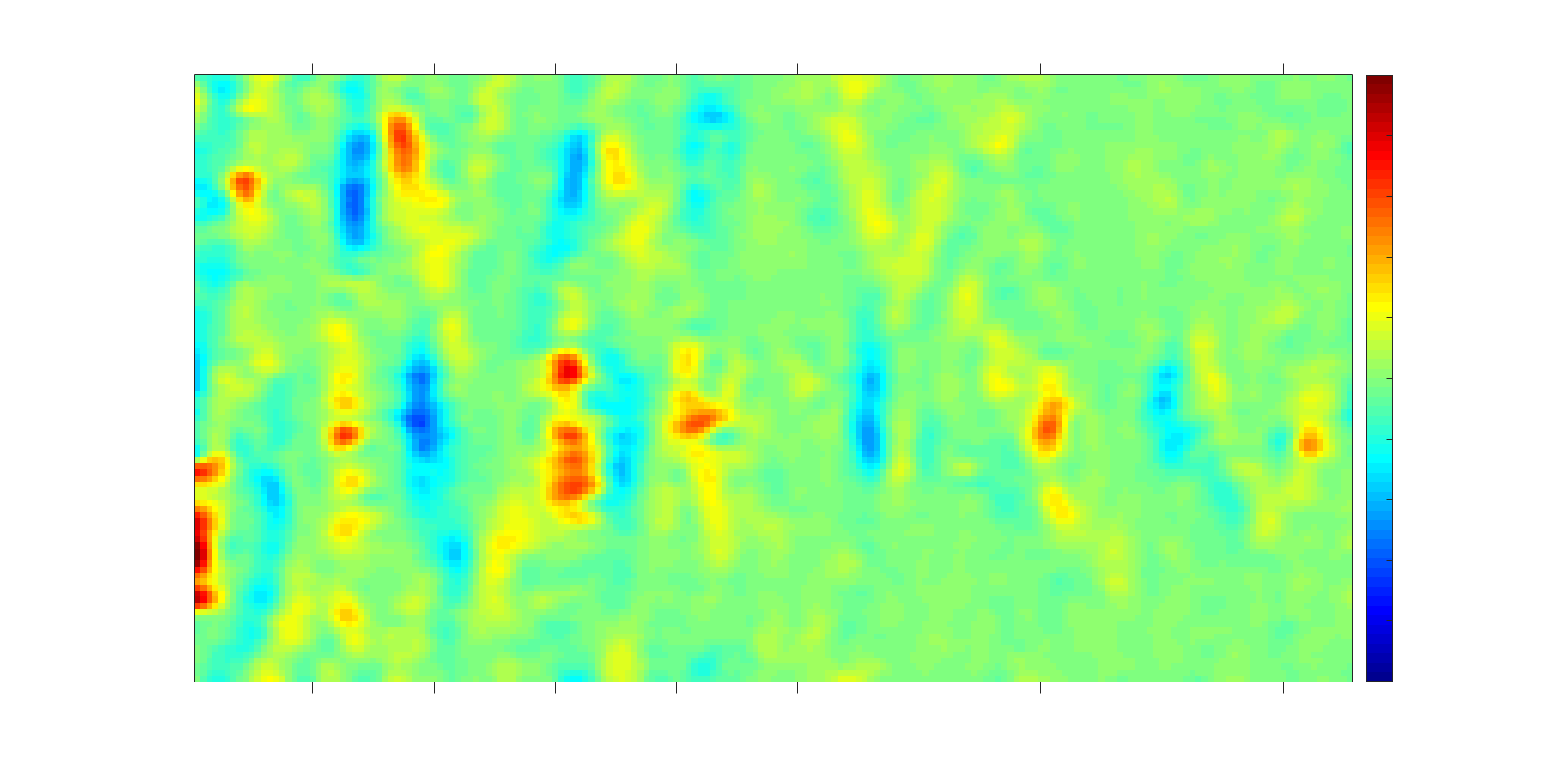

Supplement: Figure 3—source data 1. [file elife-69229-fig3-data1.zip › Figure3 Supp/Nocodazole/c0020_pos18__RawMorpho_noLabel_25.png]

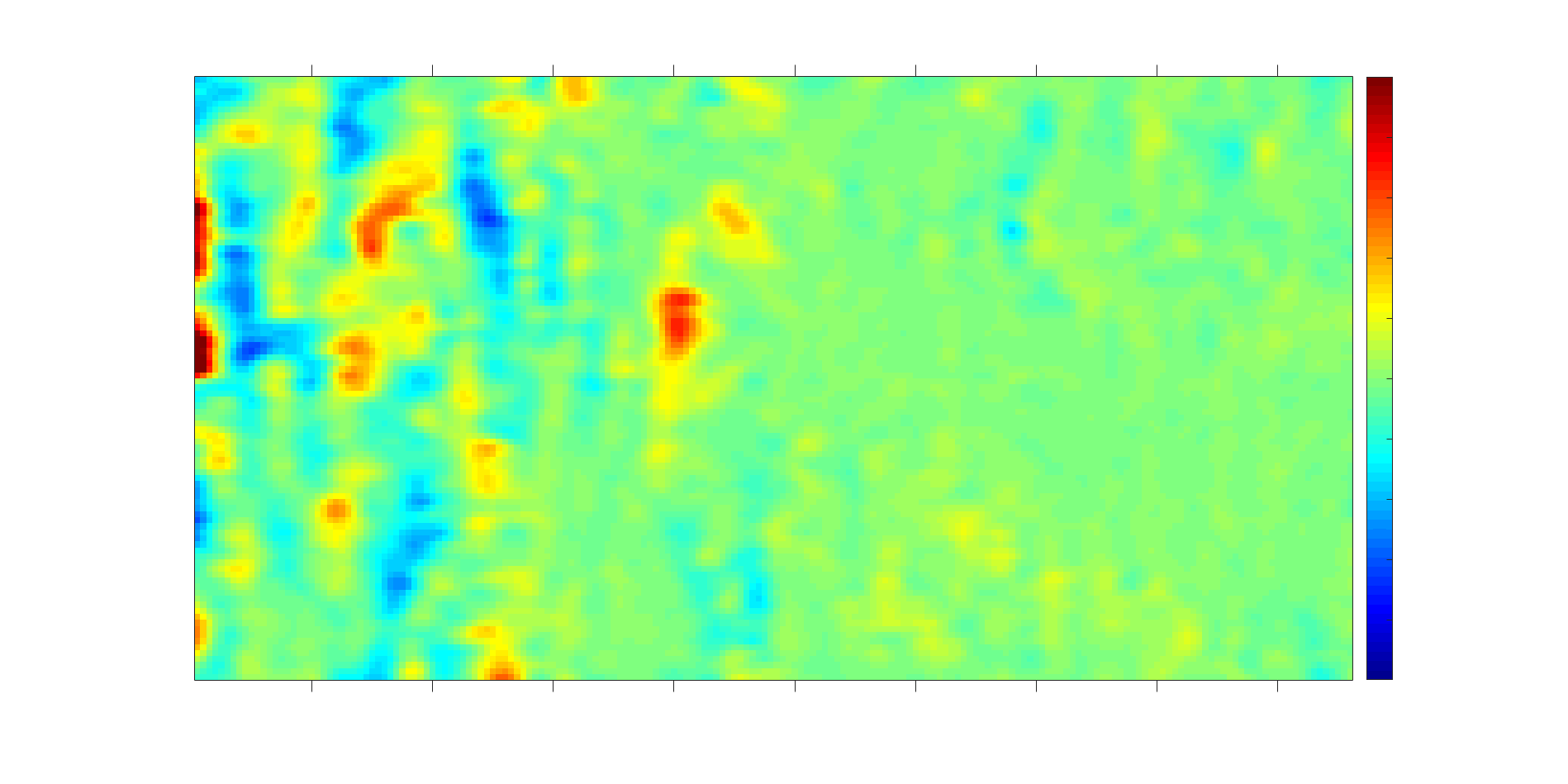

Supplement: Figure 3—source data 1. [file elife-69229-fig3-data1.zip › Figure3 Supp/Nocodazole/c0020_pos1__RawMorpho_noLabel_25.png]

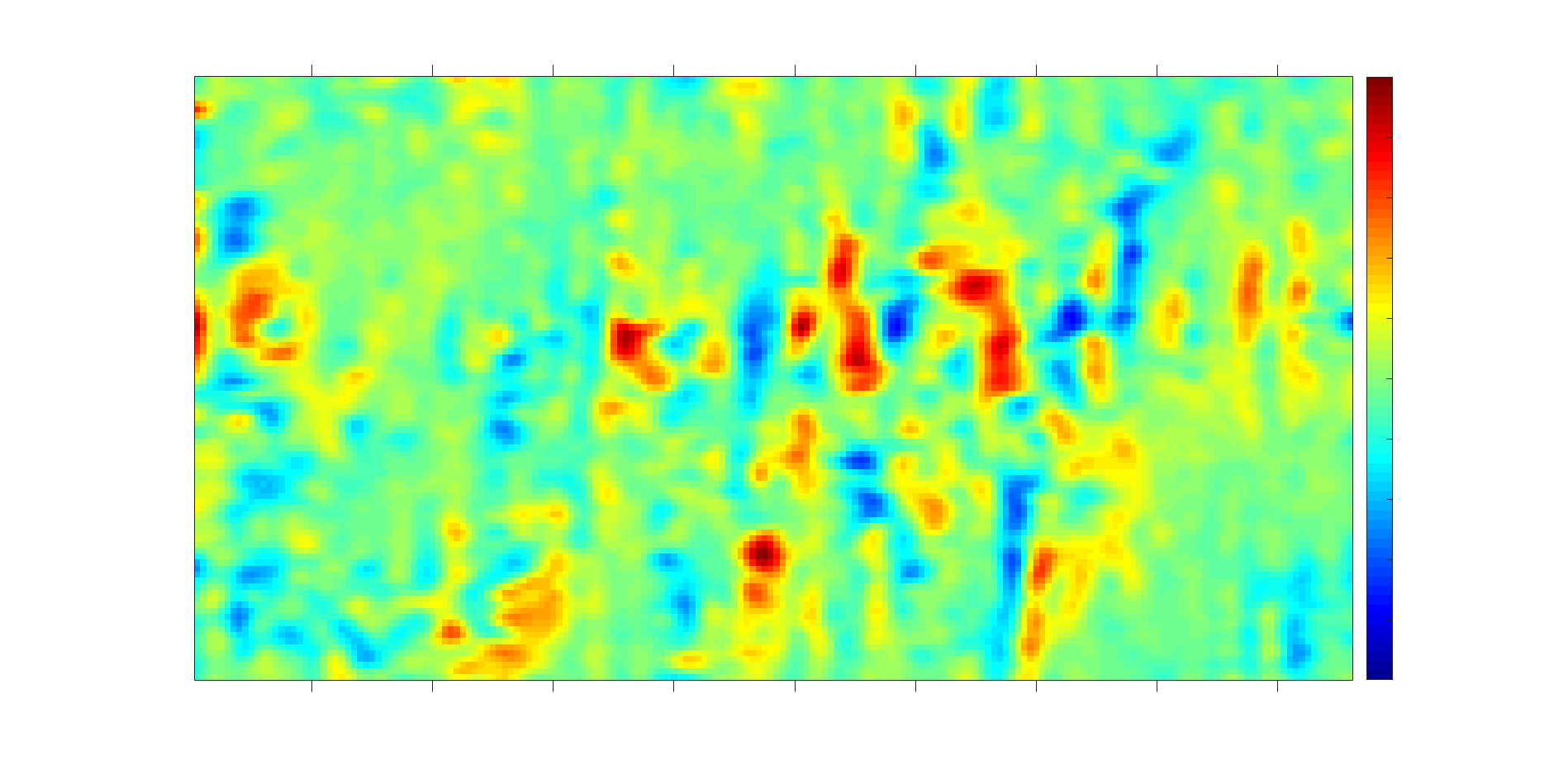

Supplement: Figure 3—source data 1. [file elife-69229-fig3-data1.zip › Figure3 Supp/Nocodazole/c0020_pos2__RawMorpho_noLabel_25.png]

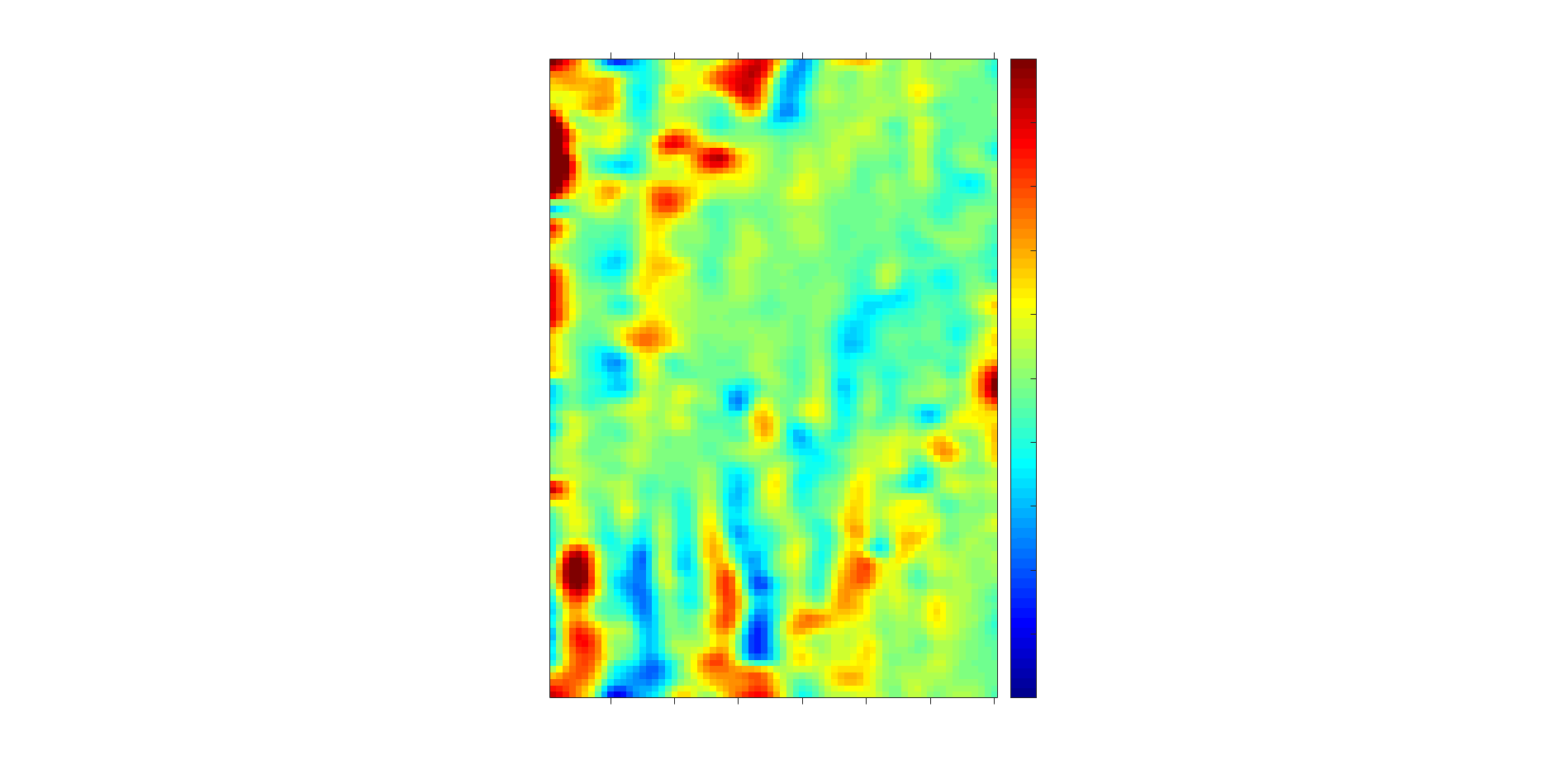

Supplement: Figure 3—source data 1. [file elife-69229-fig3-data1.zip › Figure3 Supp/Nocodazole/c0020_pos3__RawMorpho_noLabel_25.png]

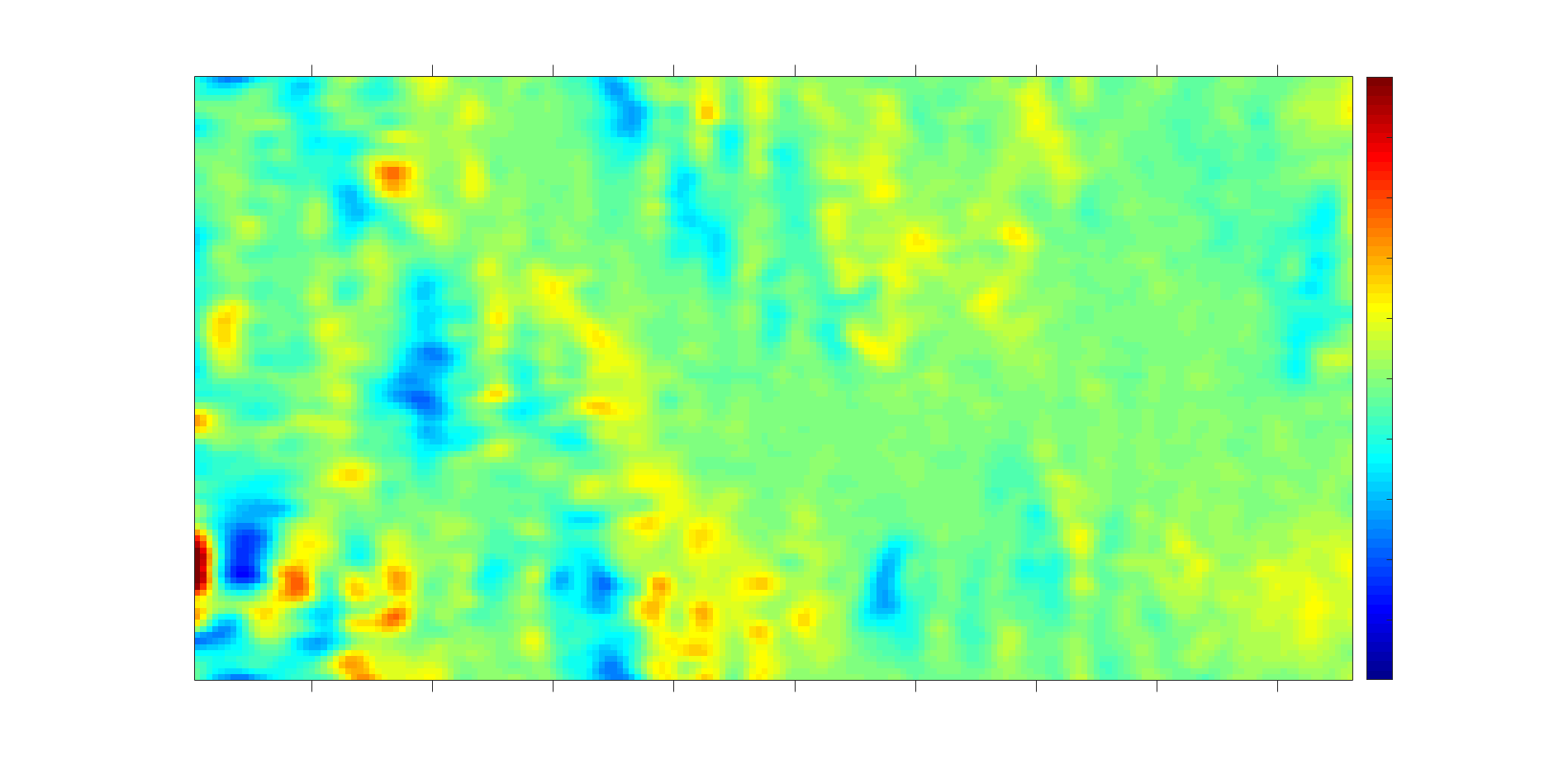

Supplement: Figure 3—source data 1. [file elife-69229-fig3-data1.zip › Figure3 Supp/Nocodazole/c0020_pos4__RawMorpho_noLabel_25.png]

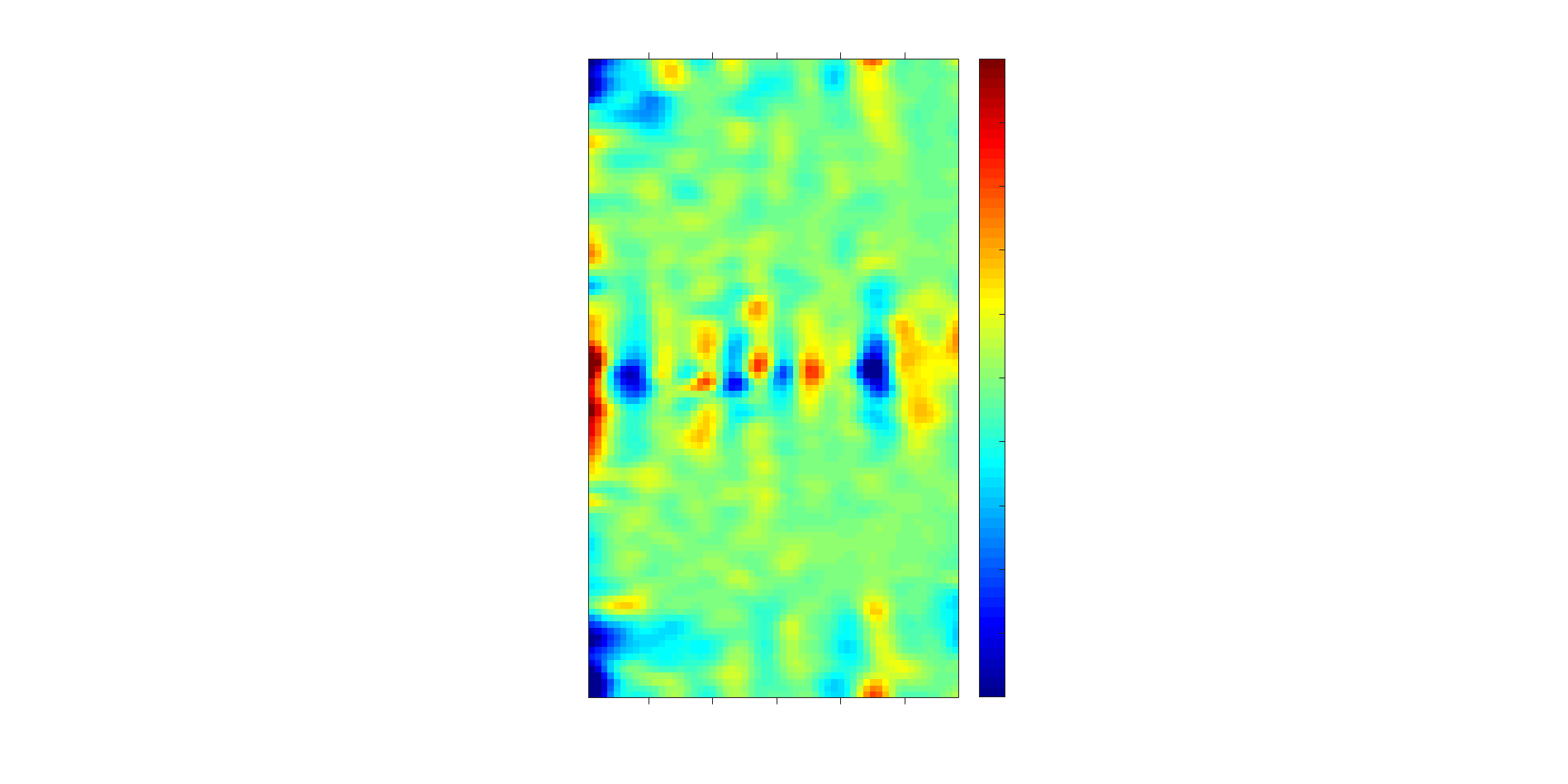

Supplement: Figure 3—source data 1. [file elife-69229-fig3-data1.zip › Figure3 Supp/Nocodazole/c0020_pos5__RawMorpho_noLabel_25.png]

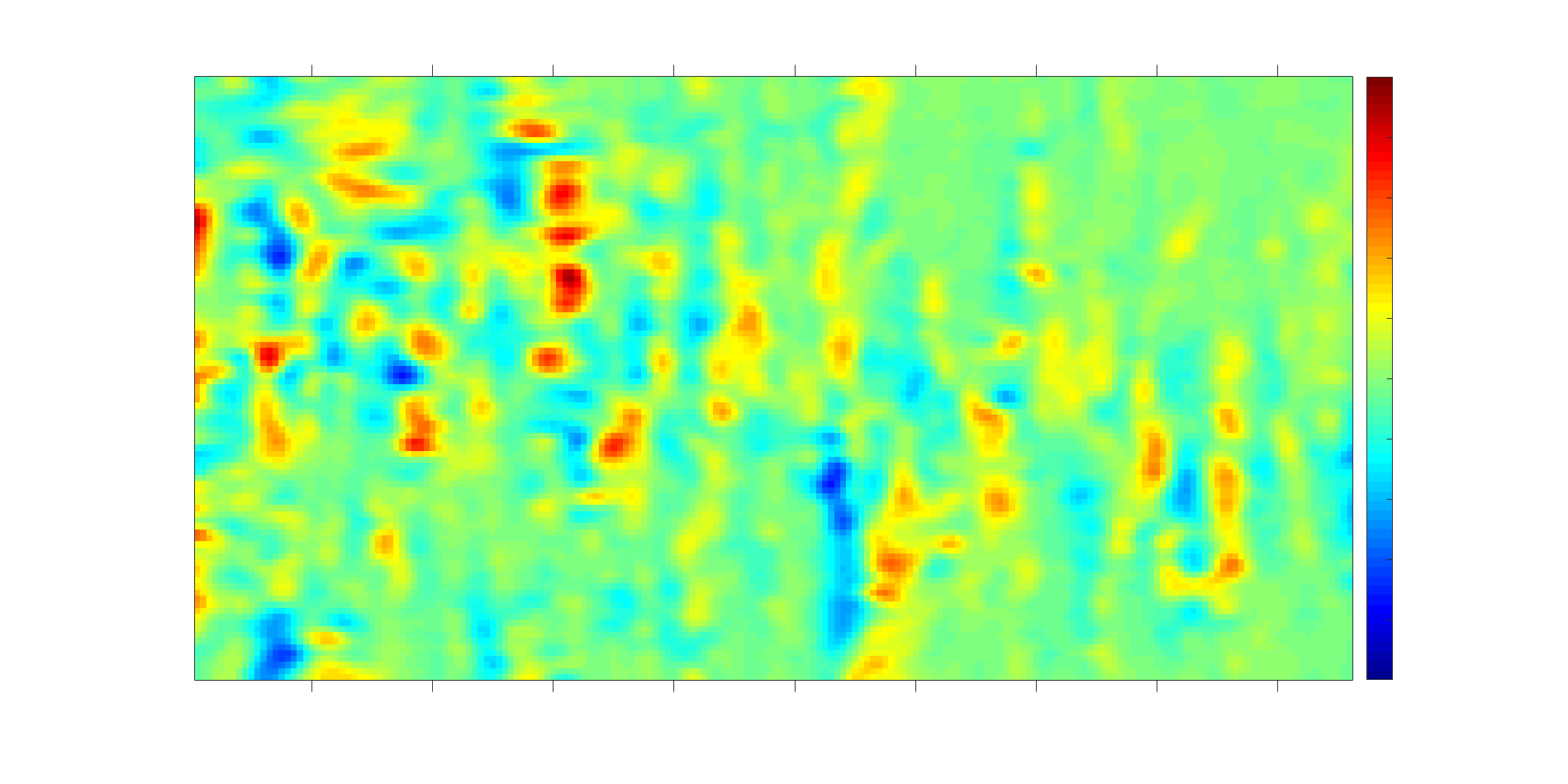

Supplement: Figure 3—source data 1. [file elife-69229-fig3-data1.zip › Figure3 Supp/Nocodazole/c0020_pos6__RawMorpho_noLabel_25.png]

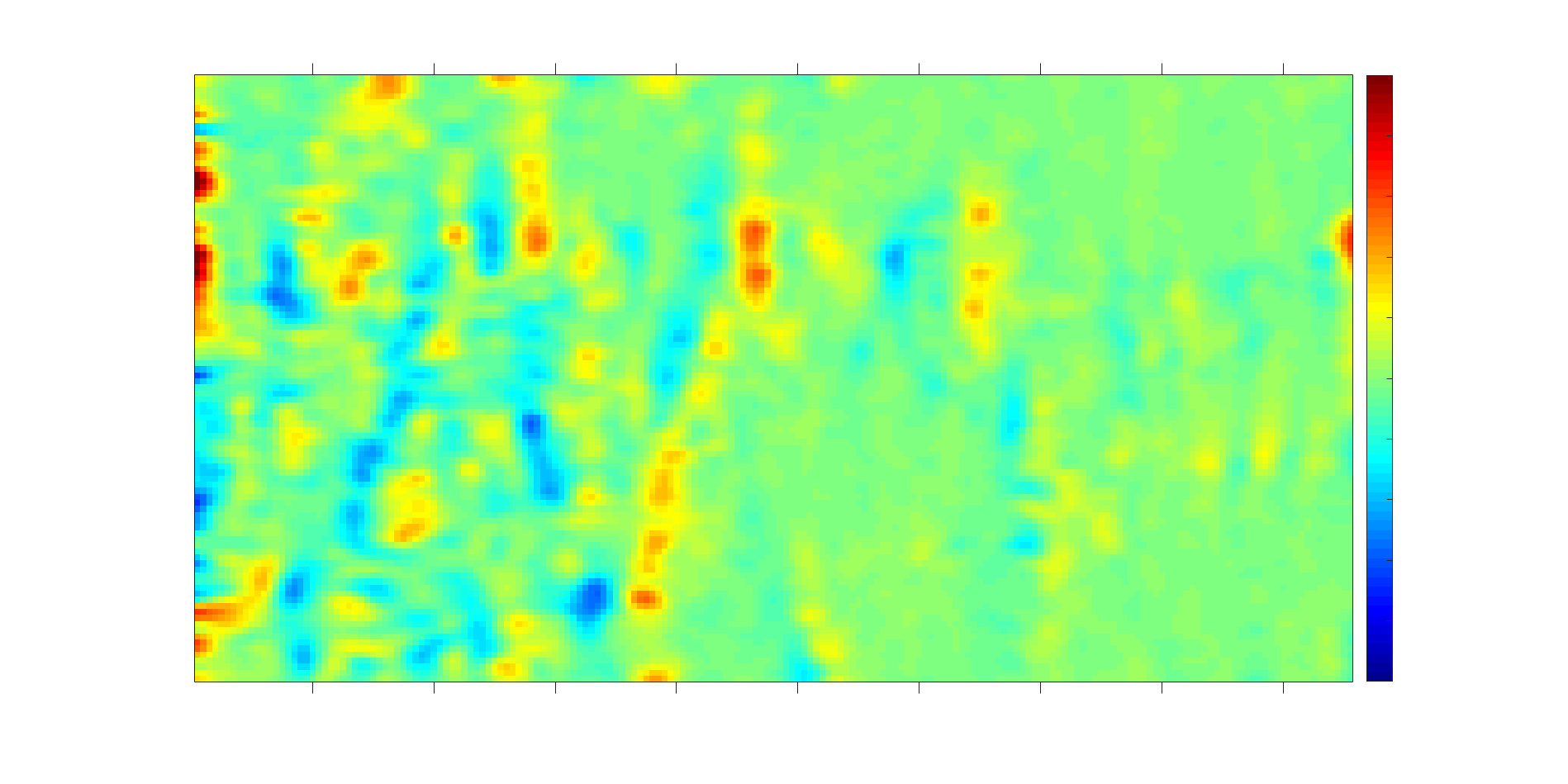

Supplement: Figure 3—source data 1. [file elife-69229-fig3-data1.zip › Figure3 Supp/Nocodazole/c0020_pos7__RawMorpho_noLabel_25.png]

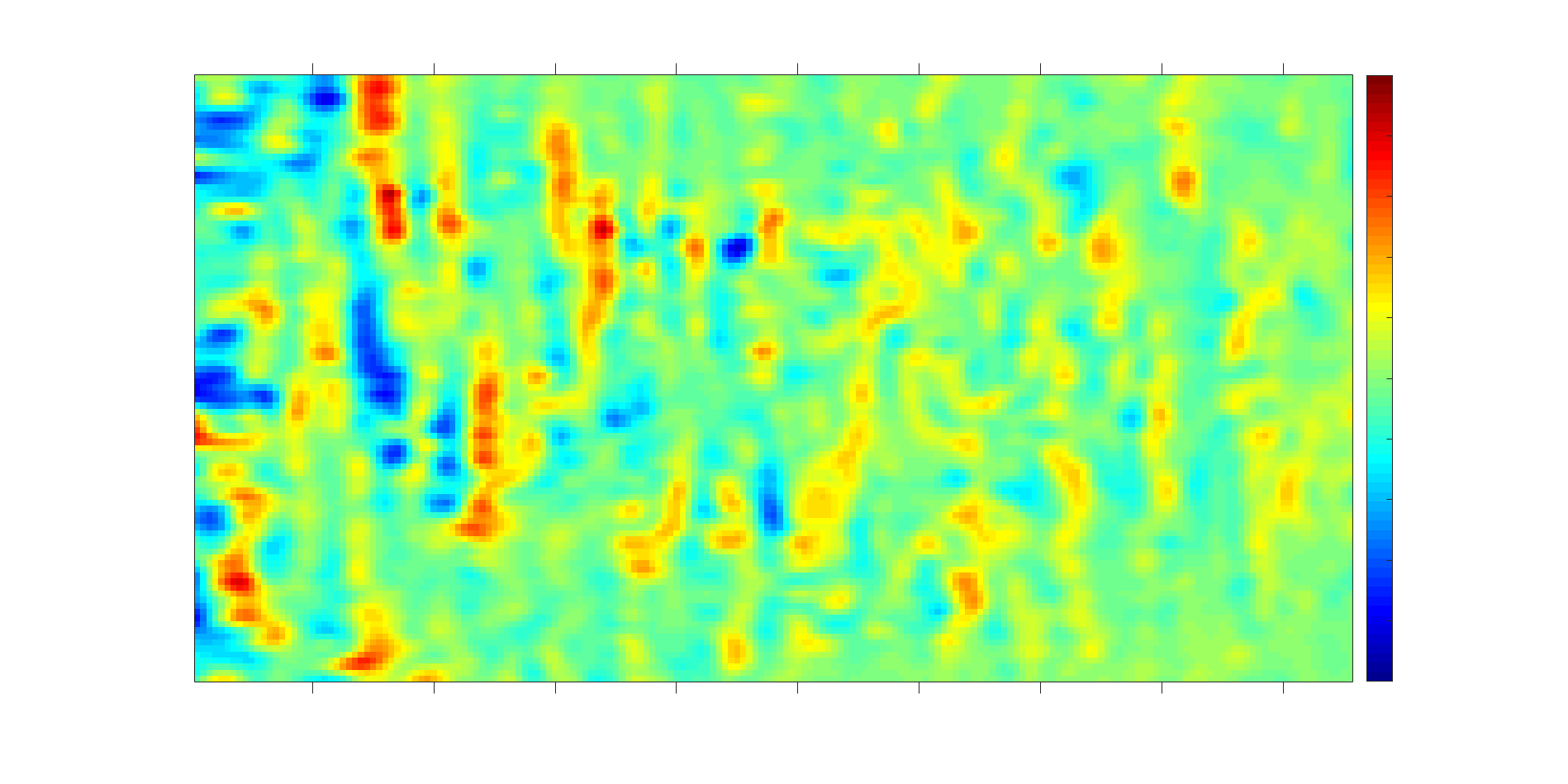

Supplement: Figure 3—source data 1. [file elife-69229-fig3-data1.zip › Figure3 Supp/Nocodazole/c0020_pos8__RawMorpho_noLabel_25.png]

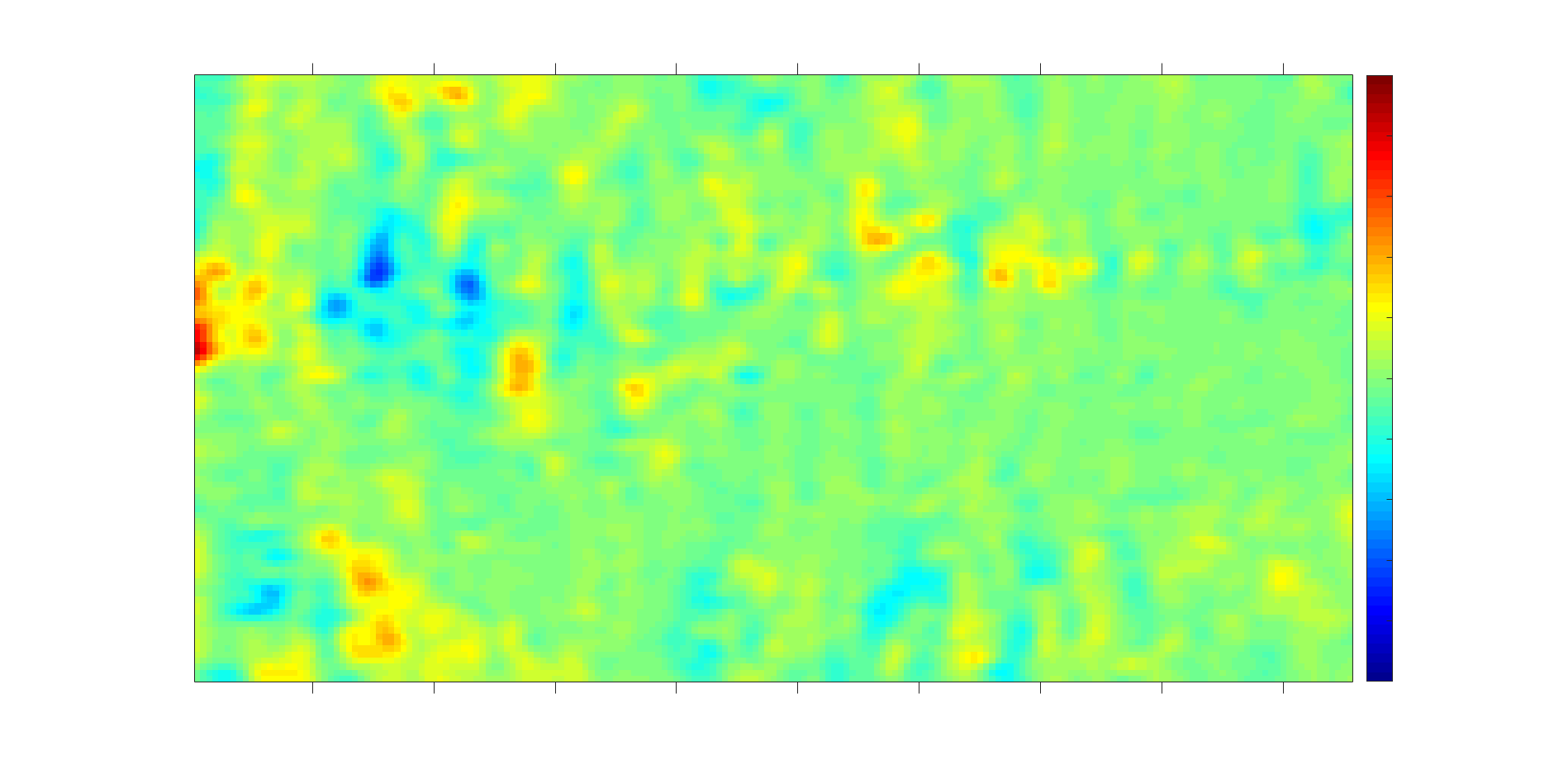

Supplement: Figure 3—source data 1. [file elife-69229-fig3-data1.zip › Figure3 Supp/Nocodazole/c0020_pos9__RawMorpho_noLabel_25.png]

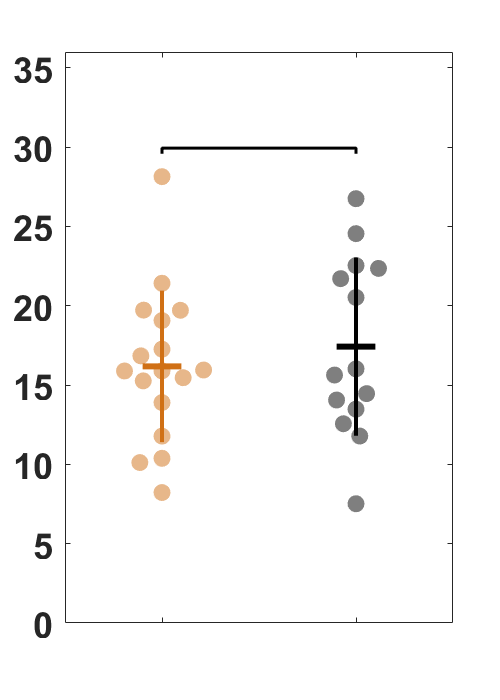

Supplement: Figure 3—source data 1. [file elife-69229-fig3-data1.zip › Figure3/3c/protrusion_speed_BOK_18_Ctrl_NZ_noLabel.png]

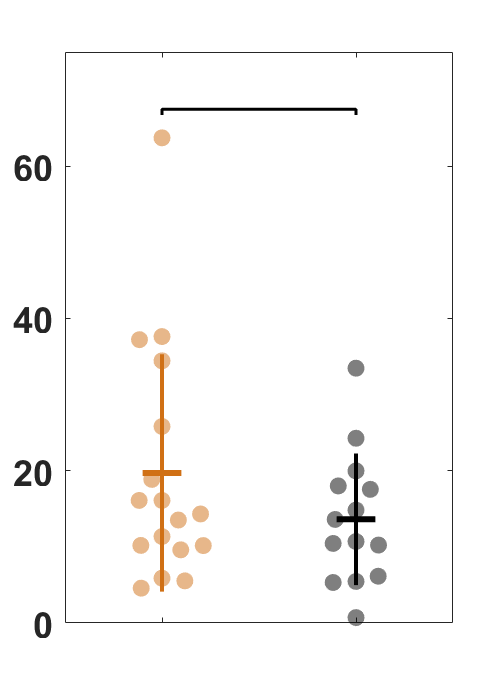

Supplement: Figure 3—source data 1. [file elife-69229-fig3-data1.zip › Figure3/3d/averaged_speed_BOK_81fr_18_Ctrl_NZ_WMW.png]

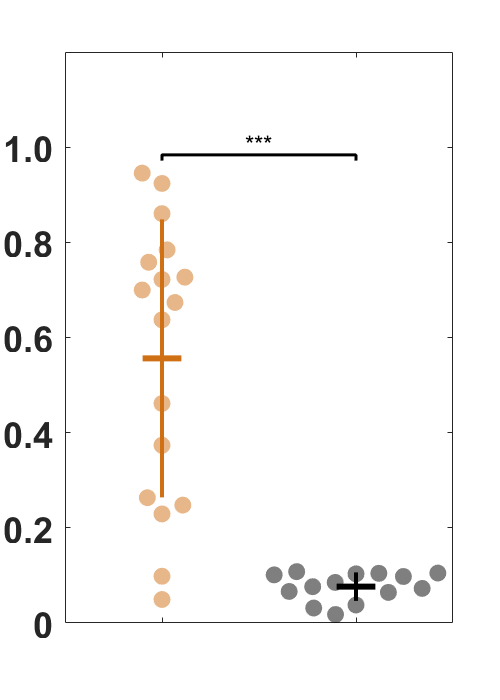

Supplement: Figure 3—source data 1. [file elife-69229-fig3-data1.zip › Figure3/3e/directionality_ratio_BOK_18_Ctrl_NZ_WMW.png]

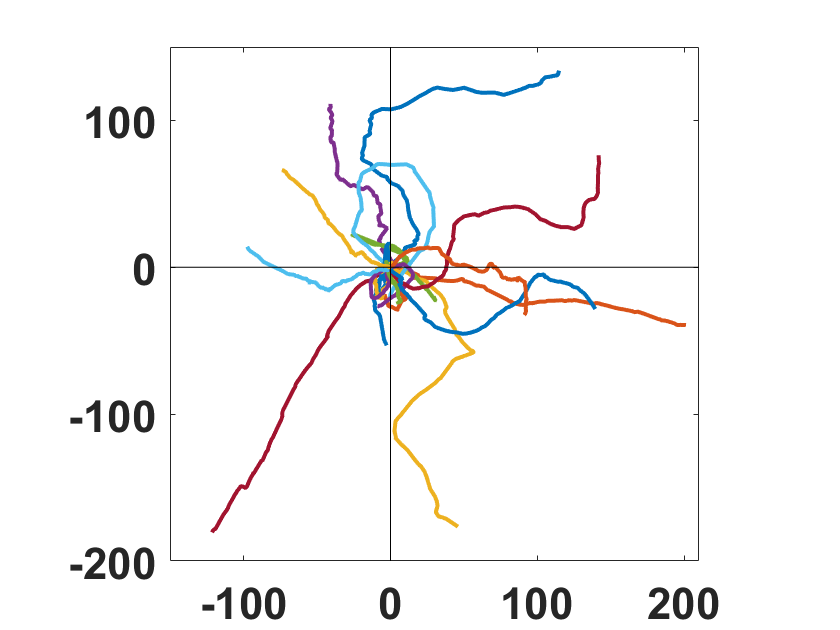

Supplement: Figure 3—source data 1. [file elife-69229-fig3-data1.zip › Figure3/3f/FREELY_trajectories_81fr_um_22.png]

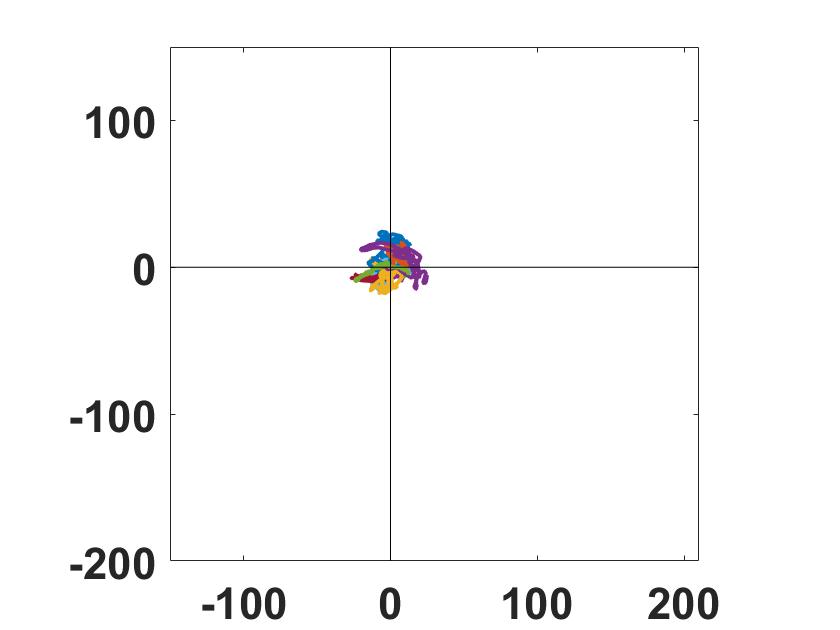

Supplement: Figure 3—source data 1. [file elife-69229-fig3-data1.zip › Figure3/3g/FREELY_Nocodazole_trajectories_81fr_um_22.png]

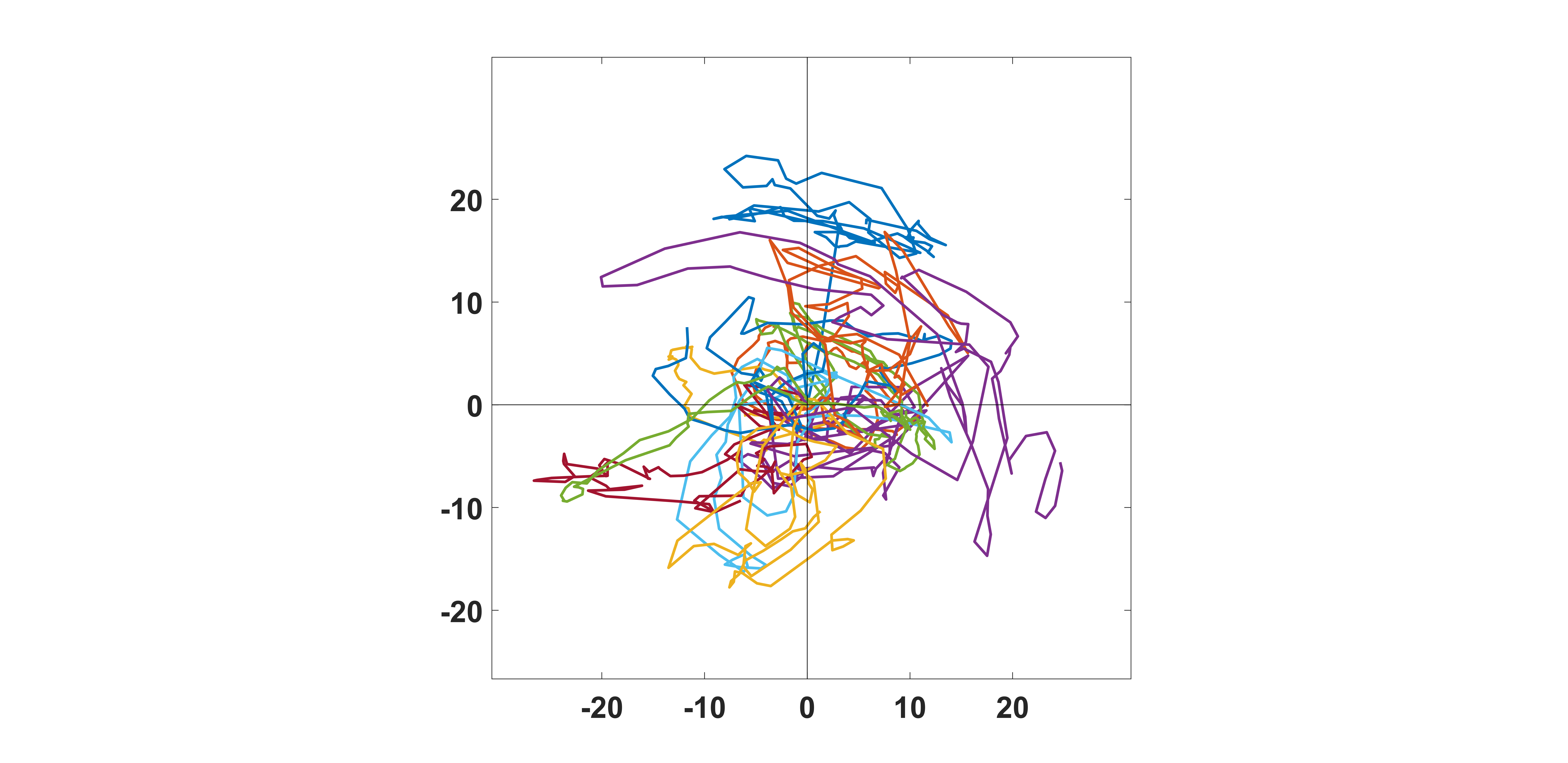

Supplement: Figure 3—source data 1. [file elife-69229-fig3-data1.zip › Figure3/3g/FREELY_Nocodazole_trajectories_81fr_um_22_ZOOM.png]

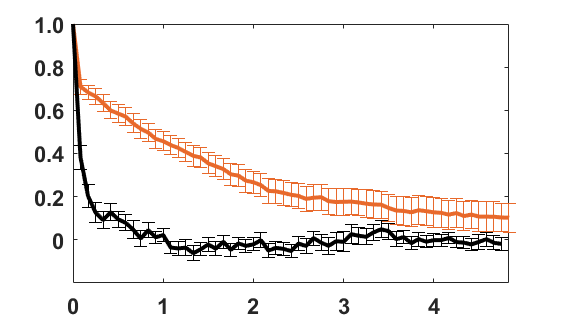

Supplement: Figure 3—source data 1. [file elife-69229-fig3-data1.zip › Figure3/3h-i/autocorrelation_BOK_11_Ctrl_NZ.png]

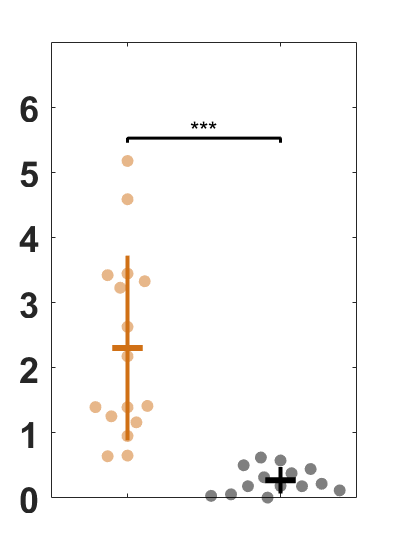

Supplement: Figure 3—source data 1. [file elife-69229-fig3-data1.zip › Figure3/3h-i/persistence_time_BOK_18_Ctrl_NZ.png]

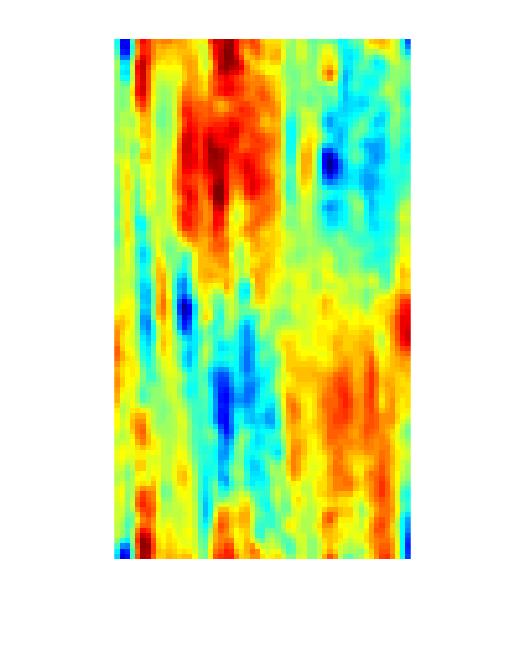

Supplement: Figure 4—source data 1. [file elife-69229-fig4-data1.zip › Figure4 Supp/Figure4 - figure supplement2/morphomap data/pos10.mat1.jpg]

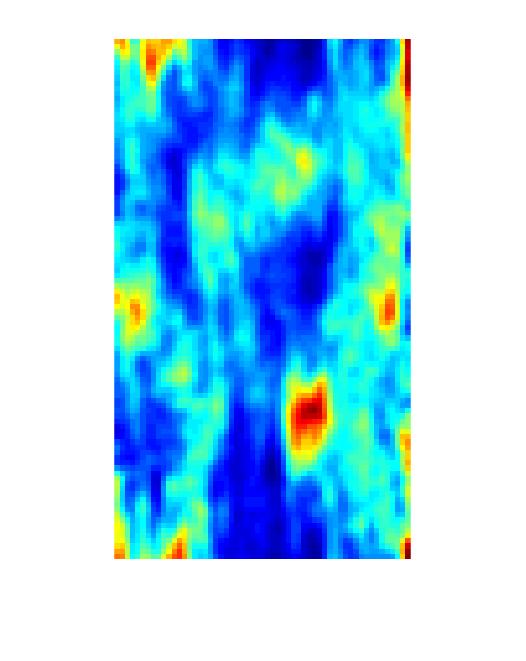

Supplement: Figure 4—source data 1. [file elife-69229-fig4-data1.zip › Figure4 Supp/Figure4 - figure supplement2/morphomap data/pos10.mat2.jpg]

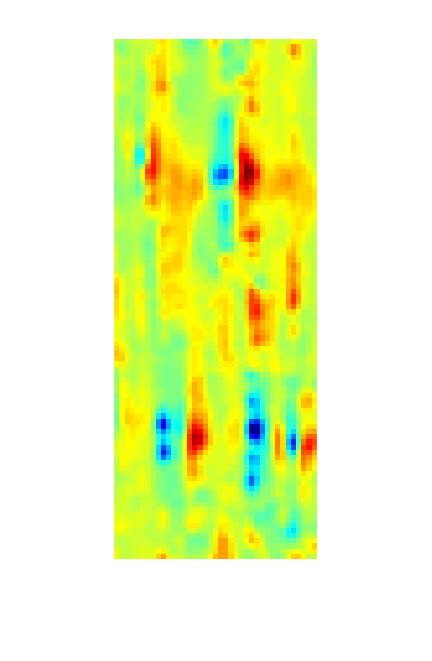

Supplement: Figure 4—source data 1. [file elife-69229-fig4-data1.zip › Figure4 Supp/Figure4 - figure supplement2/morphomap data/pos11.mat1.jpg]

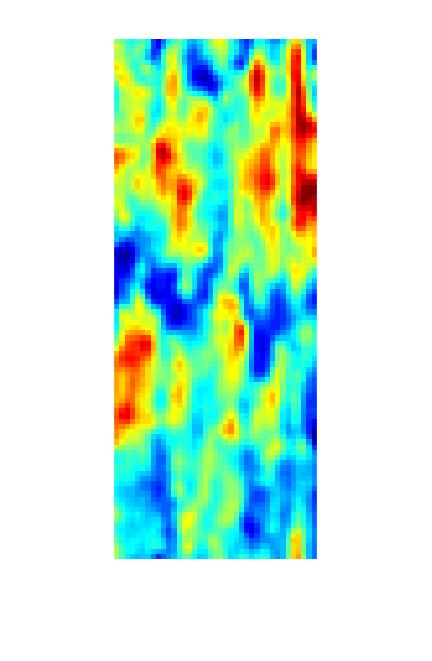

Supplement: Figure 4—source data 1. [file elife-69229-fig4-data1.zip › Figure4 Supp/Figure4 - figure supplement2/morphomap data/pos11.mat2.jpg]

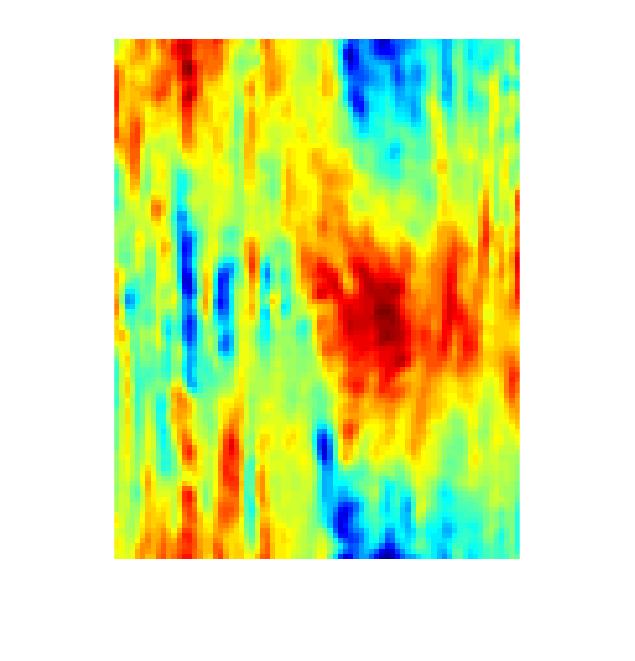

Supplement: Figure 4—source data 1. [file elife-69229-fig4-data1.zip › Figure4 Supp/Figure4 - figure supplement2/morphomap data/pos12.mat1.jpg]

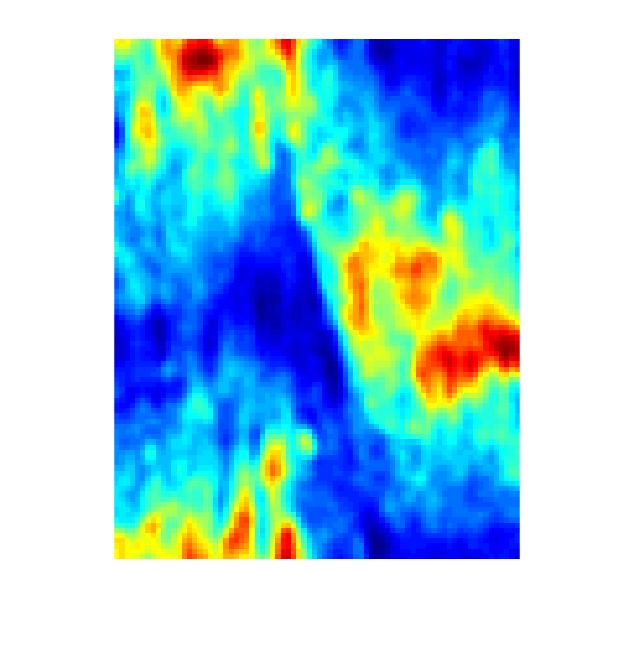

Supplement: Figure 4—source data 1. [file elife-69229-fig4-data1.zip › Figure4 Supp/Figure4 - figure supplement2/morphomap data/pos12.mat2.jpg]

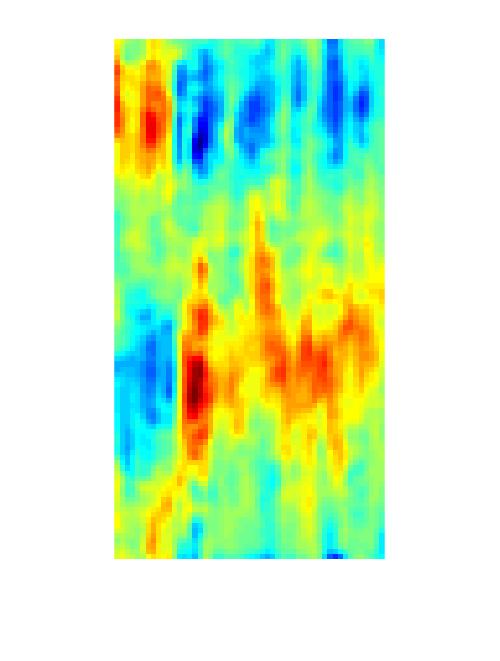

Supplement: Figure 4—source data 1. [file elife-69229-fig4-data1.zip › Figure4 Supp/Figure4 - figure supplement2/morphomap data/pos13.mat1.jpg]

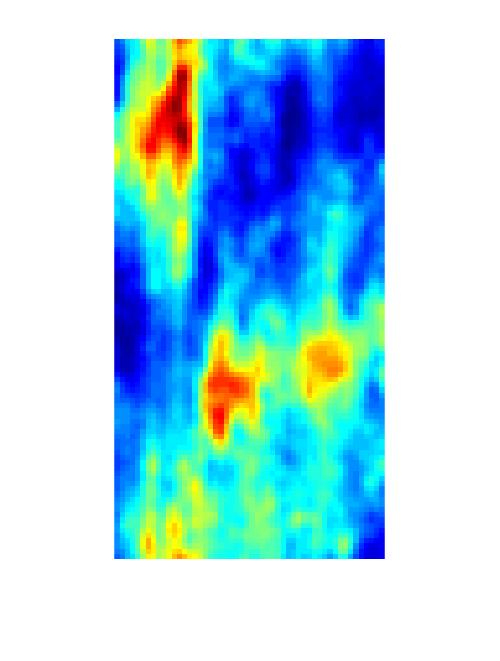

Supplement: Figure 4—source data 1. [file elife-69229-fig4-data1.zip › Figure4 Supp/Figure4 - figure supplement2/morphomap data/pos13.mat2.jpg]

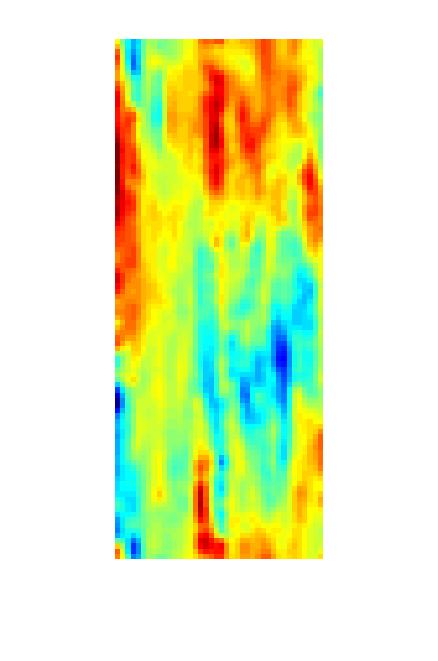

Supplement: Figure 4—source data 1. [file elife-69229-fig4-data1.zip › Figure4 Supp/Figure4 - figure supplement2/morphomap data/pos14.mat1.jpg]

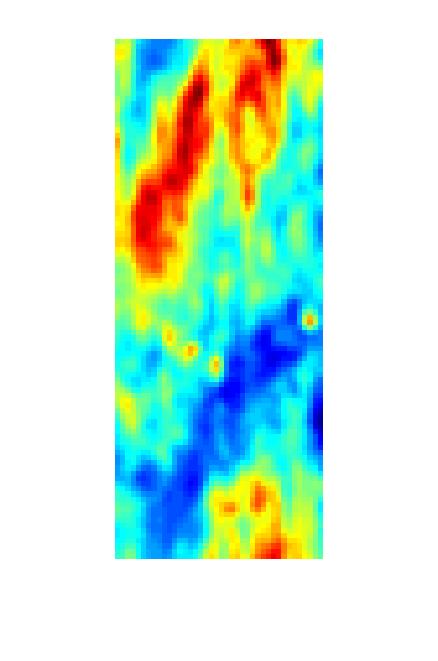

Supplement: Figure 4—source data 1. [file elife-69229-fig4-data1.zip › Figure4 Supp/Figure4 - figure supplement2/morphomap data/pos14.mat2.jpg]

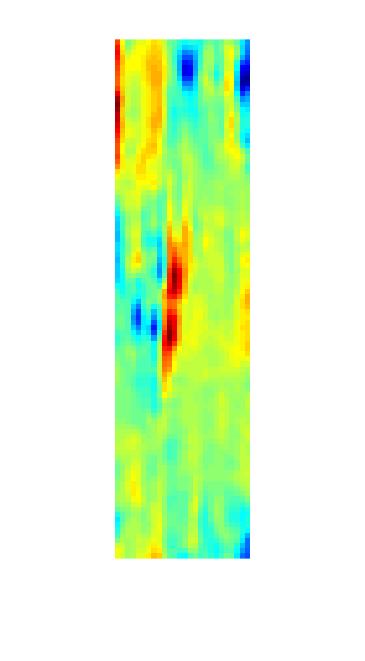

Supplement: Figure 4—source data 1. [file elife-69229-fig4-data1.zip › Figure4 Supp/Figure4 - figure supplement2/morphomap data/pos16.mat1.jpg]

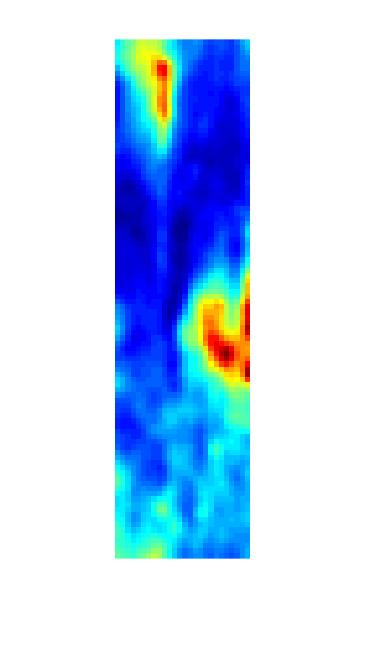

Supplement: Figure 4—source data 1. [file elife-69229-fig4-data1.zip › Figure4 Supp/Figure4 - figure supplement2/morphomap data/pos16.mat2.jpg]

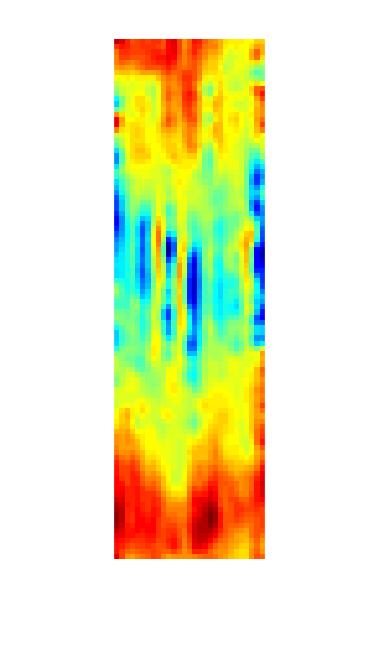

Supplement: Figure 4—source data 1. [file elife-69229-fig4-data1.zip › Figure4 Supp/Figure4 - figure supplement2/morphomap data/pos17.mat1.jpg]

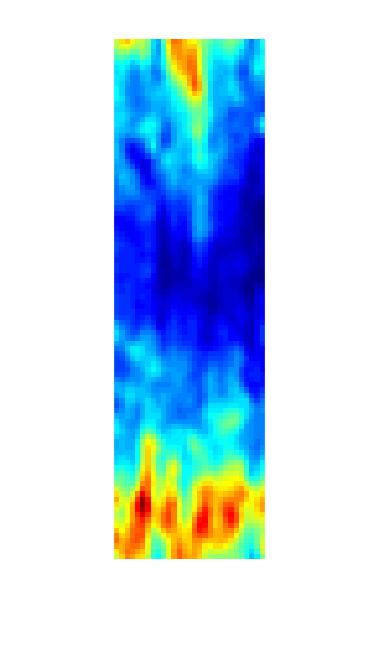

Supplement: Figure 4—source data 1. [file elife-69229-fig4-data1.zip › Figure4 Supp/Figure4 - figure supplement2/morphomap data/pos17.mat2.jpg]

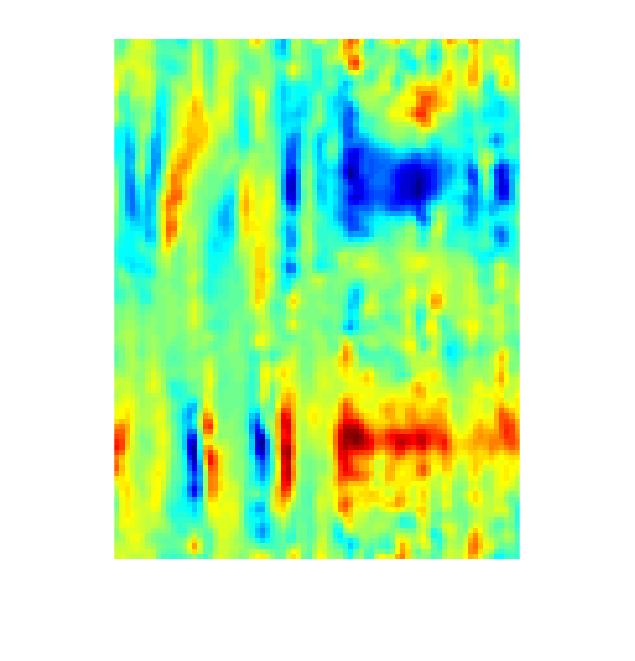

Supplement: Figure 4—source data 1. [file elife-69229-fig4-data1.zip › Figure4 Supp/Figure4 - figure supplement2/morphomap data/pos18.mat1.jpg]

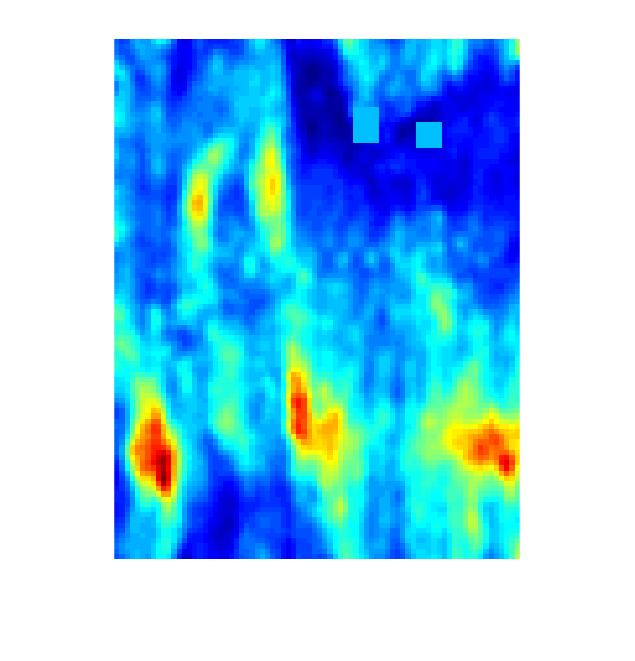

Supplement: Figure 4—source data 1. [file elife-69229-fig4-data1.zip › Figure4 Supp/Figure4 - figure supplement2/morphomap data/pos18.mat2.jpg]

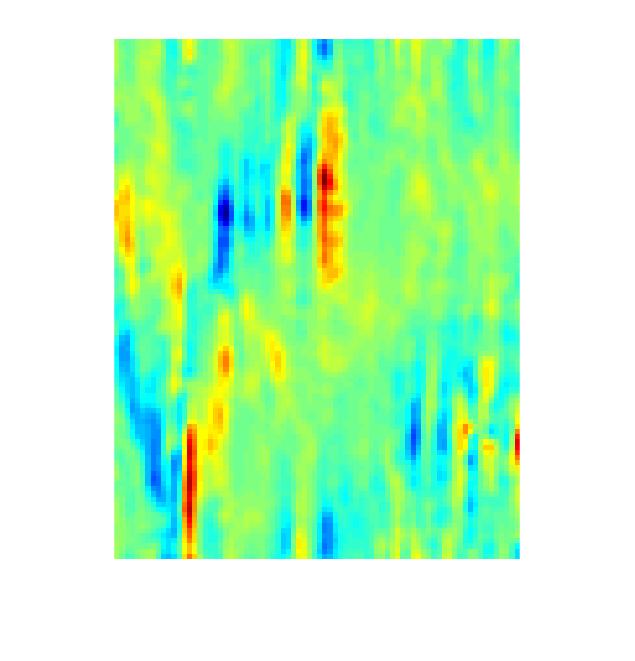

Supplement: Figure 4—source data 1. [file elife-69229-fig4-data1.zip › Figure4 Supp/Figure4 - figure supplement2/morphomap data/pos19.mat1.jpg]

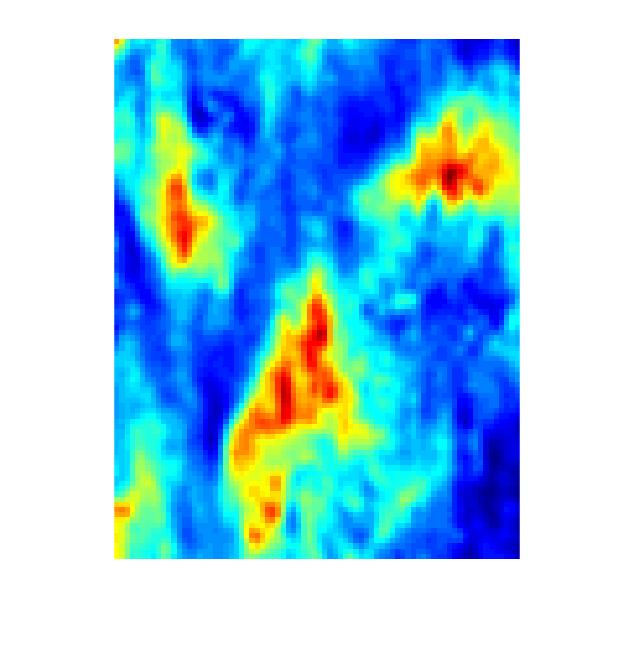

Supplement: Figure 4—source data 1. [file elife-69229-fig4-data1.zip › Figure4 Supp/Figure4 - figure supplement2/morphomap data/pos19.mat2.jpg]

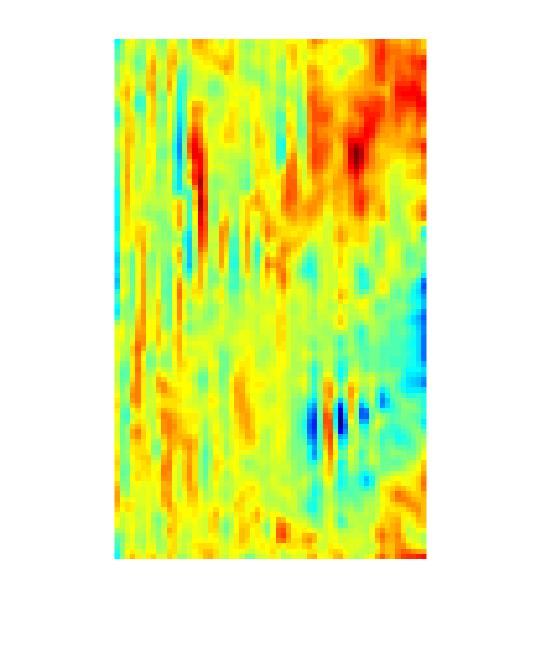

Supplement: Figure 4—source data 1. [file elife-69229-fig4-data1.zip › Figure4 Supp/Figure4 - figure supplement2/morphomap data/pos2.mat1.jpg]

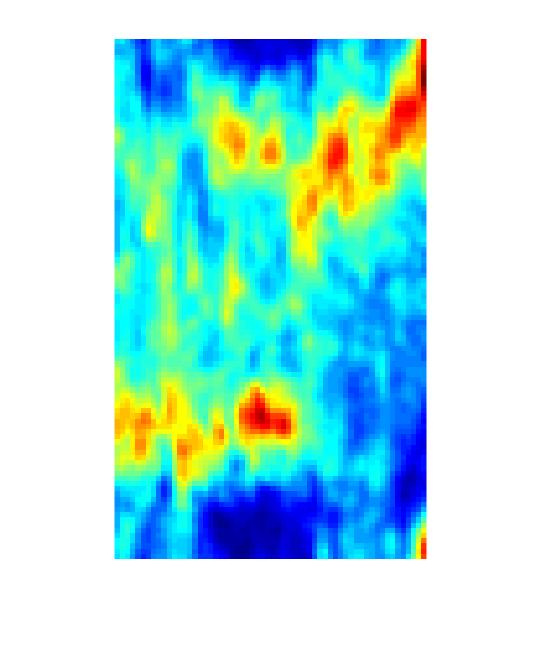

Supplement: Figure 4—source data 1. [file elife-69229-fig4-data1.zip › Figure4 Supp/Figure4 - figure supplement2/morphomap data/pos2.mat2.jpg]

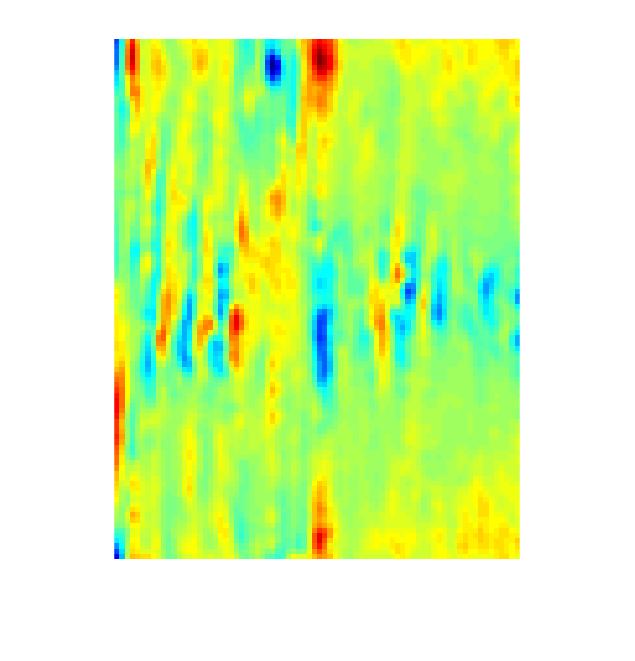

Supplement: Figure 4—source data 1. [file elife-69229-fig4-data1.zip › Figure4 Supp/Figure4 - figure supplement2/morphomap data/pos20.mat1.jpg]

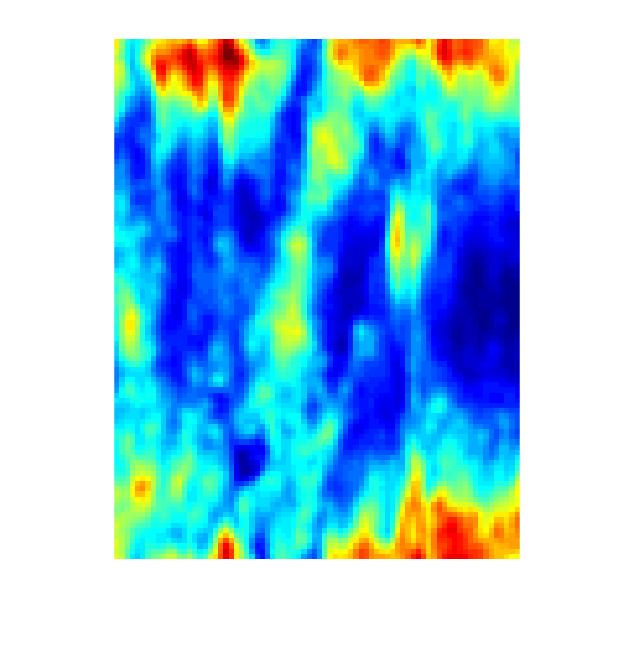

Supplement: Figure 4—source data 1. [file elife-69229-fig4-data1.zip › Figure4 Supp/Figure4 - figure supplement2/morphomap data/pos20.mat2.jpg]

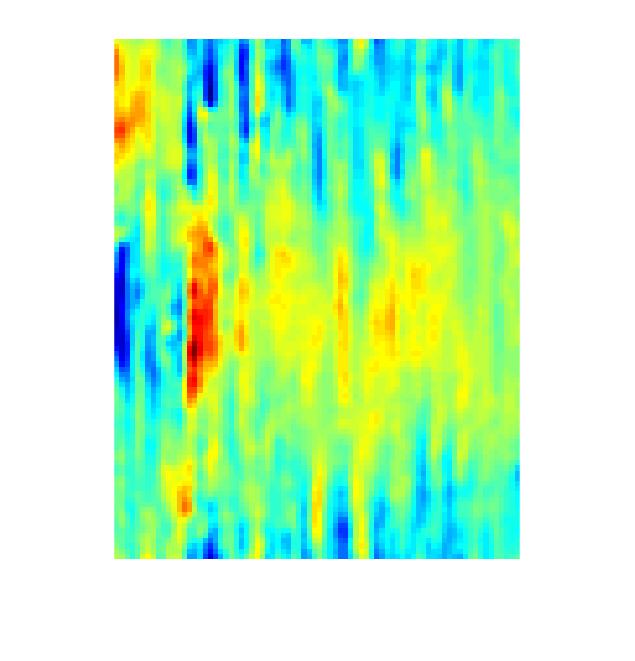

Supplement: Figure 4—source data 1. [file elife-69229-fig4-data1.zip › Figure4 Supp/Figure4 - figure supplement2/morphomap data/pos4.mat1.jpg]

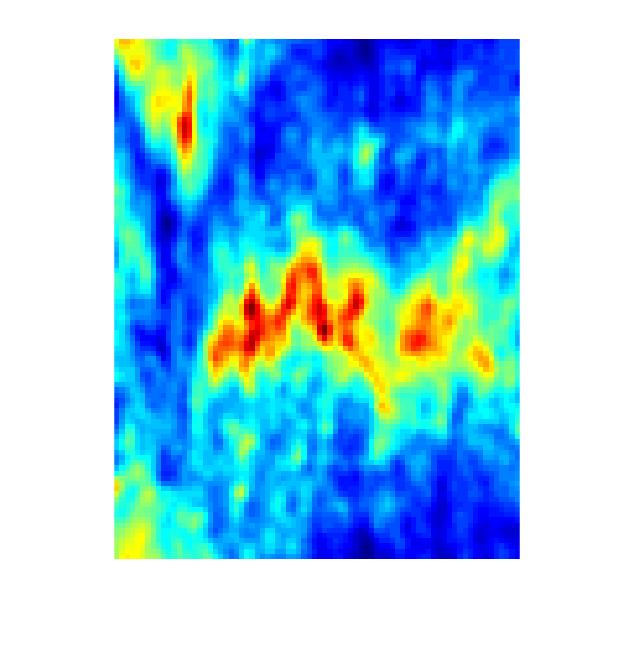

Supplement: Figure 4—source data 1. [file elife-69229-fig4-data1.zip › Figure4 Supp/Figure4 - figure supplement2/morphomap data/pos4.mat2.jpg]

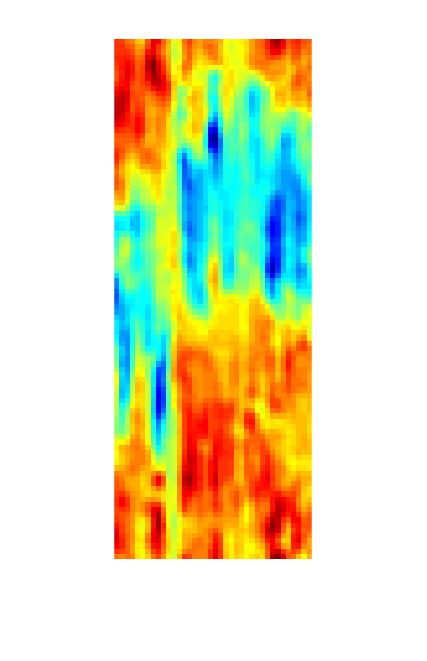

Supplement: Figure 4—source data 1. [file elife-69229-fig4-data1.zip › Figure4 Supp/Figure4 - figure supplement2/morphomap data/pos6.mat1.jpg]

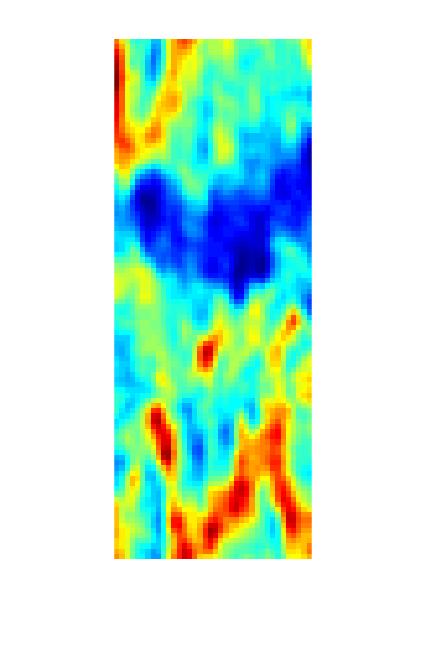

Supplement: Figure 4—source data 1. [file elife-69229-fig4-data1.zip › Figure4 Supp/Figure4 - figure supplement2/morphomap data/pos6.mat2.jpg]

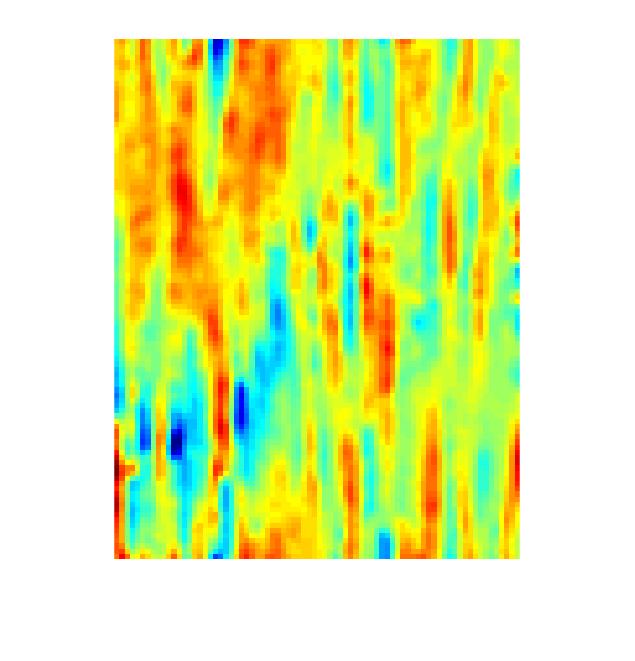

Supplement: Figure 4—source data 1. [file elife-69229-fig4-data1.zip › Figure4 Supp/Figure4 - figure supplement2/morphomap data/pos7.mat1.jpg]

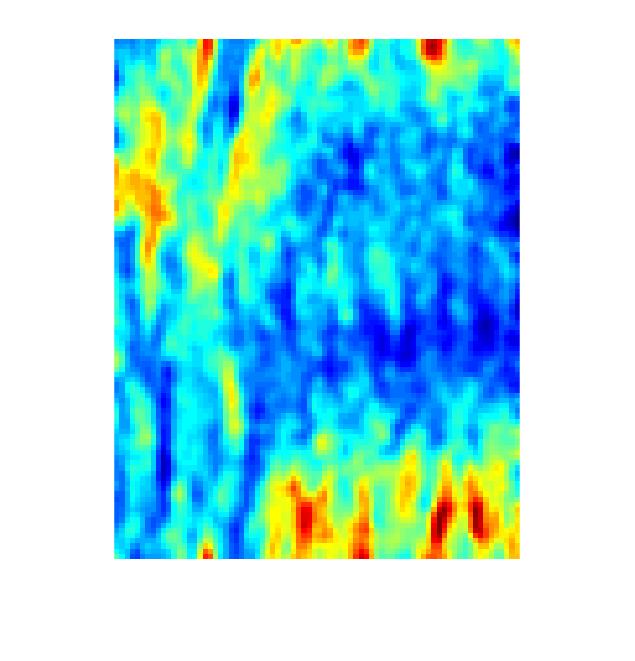

Supplement: Figure 4—source data 1. [file elife-69229-fig4-data1.zip › Figure4 Supp/Figure4 - figure supplement2/morphomap data/pos7.mat2.jpg]

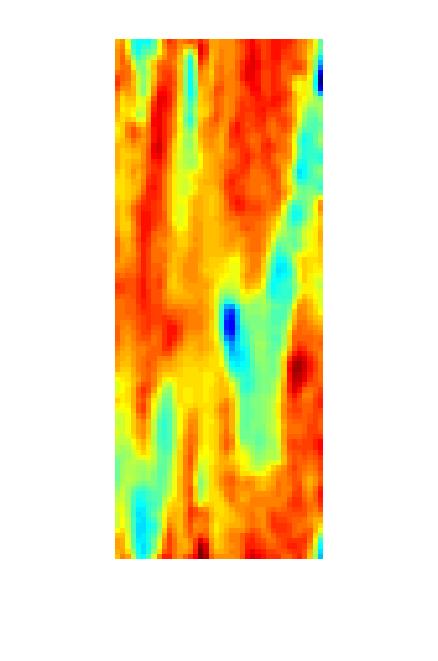

Supplement: Figure 4—source data 1. [file elife-69229-fig4-data1.zip › Figure4 Supp/Figure4 - figure supplement2/morphomap data/pos9.mat1.jpg]

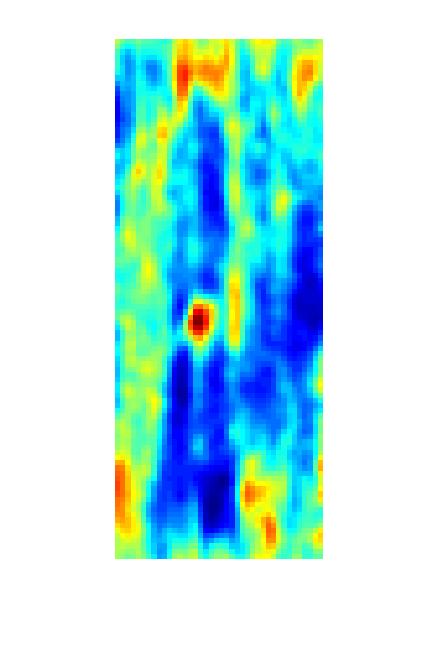

Supplement: Figure 4—source data 1. [file elife-69229-fig4-data1.zip › Figure4 Supp/Figure4 - figure supplement2/morphomap data/pos9.mat2.jpg]

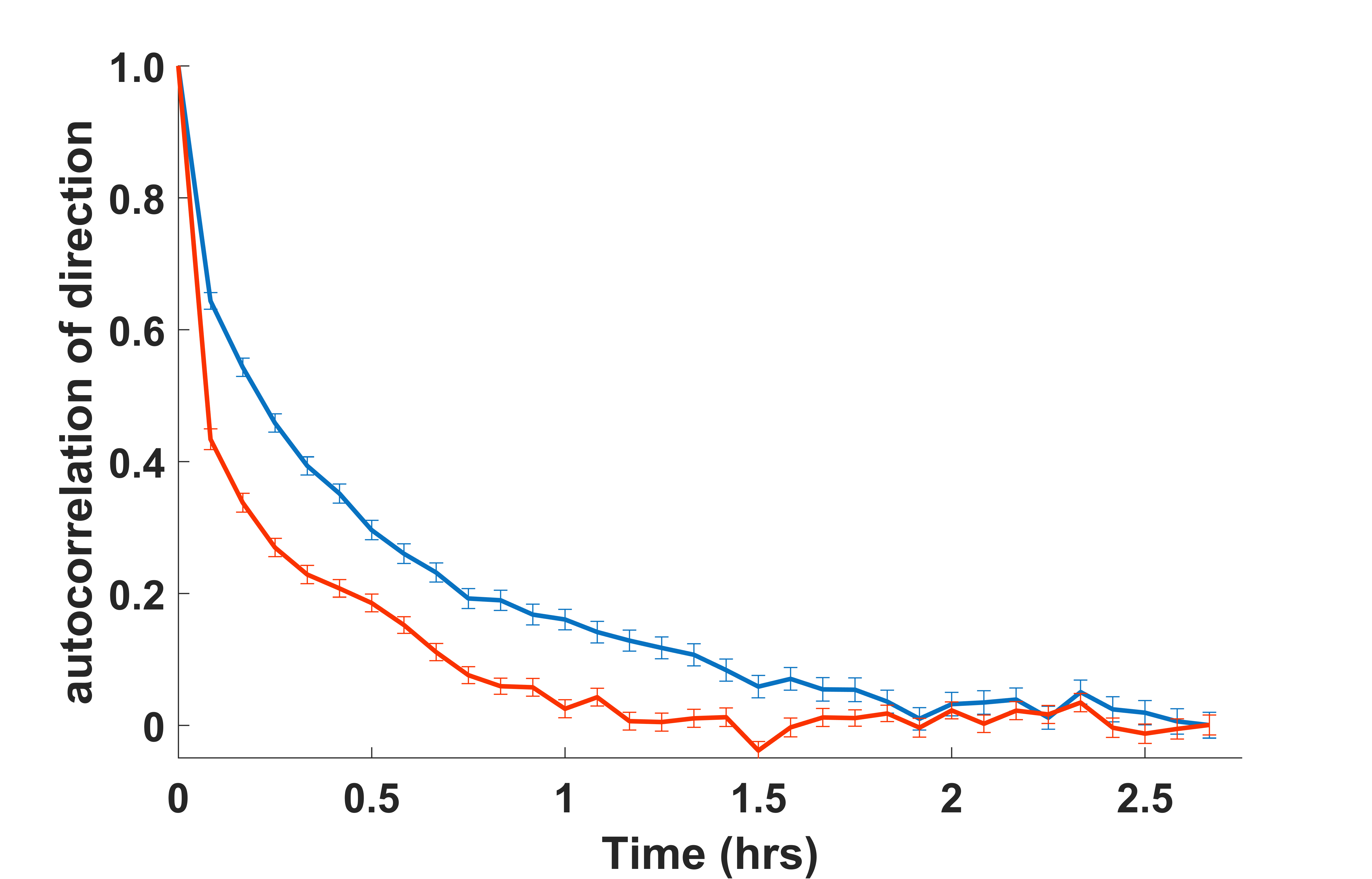

Supplement: Figure 4—source data 1. [file elife-69229-fig4-data1.zip › Figure4 Supp/Figure4 - figure supplement3/auto10x.png]

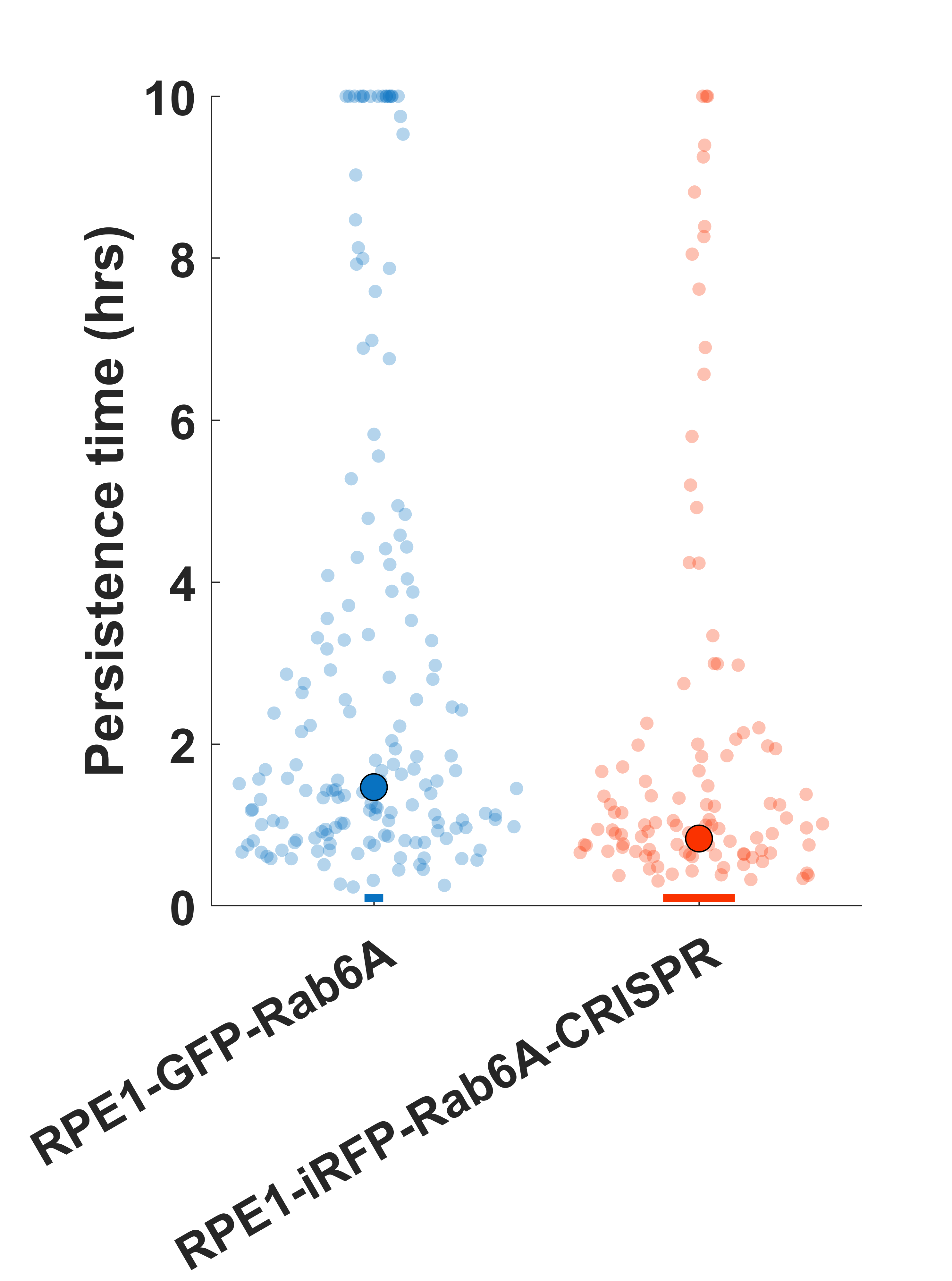

Supplement: Figure 4—source data 1. [file elife-69229-fig4-data1.zip › Figure4 Supp/Figure4 - figure supplement3/persistence10x.png]

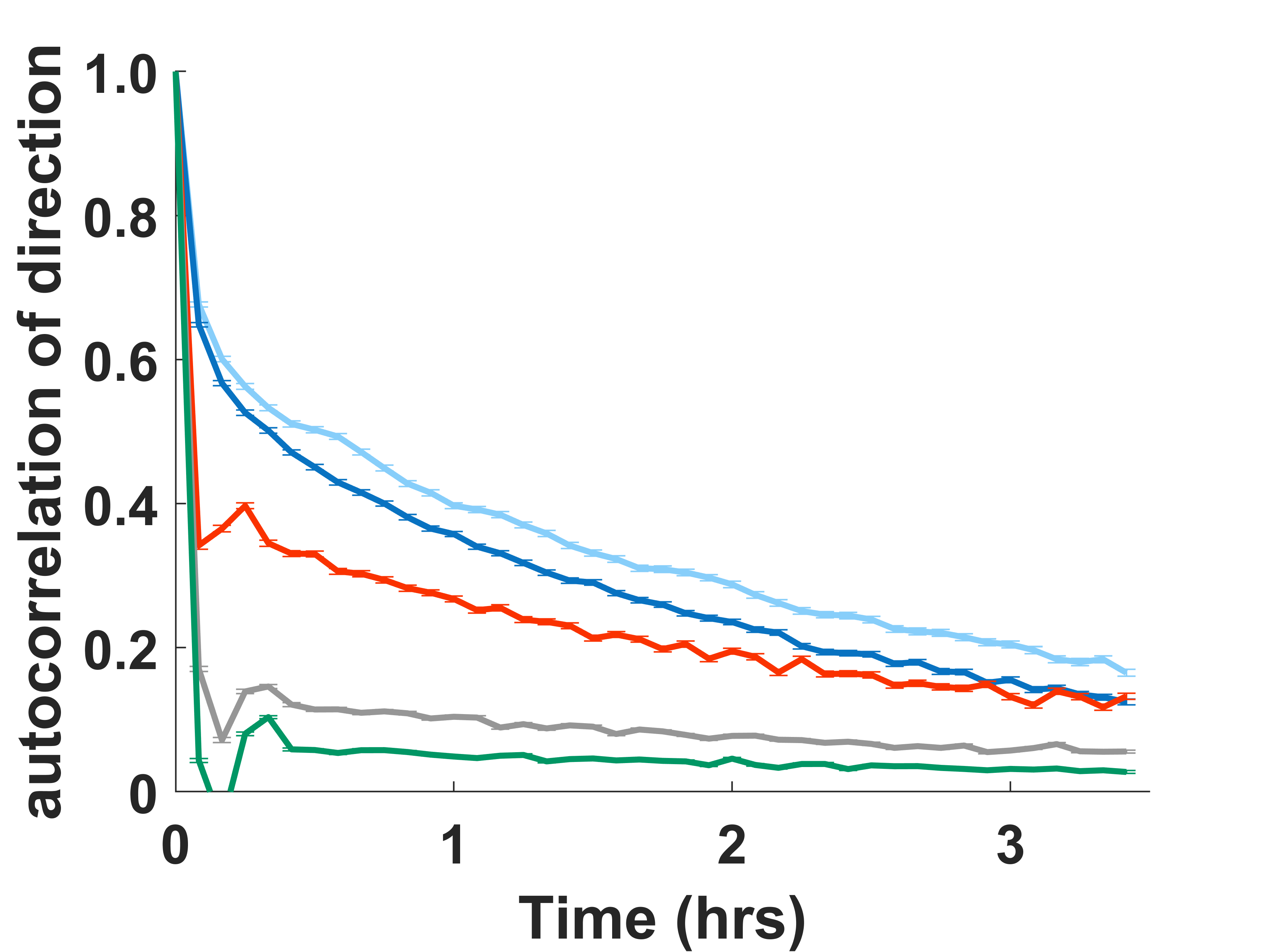

Supplement: Figure 4—source data 1. [file elife-69229-fig4-data1.zip › Figure4 Supp/Figure4 - figure supplement4/autocorr.png]

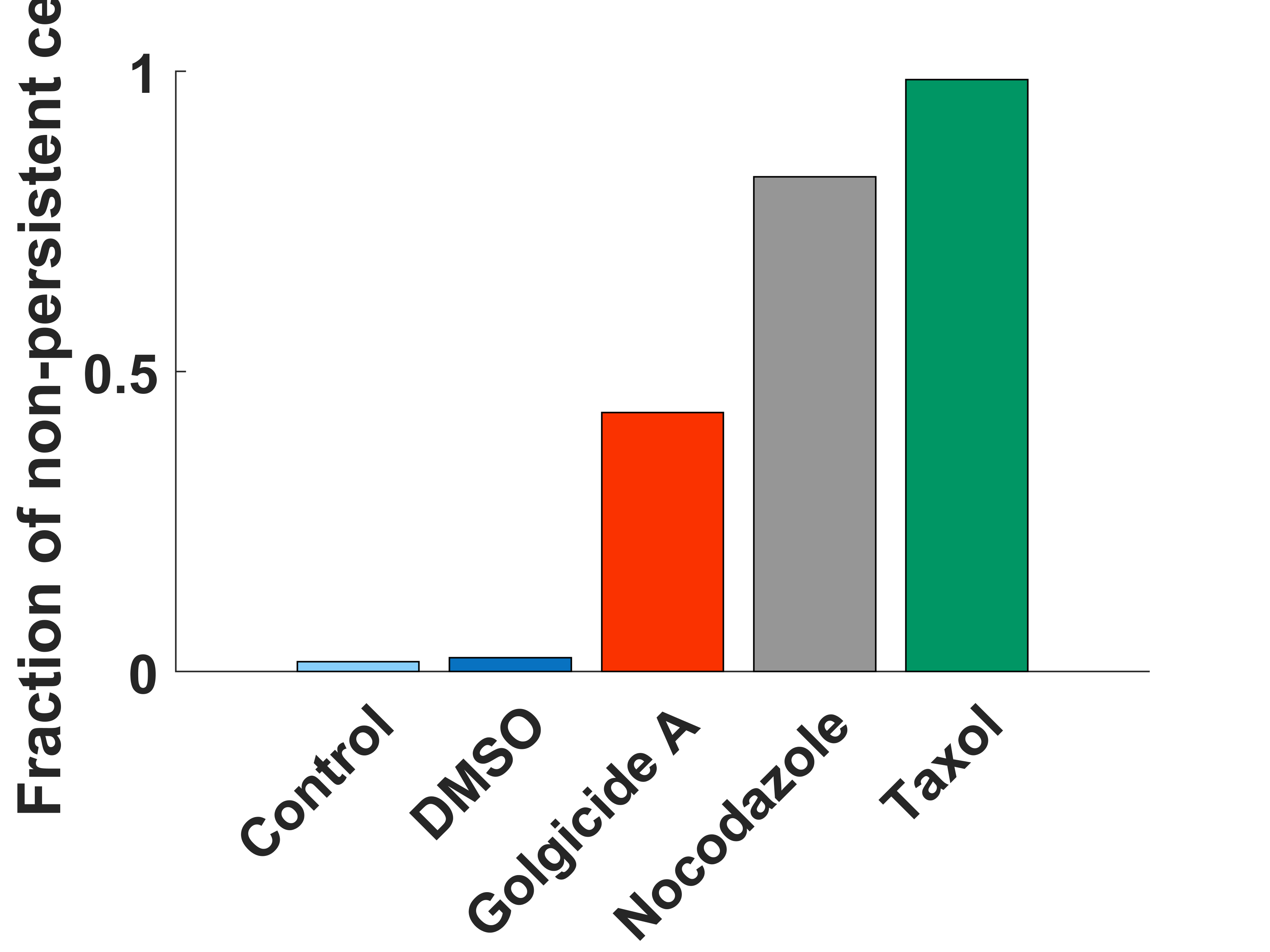

Supplement: Figure 4—source data 1. [file elife-69229-fig4-data1.zip › Figure4 Supp/Figure4 - figure supplement4/boxes.png]

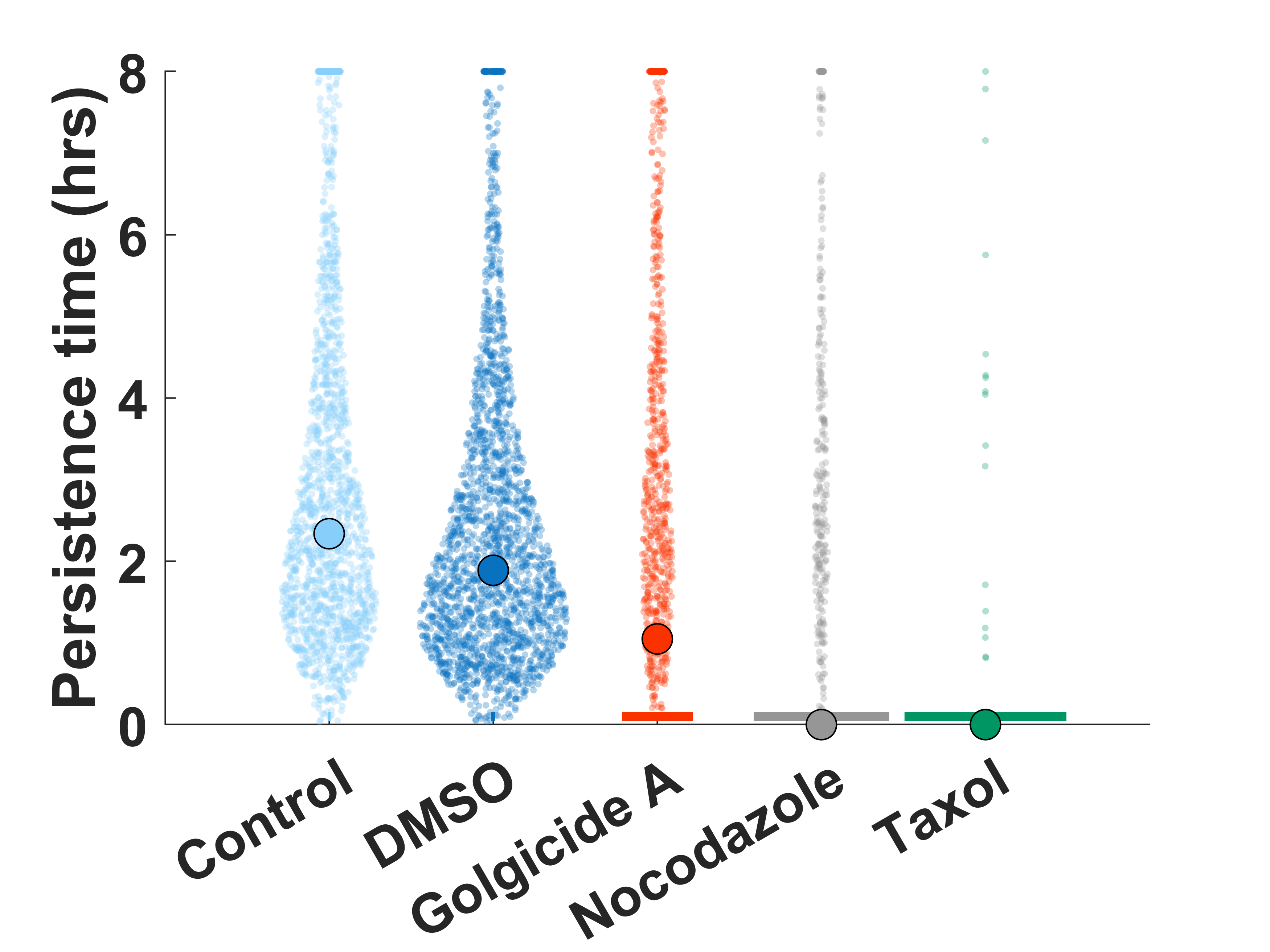

Supplement: Figure 4—source data 1. [file elife-69229-fig4-data1.zip › Figure4 Supp/Figure4 - figure supplement4/swarm.png]

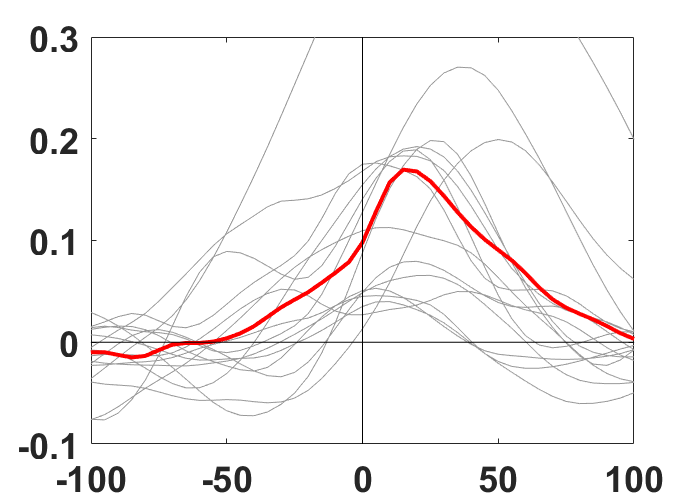

Supplement: Figure 4—source data 1. [file elife-69229-fig4-data1.zip › Figure4/4c-e/corrCoeff_ALL_18_inverse_noLabel.png]

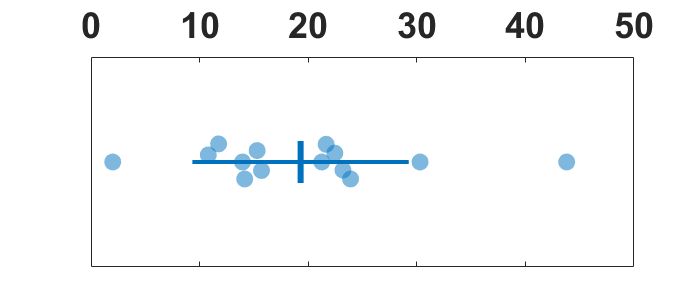

Supplement: Figure 4—source data 1. [file elife-69229-fig4-data1.zip › Figure4/4c-e/lagSwarm_18_noLabel.png]

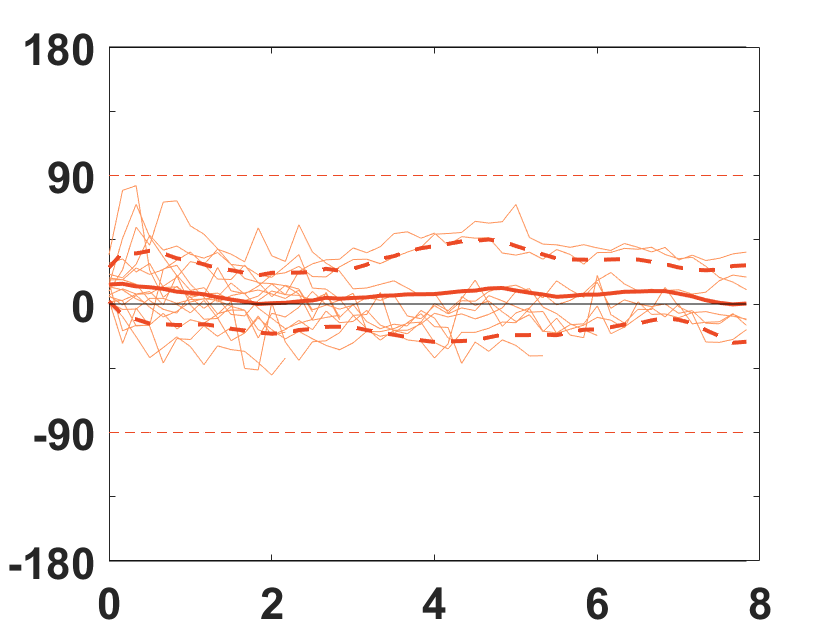

Supplement: Figure 5—source data 1. [file elife-69229-fig5-data1.zip › Figure5 Supp/Figure5b supp - front/FRONT_exp_reorientation_10h_O_22_box.png]

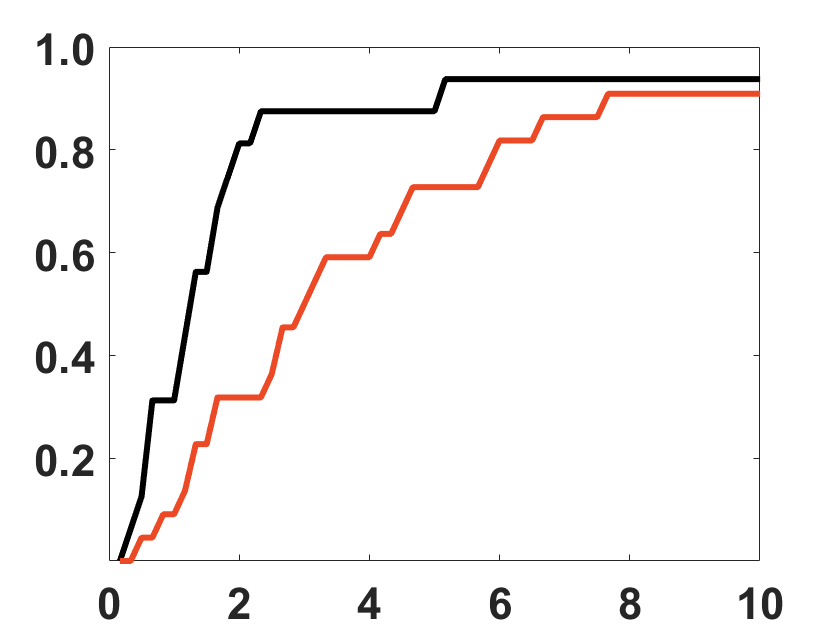

Supplement: Figure 5—source data 1. [file elife-69229-fig5-data1.zip › Figure5 Supp/Figure5c supp - cumulative/cumulative_4f_exp_only.png]

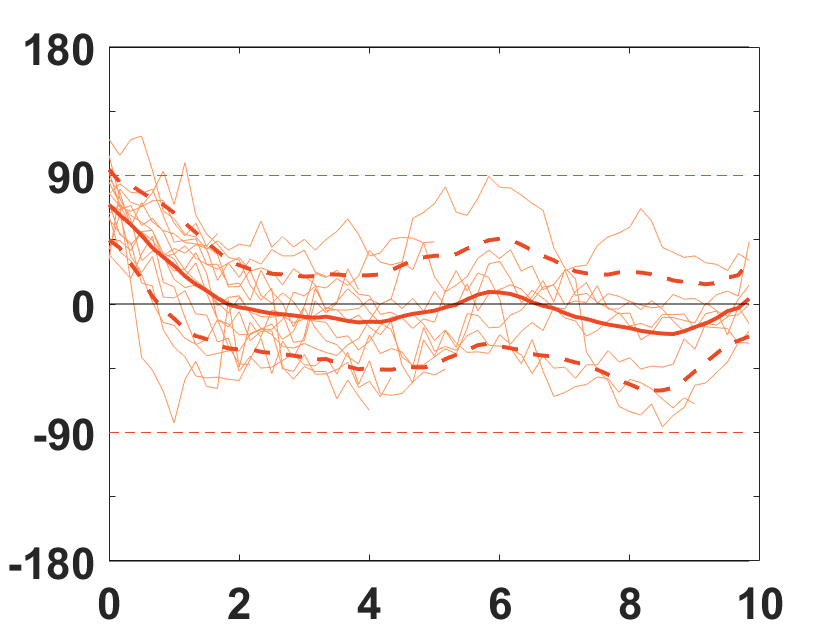

Supplement: Figure 5—source data 1. [file elife-69229-fig5-data1.zip › Figure5 Supp/Figure5e supp - static/STATIC_exp_reorientation_O_22_all.png]

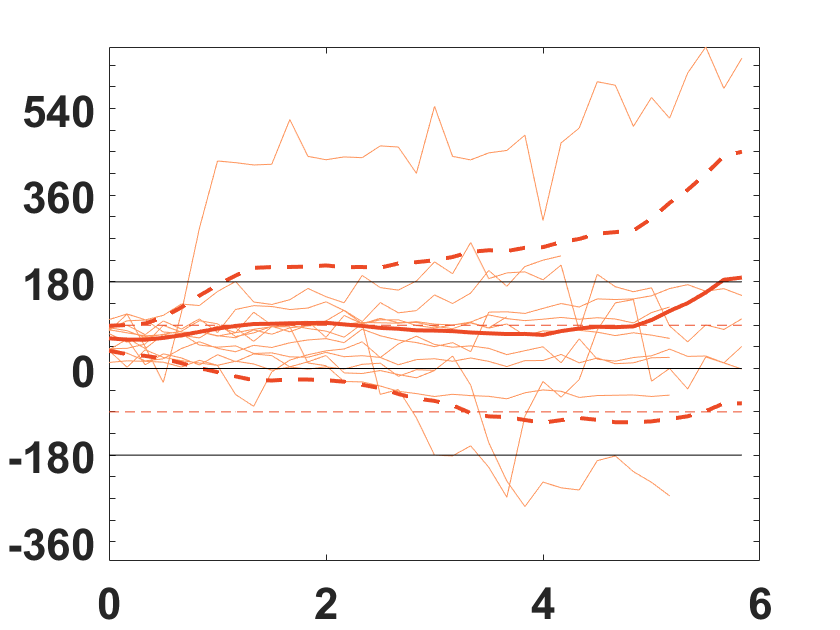

Supplement: Figure 5—source data 1. [file elife-69229-fig5-data1.zip › Figure5 Supp/Figure5f supp - static ctrl/STATIC_control_reorientation_O_22.png]

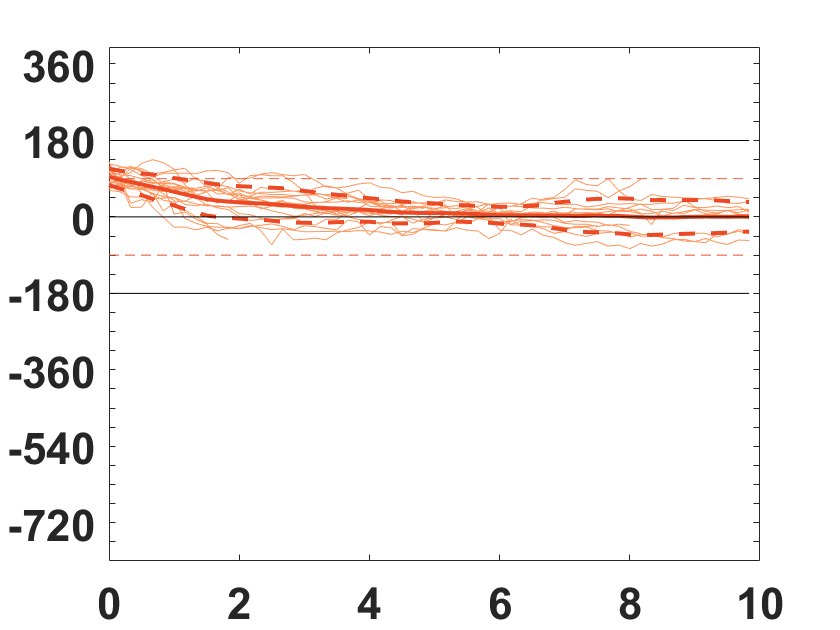

Supplement: Figure 5—source data 1. [file elife-69229-fig5-data1.zip › Figure5/5b/ANGLE_exp_reorientation_10h_O_22_big_box.png]

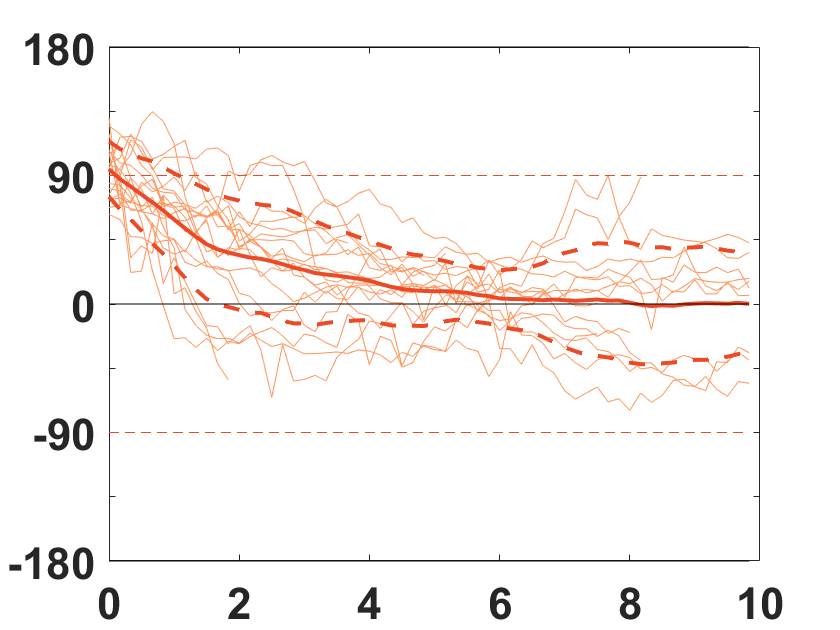

Supplement: Figure 5—source data 1. [file elife-69229-fig5-data1.zip › Figure5/5b/ANGLE_exp_reorientation_10h_O_22_box.png]

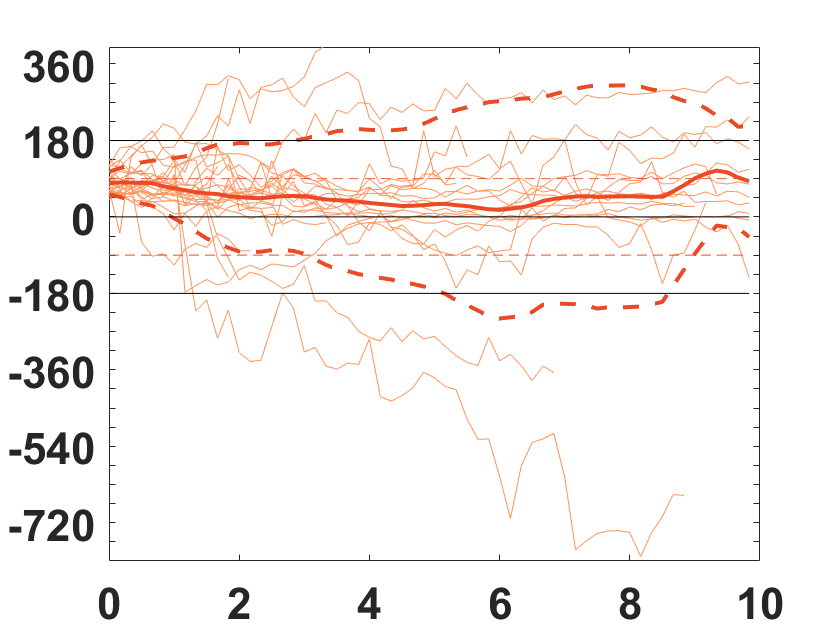

Supplement: Figure 5—source data 1. [file elife-69229-fig5-data1.zip › Figure5/5c/ANGLE_control_reorientation_graph_O_22_ALL.png]

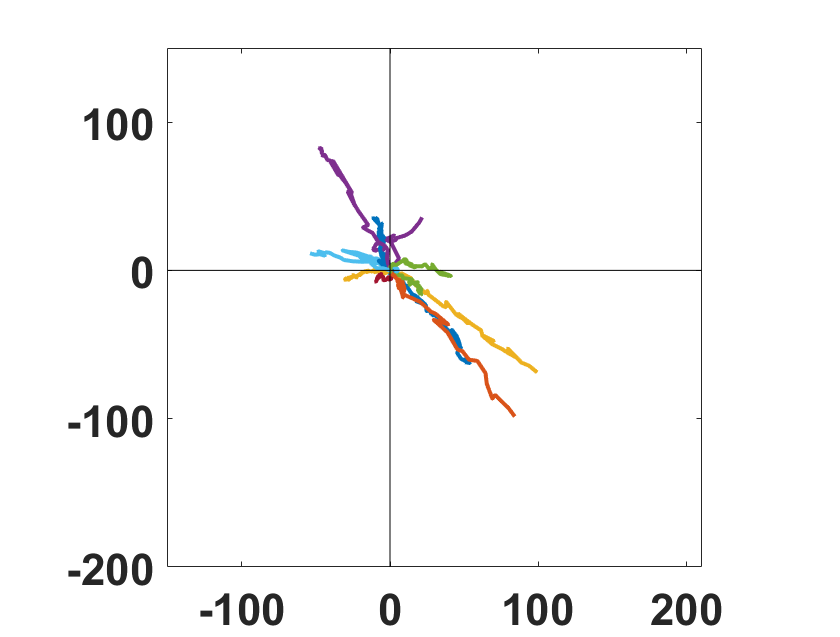

Supplement: Figure 5—source data 1. [file elife-69229-fig5-data1.zip › Figure5/5d/ANGLE_NZ_Opto_trajectories_40fr.png]

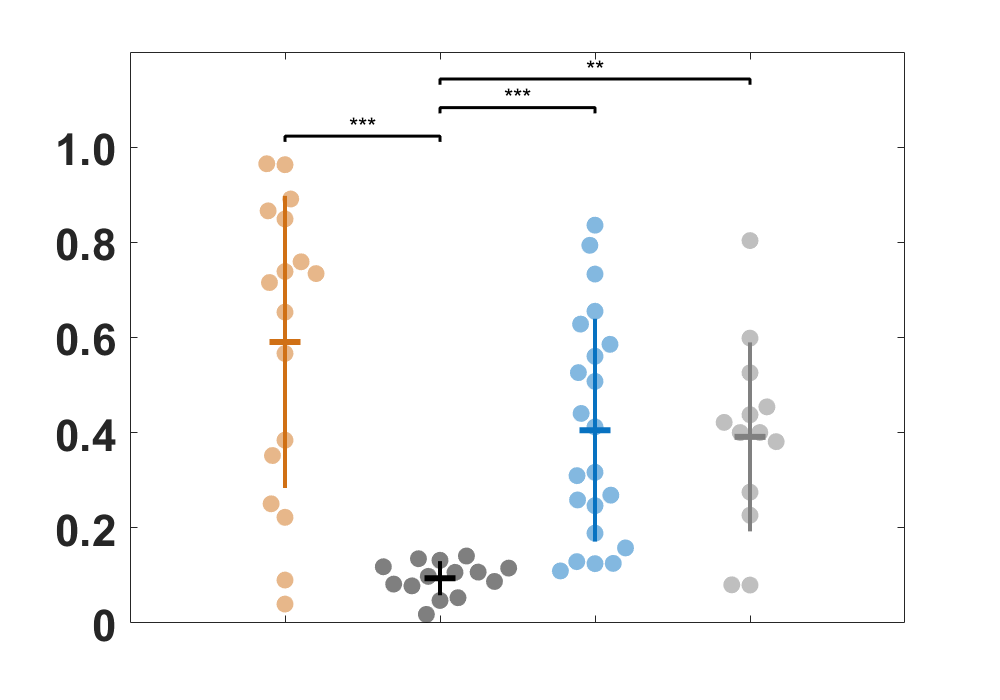

Supplement: Figure 5—source data 1. [file elife-69229-fig5-data1.zip › Figure5/5e/dirRatio_optoNZ_22_KW_Dunn_NZ.png]

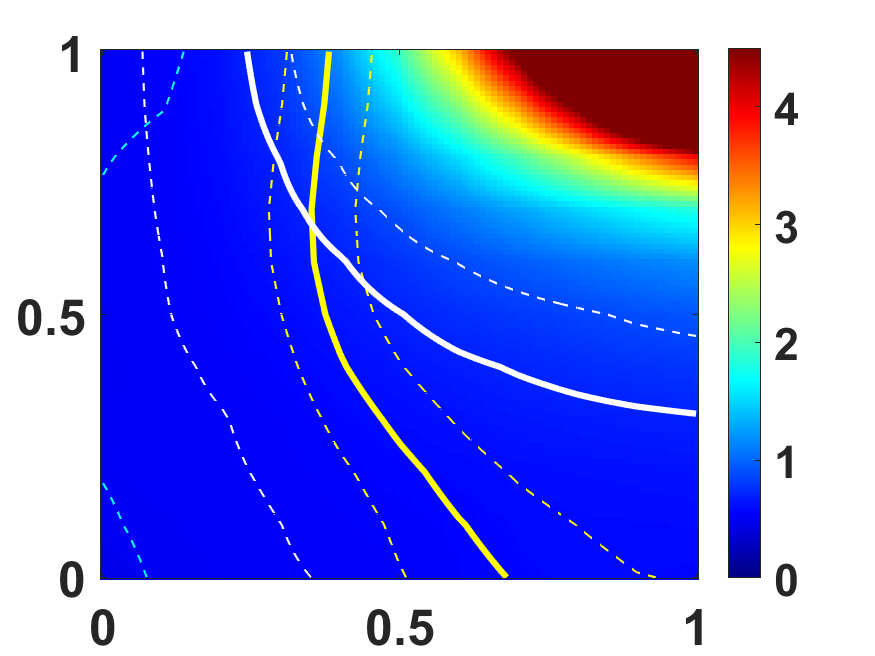

Supplement: Figure 6—source data 1. [file elife-69229-fig6-data1.zip › Figure6 Supp/Figure6 - figure supplement2/WTvsHeLa.png]

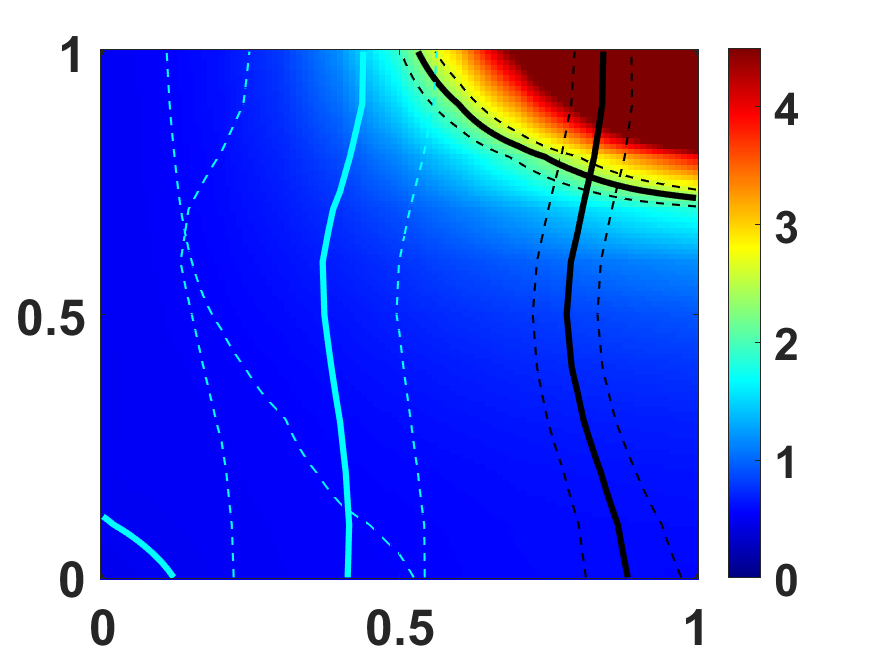

Supplement: Figure 6—source data 1. [file elife-69229-fig6-data1.zip › Figure6 Supp/Figure6 - figure supplement3 A-B/Protrusive unicity from experiments NZ/WTvsNZ.png]

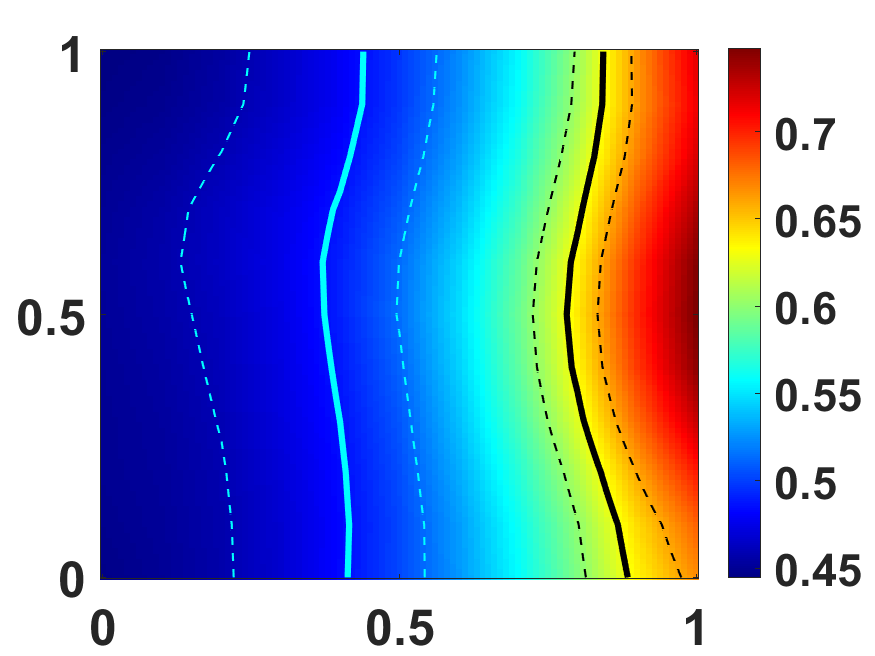

Supplement: Figure 6—source data 1. [file elife-69229-fig6-data1.zip › Figure6 Supp/Figure6 - figure supplement3 A-B/Protrusive unicity from experiments NZ/WTvsNZuni.png]

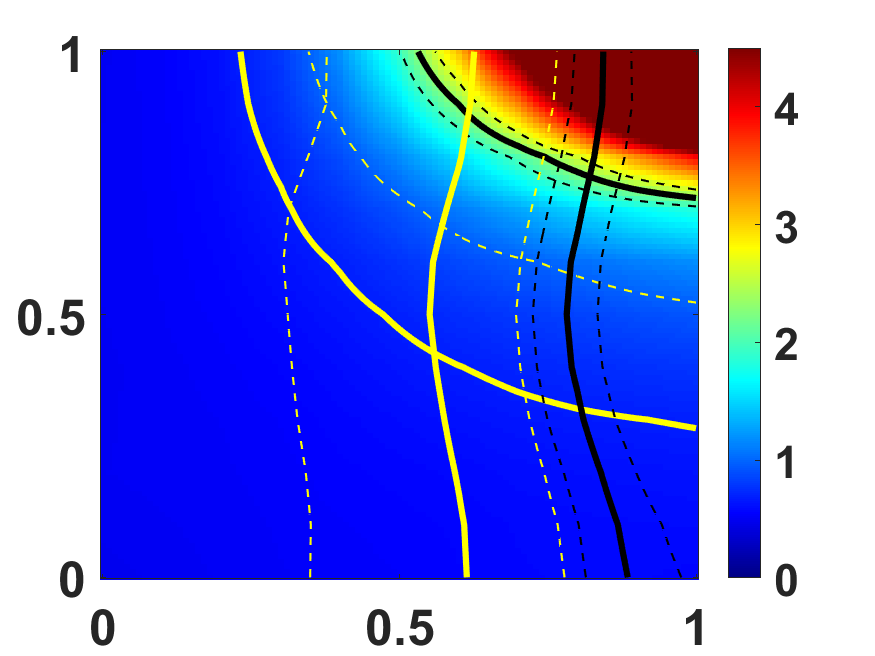

Supplement: Figure 6—source data 1. [file elife-69229-fig6-data1.zip › Figure6 Supp/Figure6 - figure supplement3 C-D/WTvsGCA.png]

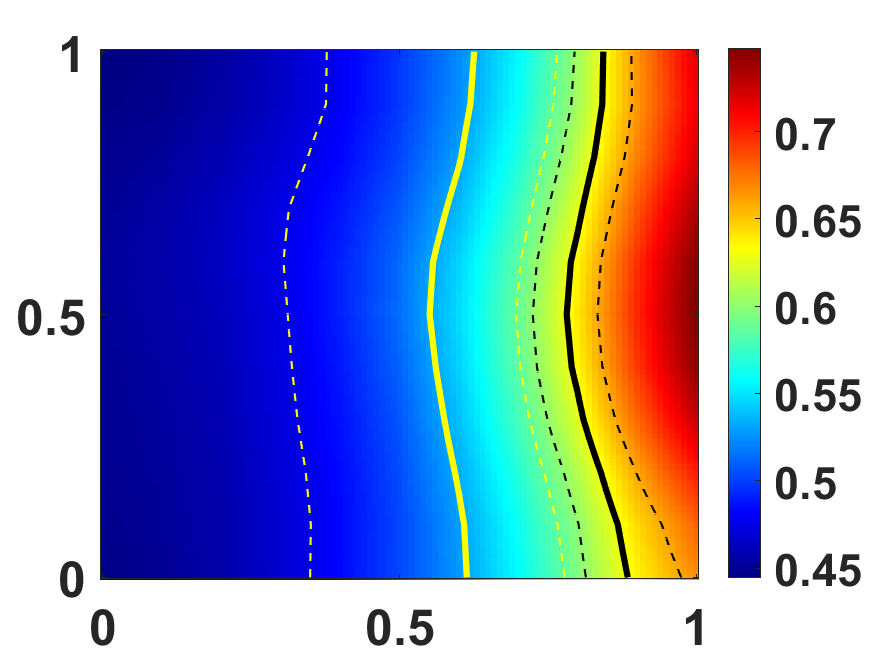

Supplement: Figure 6—source data 1. [file elife-69229-fig6-data1.zip › Figure6 Supp/Figure6 - figure supplement3 C-D/WTvsGCAuni.png]
